# Genome-wide association analysis uncovers rice blast resistance alleles of *Ptr* and *Pia*

**DOI:** 10.1038/s42003-024-06244-z

**Published:** 2024-05-20

**Authors:** Julian R. Greenwood, Vanica Lacorte-Apostol, Thomas Kroj, Jonas Padilla, Mary Jeanie Telebanco-Yanoria, Anna N. Glaus, Anne Roulin, André Padilla, Bo Zhou, Beat Keller, Simon G. Krattinger

**Affiliations:** 1 Department of Plant and Microbial Biology, University of Zürich, Zürich, Switzerland; 2 Research School of Biology, Australian National University, Canberra, ACT Australia; 3 International Rice Research Institute, Los Baños, Philippines; 4 PHIM Plant Health Institute, University of Montpellier, INRAE, CIRAD, Institut Agro, IRD, Montpellier, France; 5 Agroscope, Müller-Thurgau-Strasse 29, 8820 Wädenswil, Switzerland; 6 Centre de Biologie Structurale, CBS, University of Montpellier, CNRS UMR 5048, INSERM U, 1054 Montpellier, France; 7 Plant Science Program, Biological and Environmental Science and Engineering Division, King Abdullah University of Science and Technology (KAUST), Thuwal, 23955-6900 Saudi Arabia; 8 Center for Desert Agriculture, KAUST, Thuwal, 23955-6900 Saudi Arabia

**Keywords:** Genome-wide association studies, Agricultural genetics, Plant breeding, Plant immunity

## Abstract

A critical step to maximize the usefulness of genome-wide association studies (GWAS) in plant breeding is the identification and validation of candidate genes underlying genetic associations. This is of particular importance in disease resistance breeding where allelic variants of resistance genes often confer resistance to distinct populations, or races, of a pathogen. Here, we perform a genome-wide association analysis of rice blast resistance in 500 genetically diverse rice accessions. To facilitate candidate gene identification, we produce *de-novo* genome assemblies of ten rice accessions with various rice blast resistance associations. These genome assemblies facilitate the identification and functional validation of novel alleles of the rice blast resistance genes *Ptr* and *Pia*. We uncover an allelic series for the unusual *Ptr* rice blast resistance gene, and additional alleles of the *Pia* resistance genes *RGA4* and *RGA5*. By linking these associations to three thousand rice genomes we provide a useful tool to inform future rice blast breeding efforts. Our work shows that GWAS in combination with whole-genome sequencing is a powerful tool for gene cloning and to facilitate selection of specific resistance alleles for plant breeding.

## Abstract (web summary)

A GWAS on 500 genetically diverse rice accessions enables identification of an allelic series for the unusual *Ptr* rice blast resistance gene, and the *Pia* resistance locus.

## Introduction

Rice is an important staple crop, accounting for 18.9% of calories consumed globally^1^. One of the most serious threats for global rice production is rice blast, a disease caused by the filamentous fungal pathogen *Magnaporthe oryzae*^2^. Rice blast is responsible for significant crop losses, which if prevented could feed 60 million people each year^3^. Breeding for rice blast resistance remains the most effective and equitable means to limit rice production losses. The identification and cloning of rice blast resistance genes is an essential first step to deploy knowledge-guided disease resistance breeding strategies^4^.

Over 100 rice blast resistance loci have been genetically characterized and over 30 rice blast resistance genes have been cloned^5^. With the exceptions of *pi21*, encoding a proline-rich metal binding protein^6^, *Pi-d2*, encoding a B-lectin receptor kinase^7^, *Ptr*, encoding an armadillo repeat containing protein^8^, and *Pi65*, encoding a leucine-rich repeat receptor-like kinase^9^, rice blast resistance genes typically encode intracellular immune receptors of the nucleotide-binding domain leucine-rich repeat (NLR) protein family. Several NLR encoding rice blast resistance genes form allelic series, with various alleles at a given locus having different race-specificity spectra^10,11^. For example, seven alleles at the *Pik* resistance locus with varying resistance spectra have been identified^12,13^. *Pi1, Pik, Pik-m, Pik-p, Pik-s, Pik-h and Pike*^14–19^. The function of the *Pik* resistance locus is dependent on a pair of NLRs, one of which contains an integrated heavy-metal associated domain (HMA) responsible for the perception of the *M. oryzae* effector AVR-Pik^14,20^. Variation in the HMA domain of Pik is responsible for specific binding to different variants of AVR-Pik, resulting in broad or narrow effector specificity depending on the variant of Pik present^21^. Like *Pik*, the *Pia* locus functions through a pair of NLRs, RGA4 and RGA5, with RGA5 containing an integrated HMA domain^22^. In contrast to *Pik*, only two functional (resistance-conferring) *Pia* alleles have been identified so far. The HMA domains of described RGA5 variants directly interact with two sequence-unrelated *M. oryzae* effector proteins, AVR-Pia and AVR1-CO39^23^. While RGA5 acts as a ‘sensor’ NLR, RGA4 is a ‘helper’ NLR that is not involved in effector perception and instead initiates a cell death response after effector recognition by RGA5^24^.

*Pi-ta*, one of the first NLR resistance genes cloned in rice^25^, is described to interact directly with AVR-Pita based on yeast two-hybrid and in vitro binding assays^26^. A single amino acid polymorphism resulting in a serine to alanine substitution at residue 918 has been suggested to differentiate the functional Pi-ta version from non-AVR-Pita recognizing protein variants^25^. The *Pi-ta* locus is tightly linked to the rice blast resistance locus *Pi-ta2* in the centromeric region of rice chromosome 12^27,28^. The resistance spectrum of *Pi-ta2* was reported to be broader compared to *Pi-ta* and included all *M. oryzae* races to which *Pi-ta* containing rice is resistant, thus making it difficult to differentiate *Pi-ta* from *Pi-ta2* mediated resistance^29^. The development of near-isogenic lines in the blast-susceptible rice line LTH revealed that the *Pi-ta2* resistance spectrum is indeed broader and overlapping *Pi-ta*^30,31^. The blast resistance gene *Ptr* was mapped close to *Pi-ta* and *Pi-ta2* and confirmed to be a specific allele of the armadillo-repeat protein encoded by LOC_Os12g18729^8^. *Pi-ta2* was later shown to be allelic and encode a protein identical to the *Ptr* gene^32^. Interestingly, mutating *Ptr/Pi-ta2* results in susceptibility to *AVR-Pita* containing isolates, suggesting that *Ptr/Pi-ta2* is required for *AVR-Pita* mediated resistance^8,31^. To date, a single allele of *Ptr* has been functionally validated^8,31^.

Genebanks are an important source of novel resistance genes and allelic variants. However, screening large germplasm collections for agronomically useful traits and identifying the causal genetic loci has traditionally been a laborious and time-consuming process. Digital genebanks like the 3000 rice genomes project^33,34^ can expedite gene discovery, allowing trait data to be associated to genetic variations at the population level in the form of association studies. Genome-wide-association studies (GWAS) are an effective way to identify genetic loci by leveraging phenotypic and genotypic data from diverse populations without the need to establish bi-parental mapping populations. While several rice blast resistance loci have been identified using GWAS^35,36^, few studies have validated the underlying genetic cause of resistance, which can improve marker-assisted selection and ensure a unique source of resistance has been identified. Here, we show that GWAS coupled with medium-quality sequencing is an effective means to identify genetic loci, select candidate genes and allow for rapid functional validation using transgenic approaches.

## Results

### GWAS uncovers peak associations nearby the *Pia* and *Ptr* resistance loci

A diversity panel comprising 500 Asian rice (*Oryza sativa*) accessions was inoculated with six *M. oryzae* isolates from the Philippines. Isolates from the Philippines have previously been associated with pandemic status as the lineage is distributed globally^37^. To maximize the likelihood of discovering new resistance genes/alleles, the 500 rice accessions were selected from a larger diversity panel by excluding accessions with some known rice blast resistance genes (see methods). Disease severity was scored 7 days post inoculation (dpi) using a 0-5 scale, with 0 showing no symptoms and 5 showing large eyespot lesions with a diameter greater than 2 mm^38^. Accessions showing a standard deviation >1.3 across biological replicates were excluded from GWAS, leaving between 390 and 442 accessions with consistent disease severity scores per isolate infection (Supplementary Table 1). The average disease severity scores of the diversity panel followed a bimodal distribution for *M. oryzae* isolates Mo15-23, Mo15-24, and C923-49, while the disease scores were skewed towards susceptibility for isolates M64-1-3-9-1 and Mo15-125, and towards resistance for isolate IK81-25 (Supplementary Fig. 1).

**Fig. 1:**
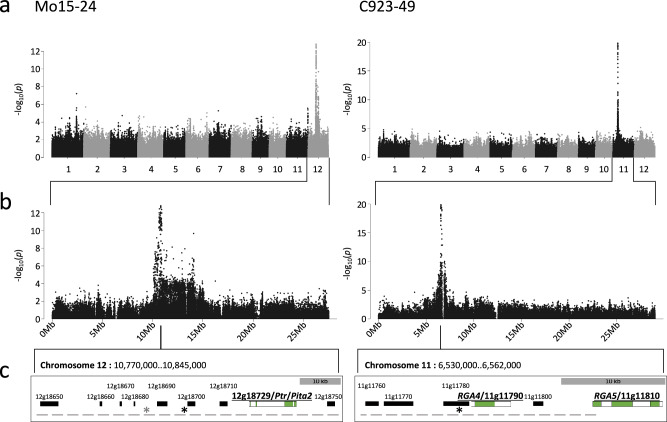
GWAS detect two strong rice blast resistance associations on chromosomes 11 and 12 near the Pia and Ptr resistance loci, respectively. **a** Manhattan plots showing associations between individual bi-allelic SNPs and resistance to *M. oryzae* isolates Mo15-24 (group two) and C923-49 across the twelve rice chromosomes. **b** Manhattan plots of chromosomes 12 (Mo15-24) and 11 (C923-49) from (**a**). **c** Close-up views of genomic regions of co-associated SNPs (haploblock) surrounding the peak SNP. The physical positions of the peak SNPs in the Nipponbare reference assembly for Mo15-24 (10,806,802), Mo15-23 (10,797,768), and C923-49 (6,540,075) are marked by asterisks (Mo15-23 peak SNP location is shown in gray). The linkage blocks surrounding the peak SNPs for Mo15-24 (Chr12: 10,769,576-10,845,095) and C923-49 (Chr11: 6,530,358-6,554,718) are marked by a gray dashed line. Gray scale bars represent 10 kb. Gene identifiers are represented as shortened versions of the Rice Genome Annotation (Osa1) Release 7 by removing *LOC_Os* from the start of each gene identifier. Exons and introns of known resistance genes are shown as green and white blocks, respectively.

Considering that the average disease severity scores showed a non-normal distribution (Supplementary Fig. 1), scores were converted to a binary format to allow for binomial treatment of the trait data when performing GWAS. Disease severity scores of <3 were classified as resistant, while disease severity scores ≥3 were considered susceptible. GWAS detected strong associations on rice chromosomes 11 and 12. The chromosome 11 peak was adjacent to the *Pia* resistance locus. While we detected the association on chromosome 11 for only one isolate (C923-49), similar association profiles were obtained on chromosome 12 for five of the six *M. oryzae* isolates (Fig. 1a, b, Supplementary Fig. 2). The peak associated SNPs (i.e., the SNPs with the lowest *P*-values) on chromosome 12 were located between positions 10.79 and 10.92 Mb (based on the Nipponbare reference assembly), close to the *Pi-ta* and *Ptr* resistance loci. A second peak on chromosome 12 was detected at position 12.9 Mb for three *M. oryzae* isolates, IK81-25, M64-1-3-9-1, and Mo15-125 (Supplementary Fig. 2). This association might correspond to a previously described rice blast resistance locus at 13.1 Mb^35^.

Given that similar associations on chromosome 12 were observed for multiple *M. oryzae* isolates, we sought to determine if these associations suggest a common source of resistance. For all peak SNP positions, a consistent pattern of association to a subset of resistant rice accessions could be observed, and the SNP variants associated with resistance were rarely present in susceptible accessions (<6.1%), except for the M64-1-3-9-1 and Mo15-125 peak associations (Supplementary Table 2, Supplementary Data 1). We refer to the subset of resistant rice accessions carrying peak associated SNPs as ‘associated accessions’. Two distinct groups of rice accessions that carry the chromosome 12 associations between positions 10.79 and 10.92 Mb could be observed, implying two distinct sources of resistance in the rice accessions tested (Supplementary Data 1). One group of *indica* rice accessions carried SNP variants associated with resistance to *M. oryzae* isolates IK81-25, M64-1-3-9-1 and Mo15-125 (group one), while the other group of accessions from the *indica*, *aus,* and *aro* subgroups carried SNP variants associated with resistance to *M. oryzae* isolates Mo15-23 and Mo15-24 (group two) (Supplementary Data 1). The Manhattan plot profile was also suggestive of two distinct groups of associated rice accessions, with the SNP association profiles overlapping between group one isolates, and a different profile observed for group two isolates (Supplementary Fig. 2). Resistance associations corresponding to group one isolates typically carried co-associated SNPs across the 10.79–12.9Mb region implying that this region, which spans the centromere, is tightly linked within group one resistance associated accessions (Supplementary Data 1, Supplementary Fig. 2). For all associations detected in this study, the Manhattan profile and peak associated SNPs was consistent for each isolate infection regardless of gaussian or binomial input data except in the case of Mo15-125 which produced a peak at 10’215’967 and 10’926’845 for binomial and gaussian input data, respectively. All group one and two isolates except for M64-1-3-9-1 are virulent to *Pi-ta* containing rice (Supplementary Table 3), suggesting that *Pi-ta* is not responsible for either of the two sets of resistance associations identified. All group one and two isolates except for Mo15-125 are avirulent to *Ptr* containing rice (Supplementary Table 3), indicating that *Ptr* may be responsible for the resistance associations to these isolates.

The peak SNP variant associated with resistance to C923-49 on chromosome 11 was enriched in resistant accessions, while being rarely present in susceptible accessions (Supplementary Table 2, Supplementary Data 2). The C923-49 resistance association is adjacent to the *Pia* locus (Fig. 1c) and the C923-49 isolate is avirulent to *Pia* (Supplementary Table 3), suggesting that *Pia* or an allele could be responsible for resistance to C923-49.

To narrow down the genomic regions likely to contain resistance genes on chromosomes 11 and 12, tightly linked SNPs surrounding peak associated SNPs were determined. Linkage blocks surrounding the peak associated SNPs for Mo15-24 (chromosome 12) and C923-49 (chromosome 11) spanned 75.5 kb and 24.4 kb, respectively (Fig. 1c). The resistance gene *LOC_Os12g18729* (*Ptr*) was located within the 75.5 kb linkage block on chromosome 12 (Fig. 1c). The only known functional allele of *Ptr* was previously shown to confer resistance to the *M. oryzae* isolate M64-1-3-9-1^32^, but this isolate was virulent on 47 out of 64 rice accessions that carry the group two resistance association (Supplementary Data 1). This indicates that the source of resistance in our study might be a novel allele of *Ptr* or a closely linked gene.

To account for the possibility that resistant rice accessions contain gene copy-number or presence-absence variation within the defined linkage blocks, we produced de novo assemblies of 10 rice accessions using linked-read sequencing. Three of the 10 accessions carried the group two resistance association (chromosome 12), two carried the group one resistance association (chromosome 12), and a single accession, IRIS_313-12190, carried both chromosome 12 peak SNP associations. Five of the rice accessions selected for sequencing carried the C923-49 resistance association on chromosome 11.

### Genome sequencing of resistance-associated rice accessions uncovers functional alleles of *Ptr* and *Pia*

Linked read sequencing (10X Genomics) was performed with a coverage of 60-fold or greater per rice accession. The scaffold N50s ranged from 0.78 – 4.6 Mb across the 10 rice accessions, with the total assembly sizes ranging from 411 to 428 Mb, except in the case of IRIS_313-10059 at 502 Mb. BUSCO (Benchmarking Universal Single-Copy Orthologs) scores between 97.7% and 98.4% indicated a high completeness of the 10 assemblies. (Supplementary Table 4).

Assessment of gene presence-absence polymorphisms in regions corresponding to the linkage blocks surrounding peak SNPs revealed no additional genes in the newly sequenced rice accessions compared to the Nipponbare reference, but amino acid (AA) polymorphisms and insertion/deletions in the putative promotor region and introns of *Ptr* were detected (Supplementary Figs. 3, 4, Supplementary Data 3). When querying the linkage block that partially includes the *Pia* locus, the five associated accessions carried genomic sequence corresponding to *RGA5*, while non-associated accessions carried genomic sequence corresponding to LOC_Os11g11810, a sequence divergent NLR present in the reference accession Nipponbare. Given that no additional genes, compared to the Nipponbare reference sequence, were detected within genomic sequence corresponding to the predicted linkage blocks, we decided to assess the sequence diversity of the candidate resistance loci *Ptr* and *Pia*. Genomic sequences encompassing the candidate resistance genes were extracted and multiple sequence alignments were performed, and the sequence of proteins encoded by *Ptr*, and the *Pia* genes *RGA4* and *RGA5* were extracted (Supplementary Figs. 3, 4, 6, 7, 8).

### The *Ptr* allele in group two (Mo15-23 and 24) resistance-associated accessions differs from the previously described functional allele of *Ptr*

Three of the sequenced rice accessions, IRIS_313-10314, IRIS_313-10738, and IRIS_313-8554, which showed the chromosome 12 resistance associations to group two isolates, all contained the same *Ptr* allele that differs from the previously described functional allele found in the rice cultivar Katy. In comparison to Katy Ptr, the new Ptr variant carried 43 AA substitutions, 14 AA deletions, and 4 AA insertions (Supplementary Figs. 3, 4). A single sequenced rice accession, IRIS_313-12190, carried chromosome 12 associations to both group one and group two isolates. The predicted Ptr protein sequence of IRIS_313-12190 shares homology with the group two resistance-associated accessions, except for four C-terminal AA substitutions, and three N-terminal AA substitutions (Supplementary Figs. 3, 4). Comparing the IRIS_313-12190 genomic sequence to other sequenced accessions suggests that a recombination event occurred somewhere in the genomic region corresponding to Ptr AA positions 136–187, resulting in a *Ptr* gene that carries a promotor and 5’ genic sequence similar to the allele found in group one associated accessions, with the remaining downstream sequence more similar to group two associated accessions (Supplementary Figs. 3, 4, Supplementary Data 3). This genomic recombination event may account for the presence of both chromosome 12 peak-associated SNPs in IRIS_313-12190, but does not necessarily imply broader resistance specificity.

Rice accessions IRIS_313-10059 and IRIS_313-10879, which carry strong resistance associations to group one isolates, carry *Ptr* alleles encoding for protein variants that differ from Katy Ptr by six and seven AA, respectively, and also differ from all other sequenced accessions (Supplementary Figs. 3, 4, Supplementary Data 4). In addition to *Ptr*, IRIS_313-10059 and IRIS_313-10879 were found to carry the reported *Pi-ta* resistance allele^25^, which is located ~200 kb away from *Ptr* at position 10.61 Mb on chromosome 12 (Supplementary Data 1). Unlike the group one resistance-associated accessions, the group two accessions do not carry the *Pi-ta* resistance allele. With the exception of the Ptr variant found in the group two resistance-associated accessions, the majority of AA polymorphisms differentiating Ptr variants in the 10 genomes we sequenced were located in the C-terminal region of the protein (Supplementary Figs. 3, 4). Considering the large number of AA changes encoded by the *Ptr* allele found in the group two resistance-associated accessions, we sought to validate the potential resistance function of this highly divergent allele.

### Transgenic plants containing the group two (Mo15-23 and 24) resistance-associated allele of *Ptr* are resistant to rice blast

To test if the group two resistance-associated allele of *Ptr* is sufficient to confer resistance to rice blast, the susceptible rice accession CO39, which contains the ‘non-functional’ Amane allele of *Ptr*, was transformed with a construct containing the *Ptr* allele amplified from the group two resistance associated rice accession IRIS_313-10314. The construct transferred into CO39 contained 10,090 bp of genomic sequence, including 2522 bp of native promotor sequence and 1016 bp of sequence downstream of the stop codon. Four-week-old plants were spray-inoculated with *M. oryzae* isolates Mo15-23 or Mo15-24. In all lines tested, non-transgenic sibling segregants were susceptible to rice blast infection while heterozygous or homozygous transgenic plants containing the group two *Ptr* allele, hereby referred to as *Ptr*_*b*_, were resistant (Supplementary Fig. 5).

### *Ptr* is a race-specific resistance gene with different functional alleles

**Fig. 2:**
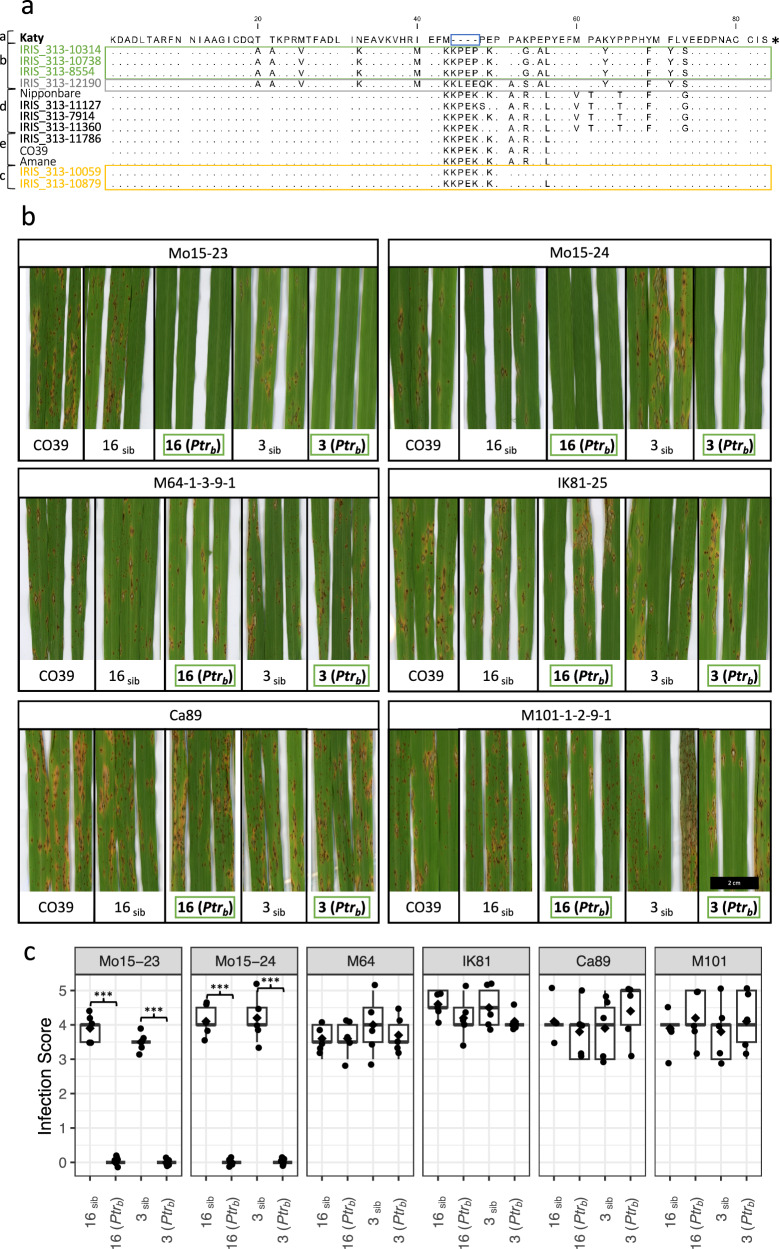
*Ptr*_*b*_ is a race-specific resistance allele. **a** Multiple sequence alignment of the polymorphic 79-83 C-terminal amino acids of Ptr variants with polymorphic residues relative to the Katy sequence (Ptr_a_) shown as single letter AA code with deletions shown as dashes and identical residues shown as dots. The Mo15-23 and Mo15-24 (group two) resistance-associated rice accessions are shown in green, the IK81-25, M64-1-3-9-1, and Mo15-125 (group one) resistance-associated rice accessions are shown in yellow, and the IRIS_313-12190 accession, which carried resistance associations to both group one and group two isolates at chromosome 12 is shown in gray. A 4 AA deletion that was previously reported to be diagnostic for Ptr_a_ is indicated by a blue box. The genotypes presented are classified into five variant subgroups (Ptr_a_ – Ptr_e_; indicated in letters a-e to the left of genotype names) based on the allelic diversity observed in 31 genetically diverse genotypes. **b** Four-week-old *Ptr*_*b*_ containing transgenic lines (3 and 16), their non-transgenic siblings (3_sib_ and 16_sib_), and the susceptible parental line CO39 were spray-inoculated with *M. oryzae* isolates indicated. Images of representative infected leaves were taken 7 days post-inoculation. Green boxes indicate transgenic plants containing *Ptr*_*b*_. Scale bar = 2 cm. **c** Infection scoring data from five plants each of 4-week-old *Ptr*_*b*_ containing transgenic lines (3 and 16) and their non-transgenic siblings (3_sib_ and 16_sib_). Scoring data at 7 days post inoculation is represented by box plots where diamonds indicate the average infection score for each genotype. Significant differences between sibling lines infected with each isolate, as determined by a two-sample t-test, are indicated (*** = *P* < 0.001), *n* = 5 plants.

After confirming that resistance is conferred by *Ptr*_*b*_, we tested if the *Ptr*_*b*_ resistance spectrum extended to other *M. oryzae* isolates. Spray inoculations were performed for six isolates with a diverse virulence spectrum (Supplementary Table 3). The *Ptr*_*b*_ transgenic lines showed complete resistance to Mo15-23 and Mo15-24 and were susceptible to the other rice blast isolates tested, consistent with a typical qualitative, major resistance gene response (Fig. 2). *Ptr*_*b*_ susceptibility to IK81-25 and M64–1–3-9-1 is consistent with the GWAS results and the separation of group one and two resistance associated accessions. The Katy allele of *Ptr* (*Ptr*_*a*_) in the NIL IRBLta2-Re (A NIL in the CO39 background) was previously shown to confer resistance to *M. oryzae* isolates IK81-25, M101–1–2-9-1 and M64–1–3-9-1^32^, all of which are virulent on transgenic lines carrying the *Ptr*_*b*_ allele (Fig. 2). To confirm that *Ptr*_*a*_ specificity is not the result of a tightly linked gene in the NIL, we performed infection testing with two *Ptr*_*a*_ mutants^32^. Both mutants were susceptible to Mo15-23 and M64–1–3-9-1 while their parental line, IR64, was resistant. In summary, both *Ptr*_*a*_ and *Ptr*_*b*_ alleles confer resistance to Mo15-23, while only *Ptr*_*a*_ is able to confer resistance to M64–1–3-9-1 (Supplementary Table 5). These results confirm that alleles of *Ptr* confer resistance to different isolates of *M. oryzae*.

### Comparisons between Ptr amino acid sequence and allele profiling of 3000 rice genomes uncovers significant diversity

**Fig. 3:**
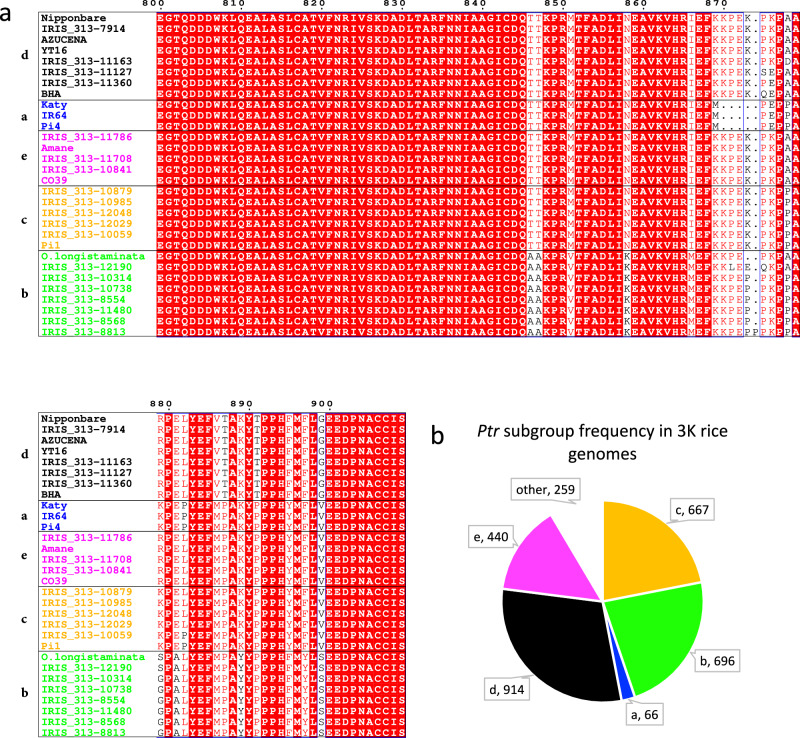
Ptr C-terminal amino acid alignment differentiates Ptr variants into five distinct subgroups. **a** Amino acid sequence extracted from 30 genomic sequences including; the 10 accessions sequenced here, BHA, YT16, Pi1, Pi4, Amane, and Katy^8^, CO39^32^, Nipponbare^39^, *O. longistaminata* and 12 other diverse rice genomes^40^. A full protein sequence alignment can be found in Supplementary Fig. 4. Variant subgroups are labeled a-e with accessions in each subgroup color coded. Accessions containing the Ptr variant encoded by *Ptr*_*a*_ are colored blue. Accessions with high similarity to the Ptr variants encoded by *Ptr*_*b*,_ and the *Ptr*_*c*_ resistance association are colored green and yellow, respectively. **b** Accessions represented in the 3000 rice genomes project were profiled based on non-synonymous SNPs which correspond to amino acid changes differentiating the subgroups in (**a**). Subgroups can be differentiated by the combination of amino acid changes at alignment positions 879 and 899 and by the characteristic deletion found in *Ptr*_*a*_. 259 accessions could not definitively be placed in the different variant subgroups due to missing SNP data or heterozygous base calls.

After confirming a new, functionally distinct *Ptr* allele, we sought to compare the sequence variants of *Ptr* we identified to those present in other sequenced accessions. Comparison of the AA sequence of Ptr from the 10 accessions we sequenced with those of BHA, YT16, Pi1, Pi4, Amane, and Katy^8^, CO39^32^, Nipponbare^39^, *O. longistaminata* and 12 other diverse rice genomes^40^ identified 13 different intact protein variants of Ptr (Fig. 3, Supplementary Fig. 4, Supplementary Data 4). We classified these variants into 5 subgroups based on verified resistance function (Ptr_a_ and Ptr_b_), GWAS resistance association (Ptr_c_), and overall sequence homology (Fig. 3, Supplementary Fig. 4, Supplementary Data 4). The Ptr_a_ subgroup is defined by a loss of four AA close to the C-terminus of the protein, a differentiating feature indicated during the first classification of Ptr function^8^ (Figs. 2a, 3). While all other variant groups are also defined by polymorphisms in the C-terminus of the protein (Figs. 2a, 3), the Ptr_b_ subgroup contains additional AA changes throughout the protein (Supplementary Figs. 3, 4). The variant subgroup c containing the Ptr variants associated with the Ik81-25, M64-1-3-9-1, and Mo15-125 resistance GWAS peak are tentatively named Ptr_c_ (Fig. 3, Supplementary Fig. 4). The two remaining variant subgroups (d and e) contained accessions with similarity to either Nipponbare (subgroup d), or CO39 (subgroup e) (Fig. 3, Supplementary Fig. 4).

A high density of SNP calls are available in coding region of *Ptr* for the vast majority of the 3000 rice genomes, thus allowing for allele profiling based on non-synonymous changes that co-associate with group two (*Ptr*_*b*_) resistance-associated accessions, group one resistance associated accessions (*Ptr*_*c*_), or the previously described Katy *Ptr* allele (*Ptr*_*a*_). We further profiled the 3000 rice genomes for non-synonymous SNPs leading to amino acid changes associated with the e and d variant subgroups. Surveying the 3000 rice genome SNP data revealed that 696 of the 3024 rice accessions likely carry *Ptr*_*b*_ or a similar variant, 667 accessions carry alleles similar to *Ptr*_*c*_, 66 accessions appear to carry *Ptr*_*a*_, and 914 and 440 accessions are similar to the variant d and e subgroups, respectively (Fig. 3b, Supplementary Data 5). This haplotype analysis indicates that *Ptr*_*a*_ is comparatively rare in the rice gene pool. The allele profiling results we provide (Supplementary Data 5) give breeders and researchers a useful tool to identify, functionally validate, and deploy diverse alleles of *Ptr* in agronomically relevant accessions.

### Rice accessions with the chromosome 11 resistance association at the *Pia* locus carry variable alleles of *Pia* resistance genes *RGA4* and *RGA5*

Five of the 10 accessions for which de novo assemblies were obtained carried the chromosome 11 resistance association at the *Pia* locus. Of these, three carried an RGA4 protein version that was identical to the previously described functional version from the rice cultivar Sasanishiki^22^, and two carried an RGA4 variant with 66 AA changes relative to Sasanishiki (Supplementary Figs. 6, 7, Supplementary Data 4). This polymorphic variant of RGA4 is more similar to the variant found in the five non-associated accessions than to the variant found in other associated accessions (Supplementary Figs. 6, 7, Supplementary Data 4). A leucine at Sasanishiki AA position 671 of RGA4 is common among all associated accessions and is replaced by a serine in non-associated accessions containing a full-length version of RGA4 (Supplementary Fig. 7).

Sequence homologous to *RGA5* was only detected for the five C923-49 resistance-associated accessions (Supplementary Fig. 6). Non-associated accessions instead carried genes homologous to LOC_Os11g11810, which only shares homology to *RGA5* in the 5’ genic region encoding the first 148 amino acids. Besides IRIS_313-10059 *RGA5*, which encodes an RGA5 identical to Sasanishiki, the RGA5 encoded in rice accessions IRIS_313-10314 and IRIS_313-11786 contain 12 AA changes relative to Sasanishiki, including 9 within the HMA domain (Supplementary Figs. 6, 8, 9). The AA sequence of the IRIS_313-10314 and IRIS_313-11786 RGA5 is similar to the accession 93-11^41^ except for a 3 AA insertion in the HMA domain (Supplementary Figs. 6, 8, 9). IRIS_313-12190 has 54 AA changes relative to the Sasanishiki RGA5, including 13 within the HMA domain (Supplementary Figs. 6, 8, 9). IRIS_313-11360 features a similar AA sequence to IRIS_313-12190 except for a C-terminal frameshift mutation resulting in a premature stop codon and 42 AA missing from the C terminus of RGA5 (Supplementary Figs. 6, 8). This C-terminal frameshift is located immediately adjacent to the HMA domain.

Together, these results indicate that the presence/absence of the ‘sensor’ *NLR RGA5* determines rice blast resistance at the *Pia* locus, and extensive sequence variation exists in both *RGA4* and *RGA5*.

### Alleles at the *Pia* locus encode variable HMA domains of RGA5 and differ from described alleles

**Fig. 4:**
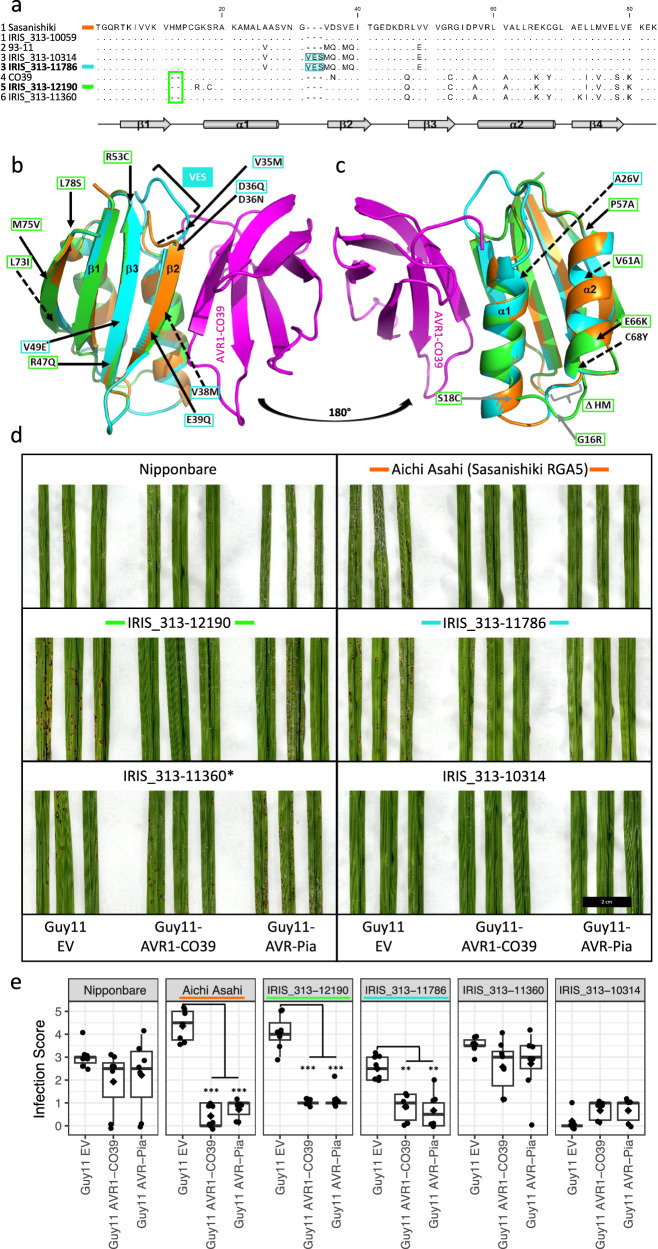
C923-49 resistance-associated accessions carry amino acid changes in the integrated HMA domain of RGA5 that lead to predicted changes in AVR interaction surface without altering AVR specificity. **a** Amino acid alignment of the RGA5 HMA domains from known functional (Sasanishiki and CO39), or likely functional (93-11) resistance protein versions, and the C923-49 resistance associated accessions. HMA variants are numbered 1–6 next to genotype name with newly identified functional variants shown in bold. Secondary structural elements are shown beneath the sequence alignment. Conserved residues relative to Sasanishiki are indicated by dots. Accession labels, deletions, and insertions are color coded to match their respective models in (**b**, **c**). IRIS_313-11360* contains a frameshift mutation after the HMA domain of RGA5. **b**, **c** Views of the interface between HMA•AVR1-CO39 models from opposite sides. Cartoon representation of three HMA•AVR1-CO39 models involving RGA5_Sasanishiki (orange), IRIS_313-11786 (cyan), IRIS_313-12190 (green), and AVR1-CO39 (magenta). Polymorphisms are indicated from the position numbering of the sequence alignment in (**a**) and are reported in the structural models by black arrows, plain for surface exposed, and dashed for buried or partially buried positions. A26V corresponds to the replacement of A26 of RGA5_Sasanishiki by a valine residue in a buried position. The last E80K polymorphism is not shown in the structure models, which end at V79. The H12 M13 insertion and the G16R or S18C polymorphisms are shown by gray bracket and arrows, respectively. **d** Infection testing of C923-49 resistance associated accessions with transgenic *M. oryzae* isolates expressing AVR1-CO39, AVR-Pia or carrying an empty vector (EV). Colored lines either side of accession names indicate the HMA domain sequence aligned in (**a**) and modeled in (**b**/**c)**. **e** Infection scoring data from six or seven 3-week-old plants for each genotype, per infection treatment, except for IRIS_313-10314 (*n* = 5). Scoring data at 7 days post inoculation is represented by box plots where diamonds indicate the average infection score for each genotype. Significant differences between AVR expressing Guy11 isolates and the empty vector (EV) control as determined by a two-sample t-test, are indicated (*** = *P* < 0.001, ** = *P* < 0. 01). Scale bar = 2 cm. Additional representative infected leaves are presented in Supplementary Fig. 10. Infection results are summarized in Supplementary Table 7.

The integrated HMA domain of RGA5 binds the *M. oryzae* effector proteins AVR1-CO39 and AVR-Pia, and is required for resistance to *M. oryzae* isolates containing these effectors^23^. Therefore, we compared the amino acid sequences of the HMA domain between the C923-49 resistance-associated accessions and the known functional RGA5 variants from Sasanishiki and CO39. Three additional HMA variants were detected. Variant 3 present in rice accessions IRIS_313-10314 and IRIS_313-11786 differs from Sasanishiki by nine AA, including a three AA insertion (VES) (Fig. 4a). Besides the three AA insertion, variant 3 is otherwise homologous to the HMA domain of the 93-11 allele (variant 2) (Fig. 4a). The Pi60(t) resistance gene from 93-11 was mapped to a region containing *RGA4* and *RGA5*, and 93-11 was also shown to be resistant to *AvrPia* containing isolates of *M. oryzae*^42^. In addition, protoplasts from the accession Peh-kuh-tsao-tu, which contains the same *Pia* allele as 93-11, were shown to elicit cell death responses when transiently expressing *AVR-Pia*^22^. Together these results suggest that the 93-11 allele of *RGA5* is functional. The two remaining C923-49 resistance-associated rice accessions, IRIS_313-12190 (variant 5) and IRIS_313-11360 (variant 6) contain RGA5 HMA variants similar to CO39 from which they differ by four and two amino acid changes, respectively (Fig. 4a).

To determine if any of the polymorphisms present in the HMA domains of the C923-49 resistance associated accessions are likely to result in changes to the interaction interface between the HMA domain and the detected effectors, we modeled the variable HMA domains from IRIS_313-11786 (variant 3) and IRIS_313-12190 (variant 5), based on the structure of the Sasanishiki HMA domain bound to AVR1-CO39^43^ (Fig. 4b, c). For the IRIS_313-11786 (variant 3) HMA-AVR1-CO39 complex the model suggested an increase of the binding interface surface area due to additional interacting residues between the N-terminus of AVR1-CO39 and residues in the re-modeled loop between the first helix and the second strand of the HMA domain, but this does not necessarily indicate a stronger resistance response would take place. In accordance with the increased binding interface, the predicted free binding energy of the IRIS_313-11786 HMA-AVR1-CO39 complex is lower (−7.9 Δ^i^G kcal/mol) compared to the Sasanishiki complex (−4.7 Δ^i^G kcal/mol) (Supplementary Table 6). Importantly, the re-modeling of this loop is completely compatible with the interface of the complex and does not introduce any clash. Two exposed residues in the variant 3 interaction surface at positions D36Q and E39Q differ from the Sasanishiki HMA domain, but are also present in the likely functional HMA variant from 93 to 11 (Fig. 4) and do not significantly alter the predicted free binding energy of the 93-11 HMA-AVR1-CO39 compared to Sasanishiki (Supplementary Table 6). All remaining amino acid changes in the HMA domain of IRIS_313-11786 are predicted to be buried or are not located at the interaction surface. The IRIS_313-12190 HMA domain (variant 5), which carries a two AA deletion after position 11, also carries two nearby AA substitutions, G16R and S18C. Together with the deletion, these substitutions may influence the structure or positioning of the first alpha helix, which is involved in the HMA-AVR interaction (Fig. 4a, b, c). However, the predicted binding energy of the HMA-effector complex is similar for IRIS_313-12190 (−5.1 Δ^i^G kcal/mol) and Sasanishiki (−4.7 Δ^i^G kcal/mol) HMA domains indicating that the perturbations at the complex interface are marginal (Supplementary Table 6).

### Infection testing of C923-49 resistance-associated accessions with transgenic *M. oryzae* isolates indicate that two new *Pia* alleles also elicit an effector-triggered immune response in the presence of AVR1-CO39 or AVR-Pia

To determine if the C923-49 resistant rice accessions with polymorphic RGA5 HMA domains, perceive AVR1-CO39 and AVR-Pia, infection tests were performed with the *M. oryzae* isolate Guy11 transformed with an empty vector construct (EV), AVR1-CO39^44^ or AVR-Pia^45^. Nipponbare, which does not contain a functional *Pia* locus, was susceptible to all three isolates of Guy11 (Fig. 4d, e, Supplementary Fig. 10). Aichi Asahi, which contains the Sasanishiki alleles of RGA4 and RGA5, was susceptible to Guy11 EV, but resistant to Guy11 containing AVR1-CO39 or AVR-Pia (Fig. 4d-e, Supplementary Fig. 10). Of the two accessions that have RGA5 HMA domains similar to CO39 (variant 5), IRIS_313-12190 was resistant to AVR1-CO39 and AVR-Pia as indicated by small lesions compared to Guy11 EV infections, while IRIS_313-11360 was susceptible to all Guy11 transformants (Fig. 4d, e, Supplementary Fig. 10). The susceptible reaction of IRIS_313-11360 is likely caused by a frameshift mutation which is present just after the HMA domain and leads to a truncated RGA5 protein (Supplementary Fig. 6, 8), however we cannot discount the possibility that reduced expression or some other mechanism is responsible for susceptibility. IRIS_313-10314 which possesses variant 3 of the HMA domain was resistant to Guy11 EV meaning the response to AVR1-CO39 or AVR-Pia could not be determined (Fig. 4, Supplementary Fig. 10). However, IRIS_313-11786, which also contains HMA variant 3, was susceptible to Guy11 EV and resistant to Guy11 containing AVR1-CO39 or AVR-Pia (Fig. 4d, e). In summary, the HMA domains of both IRIS_313-11786 (variant 3) and IRIS_313-12190 (variant 5) are able to recognize AVR1-CO39 and AVR-Pia. The resistance responses in IRIS_313-12190 also suggests that its divergent form of RGA4, which differs from Sasanishiki RGA4 by 66 AA (Supplementary Fig. 7), is a functional variant. Interestingly, several non-associated accessions carry intact versions of *RGA4*, which are similar to the functional IRIS_313-12190 *RGA4* allele. Comparing RGA4 in non-associated accessions reveals that they encode 6 AA substitutions between alignment positions 55-63 and a single AA substitution at alignment position 671 (Supplementary Fig. 7). Every genotype sequenced regardless of the presence of *RGA5*, contains an *RGA4* allele encoding the auto-active MHD motif variants TYG or MYG (Supplementary Fig. 7), either of which is sufficient for RGA4 mediated cell death^24^. Interestingly, IRIS_313-12190 features a naturally occurring MYG auto activity variant of the MHD motif which was previously shown to be sufficient for RGA4 cell death activity when transiently expressed in *N.* *benthamiana*^24^.

**Fig. 5:**
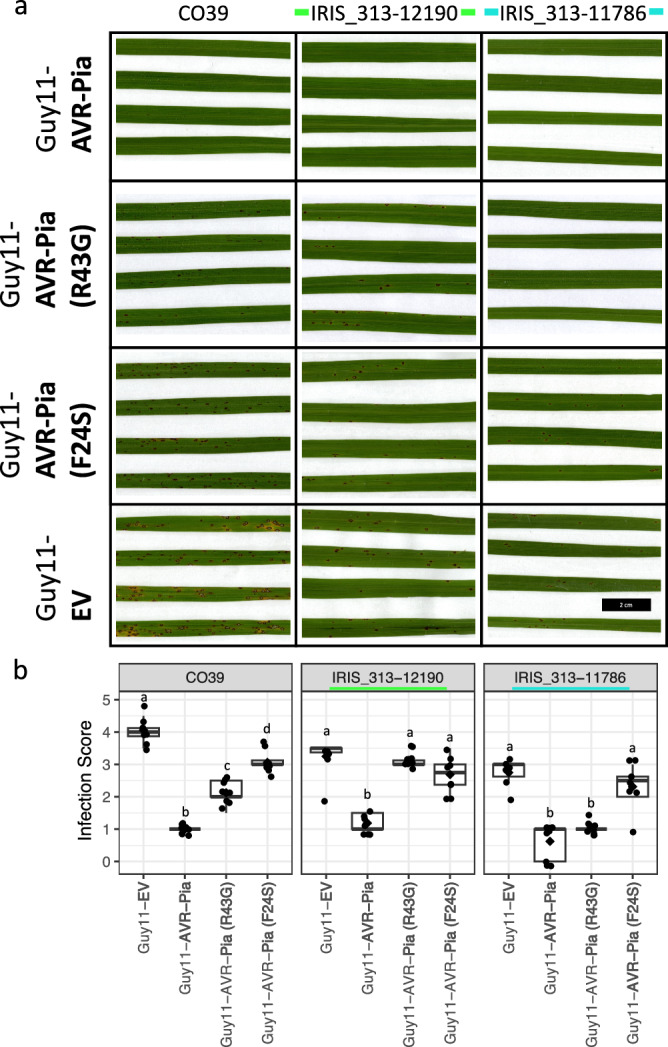
HMA domain variants respond differently to AVR-Pia amino acid changes which give rise to the virulent AVR-Pia-H3. **a** Infection testing of RGA5 HMA domain variant containing accessions with transgenic *M. oryzae* isolate Guy-11 expressing AVR-Pia, AVR-Pia (R43G), AVR-Pia (F24S) or carrying an empty vector (EV). Scale bar = 2 cm. **b** Infection scoring data from eight 3-week-old plants for each genotype, per infection treatment, except for IRIS_313-11786 infected with Guy11-EV (*n* = 6). Scoring data at 7 days post inoculation is represented by box plots diamonds indicate the average infection score for each genotype. Groups which do not differ significantly from each other based on a 95% confidence interval are labeled with the same letter (one-way ANOVA, Tukey’s HSD). Colored lines either side of accession names indicate matching HMA domain sequence aligned in Fig. 4a.

Given the observed AA changes in the RGA5 HMA domains of both IRIS_313-11786 (variant 3) and IRIS_313-12190 (variant 5), we sought to determine if these changes altered the specificity to *M. oryzae* isolates IN017 and IN058 which carry the AVR-Pia-H3 allele and are virulent on rice accessions carrying the *Pia* resistance locus from Sasanishiki or CO39^23^. IN017 was virulent on IRIS_313-12190 and IN058 was virulent on IRIS_313-11786 indicating that AVR-Pia-H3 does not elicit an effector-triggered immune response in either accession (Supplementary Fig. 11). When independently testing the influence of the R43G and F24S AA polymorphisms which differentiate AVR-Pia-H3 from Avr-Pia, IRIS_313-12190 (variant 5) was susceptible to both mutant versions of AVR-Pia (Fig. 5). However, IRIS_313-11786 (variant 3) showed a similar level of resistance to Guy11 expressing AVR-Pia (R43G) as to Guy11 expressing AVR-Pia, but was susceptible to Guy11 expressing AVR-Pia (F24S) (Fig. 5). These results are interesting in the context of plant-pathogen co-evolution; where strong selective pressure is placed on plant-perceived effectors like AVR-Pia. While the complete loss of dispensable effectors is common, effector mutations represent an alternative path for pathogens to evade effector-triggered immune responses. These results imply a possible evolutionary path for the selection of single AA changes in AVR-Pia whereby (R43G) could give rise to improved pathogen performance until encountering RGA5 HMA variant 3 which would strongly select for the F24S mutation. This hypothesis is further supported by the observed partial reduction in resistance to Guy11 expressing AVR-Pia (R43G) in CO39 (variant 4) (Fig. 5), and Kitaake^45^ (variant 1) which would also select for to complete evasion conferred by the further addition of the F24S mutation.

### *Pia* appears most commonly in indica rice accessions

Identification of *Pia* haplotypes in the 3000 rice genomes project^33^ was limited by the fact that homology to the reference Nipponbare genome, on which the SNP data is based, is only maintained in the first 148 AA of RGA5. Still, it was possible to assign 9 associated non-synonymous polymorphisms to 1050 accessions that are likely to encode RGA5 rather than the sequence divergent LOC_Os11g11810 found in Nipponbare (Supplementary Data 2). Comparing the two new functional HMA domain variants of RGA5 we identified to available de-novo genome assemblies shows that these variants are not restricted to one subgroup of rice. The variant 5 form was detected in both *indica* and *japonica* rice, while variant 3 was only detected in *indica* rice accessions (Supplementary Data 4). All of the accessions we compared that did not contain RGA5 were *japonica* rice accessions. Indeed, SNP profiling across *RGA5/LOC_Os11g11810* for the 3000 rice genomes suggests that *RGA5* appears more commonly in *indica* rice than in *japonica*. In total, 966 of the 1254 accessions that show an *RGA5*-like haplotype are *indica* accessions, whereas only 823 of the remaining 1770 rice accessions that do not show an *RGA5* haplotype were *indica* accessions. The higher prevalence of the *RGA5* haplotype in *indica* rice may suggest an introduction early in the domestication of *indica* rice, consistent with the single origin of *indica* rice from the intercrossing of *japonica* rice and a wild rice relative^46^.

## Discussion

In this study, we present a rapid strategy to identify and validate rice blast resistance genes following GWAS. Key to this achievement was the generation of medium-quality assemblies of resistant rice accessions from our diversity panel that carried peak associated SNPs. Disease resistance genes are often located in highly dynamic regions of the genome, where gene copy number and presence-absence variations are frequent. Relying on reference assemblies that do not contain the gene of interest bears the risk of missing new functional alleles^47,48^. The critical step of generating assemblies from the GWAS panel maximized the likelihood of identifying functional alleles for validation. Performing GWAS using sequenced and well-maintained populations, like the 3000 rice genomes project, allows for profiling of all accessions based on the presence of SNPs likely to be associated with particular resistance alleles. This greatly enhances the capability of breeding programs and prevents investment into the breeding, mapping or validation of resistance genes that have already been described.

We identified a resistance-conferring *Ptr* allele, designated *Ptr*_*b*,_ indicating that *Ptr* forms an allelic series with multiple functional alleles. Allelic series with multiple functional alleles exhibiting various recognition spectra to various pathogen isolates are common for NLR-encoding genes^13,49^. However, few allelic series for non-NLR encoding genes have been described. In maize, various alleles of a maize wall-associated kinase gene confer resistance against the fungal Northern corn leaf blight disease^50^. The wheat powdery mildew resistance gene *Pm4*, encoding an atypical kinase-MCTP protein, has two known functional alleles^51^. *Ptr* represents an additional example of an allelic series for an atypical disease resistance gene. The discovery of *Ptr*_*b*_ in this study adds valuable biological insight into the possible function of this atypical disease resistance gene, indicating that Ptr might be involved in the direct or indirect perception of *M. oryzae* effectors consistent with its qualitative resistance response and race-specificity.

In addition to *Ptr*_*b*_, we identified an additional *Ptr* allele, tentatively designated *Ptr*_*c*_, that was found to be tightly linked to an IK81-25, M64-1-3-9-1 and Mo15-125 resistance association. Mo15-125 was previously shown to be virulent on *Ptr*_*a*_ containing rice^32^, but Mo15-125 was avirulent on the majority of rice accessions carrying the IK81-25, M64-1-3-9-1 and Mo15-125 resistance association at the *Ptr*_*c*_ locus. This suggests that *Ptr*_*c*_ could confer resistance to *M. oryzae* isolates that are virulent on both *Ptr*_*a*_ and *Ptr*_*b*_. In support of the conclusion that other *Ptr* alleles might be novel sources of rice blast resistance, a rice blast resistance QTL from the weedy rice ecotype BHA which confers a resistance distinct from *Pi-ta* and *Ptr/Pi-ta-2* (*Oryza* spp.) was mapped to a 186,137 bp region on chromosome 12 that spans *Ptr*^52^. Recently, a unique *Ptr* allele was shown to be present in the weedy rice donor “BHA” suggesting that this allele of *Ptr* or a nearby gene is responsible for conferring blast resistance^53^. BHA *Ptr* encodes a similar protein to subgroup d accessions like Nipponbare, but with AA changes in the C-terminal region that could imply unique specificity (Fig. 3, Supplementary Fig. 4). The *Pi57(t)* rice blast resistance gene introgressed from the wild rice *Oryza longistaminata* was mapped to a 51.7 kb region which includes *Ptr*^54^. The available genomic sequence of *O. longistaminata* shows a *Ptr* allele encoding a protein sequence more similar to *Ptr*_*b*_ than any of the other variants we observed, however, two AA polymorphisms are present in the C terminal region relative to *Ptr*_*b*_ (Fig. 3, Supplementary Fig. 4, Supplementary Data 4). Given that the mapped region containing *Pi57(t)* contains just 6 genes^54^, there is a reasonable likelihood that a functional allele of *Ptr* is responsible for the *Pi57(t)* locus derived from *O. longistaminata*.

Unlike *Ptr*_*b*_ and *Ptr*_*c*_, the two additional functional alleles of the *Pia* resistance genes *RGA4* and *RGA5* we identified, did not show differential resistance responses to *M. oryzae* isolates expressing either *AVR-Pia* or *AVR1-CO39*. This is despite these *Pia* alleles encoding for AA changes in the HMA effector binding domain of RGA5 (Supplementary Figs. 8, 9), or in the case of one genotype (IRIS_313-12190), 66 AA changes in the helper NLR RGA4 (Supplementary Fig. 7). Interestingly, several non-associated accessions that do not carry *RGA5* had similar intact alleles of *RGA4* encoding only 8 AA changes relative to the newly identified functional RGA4 (IRIS_313-12190) (Supplementary Fig. 7). This diversity in RGA4 is interesting, given that RGA4 is an auto-active NLR, triggering resistance responses in the absence of RGA5^24^. All intact variants of RGA4 we identified feature the critical auto-active variants of the MHD motif (TYG or MYG) required for RGA4 mediated resistance responses^24^ (Supplementary Fig. 7). A recently cloned pair of NLRs underpinning the *Pias* resistance locus are syntenic to *RGA4* and *RGA5*, with *Pias-1* sharing homology to the divergent form of *RGA4* we identified in IRIS_313-12190 (Supplementary Fig. 7), and *Pias-2* sharing homology to the Nipponbare gene *LOC_Os11g11810*^55^ (Supplementary Fig. 12). Based on transient expression assays, RGA4 and Pias-1 induced cell-death is suppressed by RGA5, except when AVR-Pia is also present^55^. This result supports our observation that RGA5 can function in the presence of divergent forms of RGA4/Pias-1, as was the case for IRIS_313-12190 (Fig. 4, Supplementary Fig. 7). When querying available genomic sequences, we identified 12 genomes which contain homologs of *Pias-2/LOC_Os11g11810* rather than *RGA5* (Supplementary Fig. 12, Supplementary Data 4). Four of the genomes contained *Pias-2-like* (>99.80% sequence identity) alleles which encode one to three AA changes relative to Pias-2, while all remaining genomes encode LOC_Os11g11810-like variants that do not contain the 91 C-terminal AA of Pias-2 and instead carry a shorter C-terminus (Supplementary Fig. 12). All of the *Pias-2-like* alleles encode the same C-terminal AA change H1287Y when compared to Pias-2 (Supplementary Fig. 12).

The N-terminal portion of the coiled-coil domains of RGA4_1–130_ and RGA5_1–133_ were shown to interact in Y2H assays, but the CC domain of RGA5_1–173_ alone was insufficient to suppress RGA4 auto-activity^24^. With the exception of 9 AA changes, protein homology for the N-terminal 148 AA is maintained between RGA5 and Pias-2*/*LOC_Os11g11810. Besides these 148 AA, Pias-2/LOC_Os11g11810 is otherwise divergent from RGA5. Given that Pias-2 is able to suppress RGA4 auto-activity, the divergent C-terminus encoded by *LOC_Os11g11810-like* alleles could represent an additional receptor component that releases RGA4/Pias-1 to trigger a resistance response similar to the *Pia* and *Pias* NLR pairs.

This study provides a robust conceptual approach for rapid gene cloning and assessment of genetic diversity, which has led to new biological insights into rice blast resistance. These results add to improving knowledge-guided disease resistance breeding strategies, for example through the deployment of optimal disease resistance gene stacks.

## Materials and Methods

### Selection of rice blast diversity panel and infection testing of rice accessions

Accessions from the 3000 rice genomes project were selected based on the absence of some known rice blast resistance genes. This was executed by using gene specific markers and bioinformatic analyses to screen for genotypes containing *Pi2/9*, *Pik,* and *Pi3/5*. Accessions were further selected based on their country of origin and genotype subgrouping, resulting in a set of 500 genetically diverse accessions for infection testing (Supplementary Data 1). Infection testing was performed using a set of diverse *M. oryzae* isolates from the Philippines which was shown to exhibit varying virulence spectra when infecting near-isogenic rice lines containing different rice blast resistance loci (Supplementary Table 3). For each *M. oryzae* isolate infection, three replicates of 10 plants from each of the 500 rice accessions were grown for 14 days and then spray-inoculated with *M. oryzae* spore solutions (1–2 × 10^6^ spores/ml). Inoculated plantlets were covered and incubated in cooled conditions (max 28°C) overnight, then transferred and kept in a misted greenhouse for 6 days. Disease assessment was performed 7 days post inoculation. Lesion type for each plant (maximum 10 plants per accession, per isolate infection) was recorded based on the evaluation system published by JIRCAS^38^. A summary of infection testing data is included in Supplementary Data 1.

### Genome-wide-association-studies and investigating candidate gene regions

GWAS was performed using the “3K RG 1M GWAS SNP Dataset, all chromosomes” from the Rice SNP-seek database (https://snp-seek.irri.org/_download.zul;jsessionid=CF24BFBA8A7CEEAD273D6EAC4467EC98). BED/FAM/BIM files were converted to TFAM/TPED files using PLINK^56^. GWAS were performed using the R package GenABEL^57^. To correct for population structure, a linear mixed (polygenic) model was estimated using the polygenic function^58^ and association was determined using the mmscore function^59^. Both binomial and Gaussian treatments of infection data were used as inputs for GWAS. Infection data was cleaned prior to analysis by removing accessions with a spread of infection scores across 5 or 6 units on the 0–5 infection scale, or with a SD > 1.3. Using the same SNP set as used for GWAS, candidate gene regions surrounding peak associated SNPs were defined using the Solid Spine of LD function in Haploview^60^. SNPs in associated and non-associated accessions were extracted using the Rice SNP-seek database^61^. The SNP variants for each accession, at each peak associated SNP, were tabulated alongside resistance scores to determine which SNP variants were commonly associated with rice blast resistance to a particular *M. oryzae* isolate.

### Genome sequencing and de novo assembly

High molecular weight DNA extraction from 2-week-old rice leaves was performed^62^. Genomic DNA was confirmed to be larger than 100 kb on average using an Agilent Femto Pulse System. DNA integrity was confirmed by agarose gel electrophoresis and concentration was determined using a Life Technologies Qubit Fluorometer. Genome sequencing was performed using linked read sequencing^63^. Library preparation was performed using a 10X genomics, Chromium™ Genome Library, Gel Bead and Multiplex Kit according to manufacturer’s instructions. 150 bp paired-end sequencing was obtained using an Illumina *HiSeq* X Ten system. Genome assembly was performed using the 10X Genomics Supernova - 2.1.1 software package for d*e novo* assembly^64^. The de novo assembled genomes were queried by BLAST search to identify scaffolds containing strong homology to candidate resistance genes. The rice accessions carrying the Mo15-23 and Mo15-24 resistance association on chromosome 12 all carried varying sizes of missing sequence 2 kb upstream of the *Ptr* start codon. This region was separated by an N string during scaffold assembly due to linked reads being assigned to the same scaffold, but short reads not being able to be assembled across the sequence gap. Primers (Supplementary Data 6) were designed to span this region and resolve the complete genomic sequence for the accession IRIS_313-10314 revealing a duplicate repeat of 108 bp.

### *Ptr*_*b*_ rice transformation and infection testing

The genomic sequence of *Ptr*_*b*_ was amplified from IRIS_313-10314 genomic DNA in two fragments with primers at the distal 5’ and 3’ ends of the *Ptr*_*b*_ genomic region containing a 15 bp overlap to linearized (digested with *HindIII* and *BsrGI*) pIPKB001 binary vector^65^. Internal primers were positioned such that an 18 bp overlap between the two genomic fragments would be generated (Supplementary Data 6). Genomic fragments were fused with the linearized pIPKB001 binary vector by In-Fusion cloning (Takara Bio Inc). Correct assembly of the intact *Ptr* sequence in pIPKB001 was confirmed by Sanger sequencing. Transformation of CO39 and isolation of homozygous single copy transformants and sibling lines was performed^66^. Four-week-old plants were spray-infected with a solution containing 40,000 *M. oryzae* spores/ml in water containing 0.1% Tween®20. Following infection, plants were placed in trays containing water, covered with a wet plastic hood, and left in the dark for 16 h. Hoods were removed from the plants and they were left to dry before transferring into 12-h photoperiod, 28 °C day and 23 °C night temperature. Symptoms were analyzed seven days after inoculation, on the youngest fully expanded leaf at the time of inoculation. Representative infection scoring leaf images and associated classifications can be found in Supplementary Fig. 13.

### Structural modeling of *Pia* HMA domain variants

The RGA5 HMA domain variants were modeled on the SWISS-MODEL web site with the HMA domain of the Sasanishiki RGA5 complexed with AVR1-CO39 (PDB: 5ZNG) as a template. For IRIS_313-11786, the ASVNGVESMQ-loop sequence was modeled by RCD+: Fast loop modeling server^67^. The loop with the least clashes with AVR1-CO39 in the complex was selected. This HMA AVR1-CO39 complex was further refined on the GalaxyWEB server^68^ and the complex model with the lowest energy was selected.

### Infection testing of *Pia* variants using Guy11 transformants

To produce spores, *M. oryzae* strains were grown for 5 days at 25 °C on rice flour agar (Ref. ^69^). Infection assays were performed by spraying 3-week-old rice plants with a suspension of *M. oryzae* conidiospores adjusted to 35,000 spores/ml in water containing 0.5% of gelatin, incubating sprayed plants at 100% humidity in the dark at 23 °C for 16 h, then transferring them into a growth chamber with 12-h photoperiod, 28 °C day and 23 °C night temperature^69^. Symptoms were analyzed 7 days after inoculation on the youngest leaf fully expanded at the time of inoculation. Representative infection scoring leaf images and associated classifications can be found in Supplementary Fig. 13.

### Statistics and reproducibility

Replicates (n) are defined as individual plants or a single sample from individual plants. For infection studies, a single inoculated leaf of the same age from each plant is assessed and this constitutes a biological replicate. Sample sizes and statistical tests are indicated in figure legends.

### Supplementary information

Supplementary Information

Description of Additional Supplementary Materials

Supplementary Data 1

Supplementary Data 2

Supplementary Data 3

Supplementary Data 4

Supplementary Data 5

Supplementary Data 6

Supplementary Data 7

Supplementary Data 8

## Data Availability

Source data can be found in Supplementary Data 1. Sequence information generated in this study can be accessed using the links below. Previously published sequence information can be accessed via NCBI using identifiers listed below. All extracted protein sequences can be found in Supplementary Data 7. Protein MSA alignments were presented using ESPript^70^. https://espript.ibcp.fr. Plant material is available under material transfer agreement from IRRI (diversity panel), or from authors on request (transgenic material). 10 Genome assemblies generated in this study - https://www.ncbi.nlm.nih.gov/bioproject/822270/ : IRIS_313-10059 - https://www.ncbi.nlm.nih.gov/assembly/GCA_936157995.1. IRIS_313-12190 - https://www.ncbi.nlm.nih.gov/assembly/GCA_936150925.1. IRIS_313-10879 - https://www.ncbi.nlm.nih.gov/assembly/GCA_936157035.1. IRIS_313-11786 - https://www.ncbi.nlm.nih.gov/assembly/GCA_936158435.1. IRIS_313-11127 - https://www.ncbi.nlm.nih.gov/assembly/GCA_936153055.1. IRIS_313-7914 - https://www.ncbi.nlm.nih.gov/assembly/GCA_936157175.1. IRIS_313-10314 - https://www.ncbi.nlm.nih.gov/assembly/GCA_936154975.1. IRIS_313-11360 - https://www.ncbi.nlm.nih.gov/assembly/GCA_936145055.1. IRIS_313-10738 - https://www.ncbi.nlm.nih.gov/assembly/GCA_936157615.1. IRIS_313-8554 - https://www.ncbi.nlm.nih.gov/assembly/GCA_936157125.1. Published sequence information used in this study. CO39: GCA_001611235.1^71^ (Genome assembly), SWLY01018120.1 (Ptr). *Oryza longistaminata*: GCA_000789195.1. *Oryza sativa* cultivar Katy disease resistance protein (Ptr) mRNA: MG385187.1^8^. *Oryza sativa* cultivar PINo.1 disease resistance protein (Ptr) mRNA: MG385188.1^8^. *Oryza sativa* cultivar BHA disease resistance protein (Ptr) mRNA: MG385192.1^8^. *Oryza sativa* cultivar PI4 disease resistance protein (Ptr) mRNA: MG385189.1^8^. *Oryza sativa* cultivar YT16 disease resistance protein (Ptr) mRNA: MG385190.1^8^. *Oryza sativa* cultivar Amane disease resistance protein (Ptr) mRNA: MG385191.1^8^. 93-11: GenBank sequence - Chromosome 11: CM012063.1^41^, GenBank sequence - Chromosome 12: CM012064.1^41^, AB604628 (RGA5)^22^. Sasanishiki: AB604627 (RGA5)^22^. *Pias-1*: LC672059; *Pias-2*: LC672060^55^.

## Code availability

An example R script for performing GWAS and the complete compatible GWAS dataset can be found in Supplementary Data 8.
