# Supplementary material for: Genome-wide association analysis uncovers rice blast resistance alleles of Ptr and Pia
Source: Commun Biol. 2024 May 20;7:607. doi: 10.1038/s42003-024-06244-z (PMC11106262; doi:10.1038/s42003-024-06244-z)
Supplement: Supplementary file 1 — Supplementary Information [file 42003_2024_6244_MOESM1_ESM.pdf]

Supplementary information

Supplementary Figures pages 2-28

Supplementary Tables pages 29-35

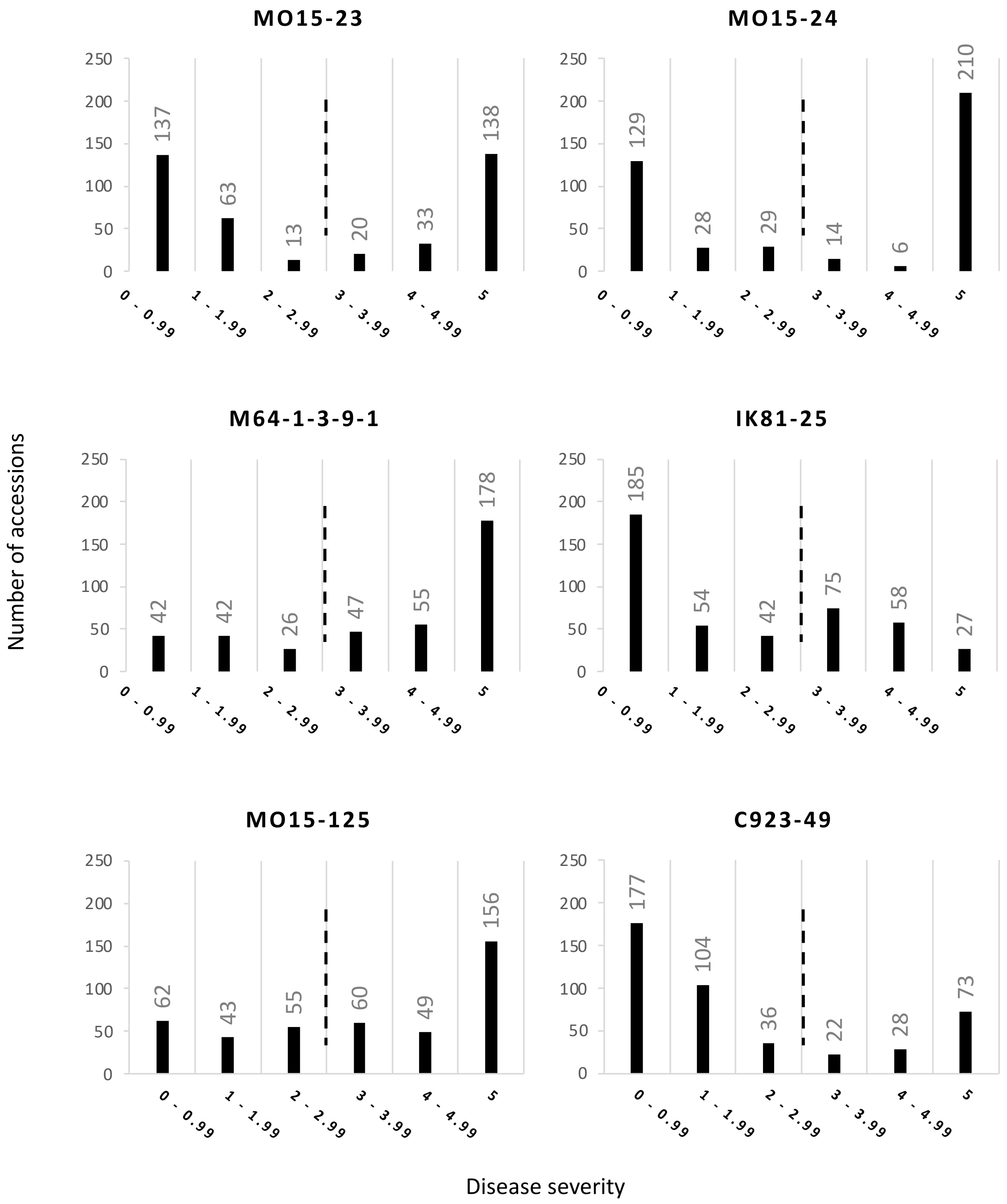

Supplementary Figure 1. Frequency distribution of rice blast disease severity scores. Distribution of *M. oryzae* inoculation scores shown for each of the six pathogen isolates. Rice accessions with a standard deviation greater than 1.3 between across replicates were excluded. 0 = no symptoms; 5 = eyespot lesions greater than 2 mm. The average rice blast disease severity score for each accession infected with six different *M. oryzae* isolates is presented as the frequency of occurrence in the bin ranges indicated on the X-axis (disease severity). Vertical dashed lines indicate the cutoff for resistant and susceptibility. For all isolates, except Mo15-125, vertical dashed lines also define the bimodal distribution of infection scores.

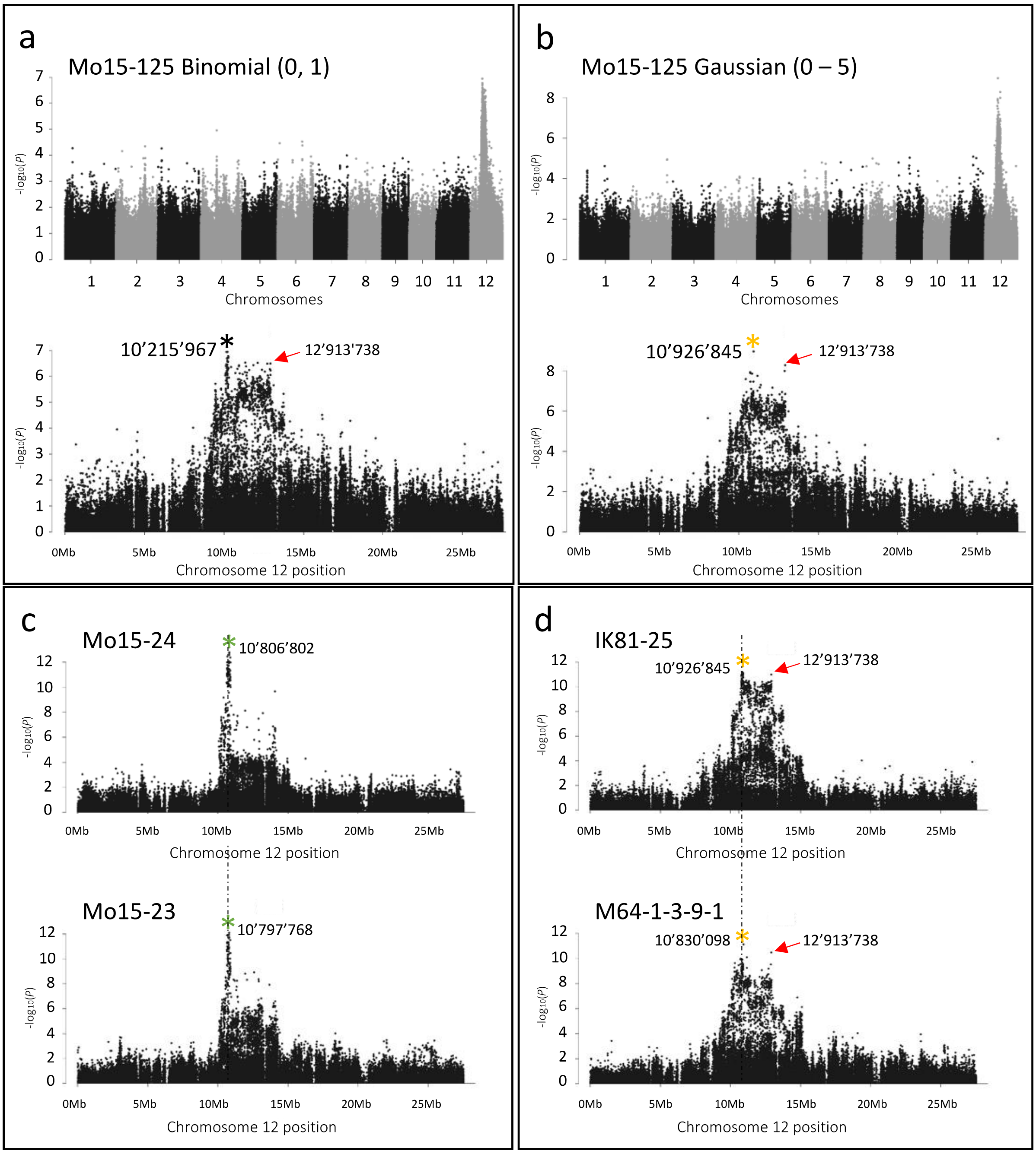

Supplementary Figure 2. Additional chromosome 12 association peaks produced by different *M. oryzae* isolates and treatments of input data. a, b GWAS performed on the Mo15-125 infection data set produces different peak associations when infection scores were treated as binomial (resistant vs susceptible) or gaussian (0-5 disease severity scores), and used as input for GWAS. **Every other infection data set produced the same peak associations regardless of input data type.** Peak SNPs are indicated by an asterisk symbol and the nucleotide position (based on Nipponbare reference assembly) is indicated. Red arrows indicate the position of a second peak SNP. The Mo15-125 Gaussian GWAS peak SNP is identical to the M64-1-3-9-1 GWAS peak SNP at position 10,926,845 (shown in panel D). **c, d** Manhattan plots of chromosome 12 associations are shown for Mo15-23/24 (group 2), as well as for IK81-25 and M64-1-3-9-1 (group 1), based on binomial input data. Peak SNPs are marked with an asterisk and the nucleotide position is indicated. **d** Red arrows indicate the position of a second peak SNP that co-associates with the peak SNPs of IK81-25 and M64-1-3-9-1 at 10.92 and 10.83 Mb. All nucleotide positions are relative to the Nipponbare reference genome. The asterisks denoting chromosome 12 peak SNPs are color coded to indicate that particular subsets of accessions carrying the associated variant of the chromosome 12 peak SNPs are shared for Mo15-24 and 23 (green – group 2), and for Mo15-125, IK81-25 and M64-1-3-9-1 (yellow – group 1).

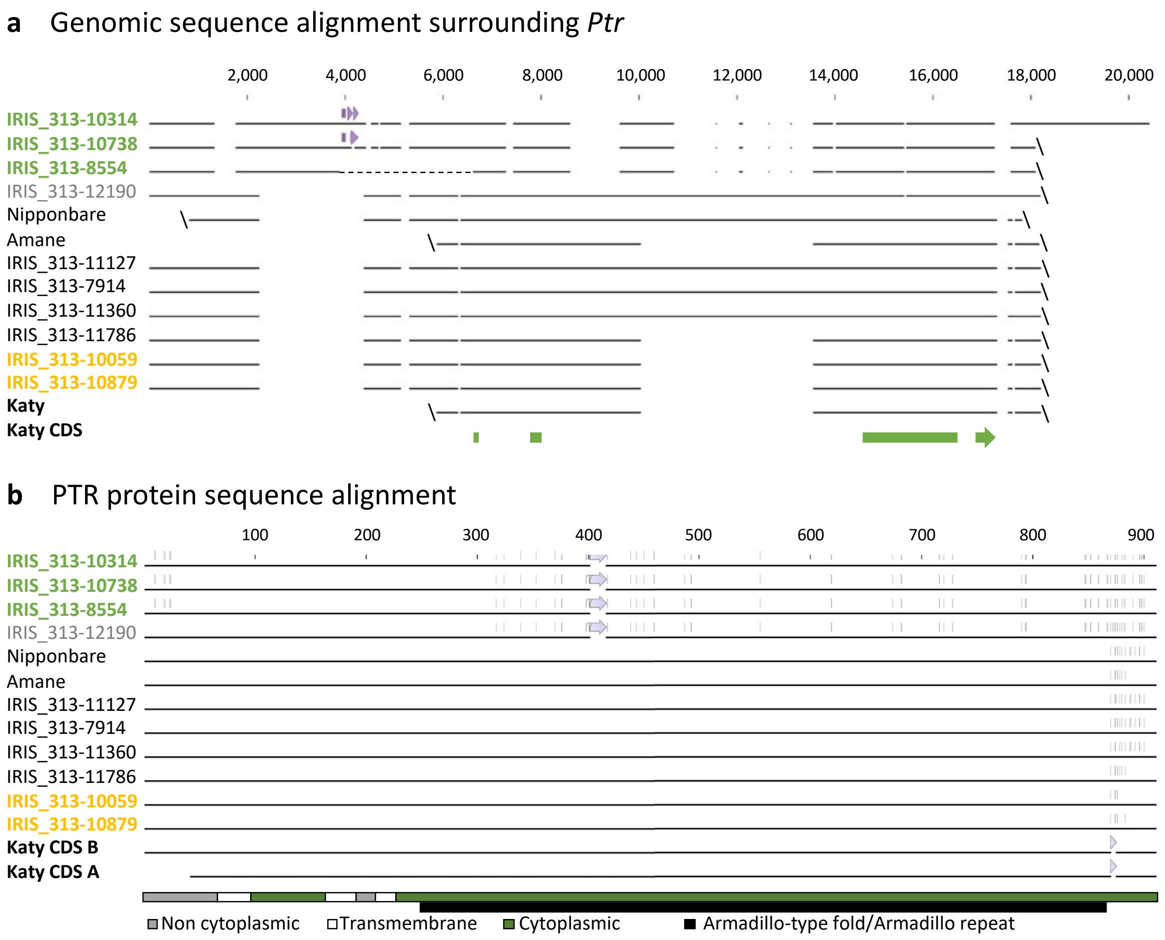

Supplementary Figure 3. Schematic representation of *Ptr* genomic and protein multiple sequence alignments. a Genomic sequence surrounding *Ptr* and, **b** Ptr protein sequence alignment shown for associated and non-associated de-novo assembled accessions, and for Nipponbare, Amane (identical to CO39 *Ptr*) and Katy (previously identified *Ptr* allele). Mo15-23 and Mo15-24 resistance associated accession names have green labels, Ik81-25, M64-1-3-9-1 and Mo15-125 resistance associated accessions have yellow labels, and IRIS-313-12190 which carried resistance associations to Mo15-23, Mo15-24, Ik81-25, M64-1-3-9-1 and Mo15-125 at chromosome 12 is shown in grey. **a** Purple arrows above genomic sequence indicate the position of tandem repeats detected using Tandem repeats finder (Benson, 1999). A dashed line on IRIS_313-8554 genomic sequence indicates a gap in assembled sequence due to tandem repeat. PCR amplification using primers flanking the ambiguous region allowed for the sequence to be resolved for the IRIS-313-10314 accession. All sequences are trimmed for alignment to include 3 kb of sequence upstream of *Ptr* start site (except for IRIS-313-10314 where 5 kb of sequence upstream of *Ptr* start codon is included) and 3 kb downstream of *Ptr* stop codon. Trimmed sequence ends are denoted by a backslash (\). All other gaps in genomic sequence represent the position of in-dels. Genomic sequence for the *Ptr* locus from the accessions Katy and Amane represents the maximum available sequence reported (Zhao *et al.*, 2018). **b** A schematic of the protein multiple sequence alignment of the largest isoform of Ptr is shown with AA changes and in-dels relative to the Katy allele shown in pale purple. The four amino acid deletion found in the Katy allele is shown as a pale purple triangle above the Katy isoforms. Predicted domain structure of Ptr is shown below the protein alignment schematic. Genomic sequence alignments are provided as a supplementary file. Protein sequence alignments are included in subsequent supplementary figures.

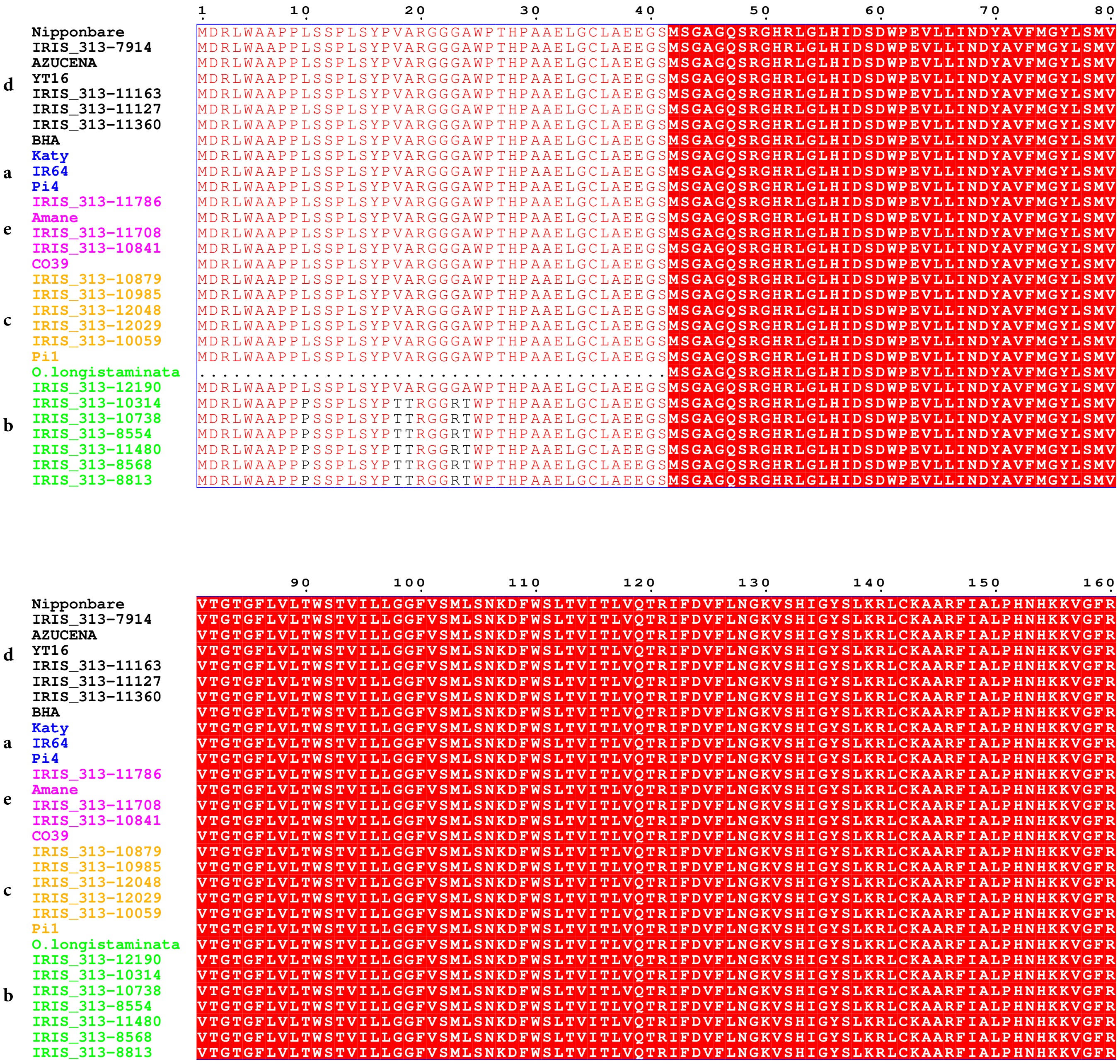

1 10 20 30 40 50 60 70 80
Nipponbare MDRLWAAPPLSSPLSYPVARGGGAWPTHPAAELGCLAEEGSMSGAGQSRGHRLGLHIDSDWPEVLLINDYAVFMGYLSMV
IRIS_313-7914 MDRLWAAPPLSSPLSYPVARGGGAWPTHPAAELGCLAEEGSMSGAGQSRGHRLGLHIDSDWPEVLLINDYAVFMGYLSMV
AZUCENA MDRLWAAPPLSSPLSYPVARGGGAWPTHPAAELGCLAEEGSMSGAGQSRGHRLGLHIDSDWPEVLLINDYAVFMGYLSMV
d
YT16 MDRLWAAPPLSSPLSYPVARGGGAWPTHPAAELGCLAEEGSMSGAGQSRGHRLGLHIDSDWPEVLLINDYAVFMGYLSMV
IRIS_313-11163 MDRLWAAPPLSSPLSYPVARGGGAWPTHPAAELGCLAEEGSMSGAGQSRGHRLGLHIDSDWPEVLLINDYAVFMGYLSMV
IRIS_313-11127 MDRLWAAPPLSSPLSYPVARGGGAWPTHPAAELGCLAEEGSMSGAGQSRGHRLGLHIDSDWPEVLLINDYAVFMGYLSMV
IRIS_313-11360 MDRLWAAPPLSSPLSYPVARGGGAWPTHPAAELGCLAEEGSMSGAGQSRGHRLGLHIDSDWPEVLLINDYAVFMGYLSMV
BHA MDRLWAAPPLSSPLSYPVARGGGAWPTHPAAELGCLAEEGSMSGAGQSRGHRLGLHIDSDWPEVLLINDYAVFMGYLSMV
Katy MDRLWAAPPLSSPLSYPVARGGGAWPTHPAAELGCLAEEGSMSGAGQSRGHRLGLHIDSDWPEVLLINDYAVFMGYLSMV
a
IR64 MDRLWAAPPLSSPLSYPVARGGGAWPTHPAAELGCLAEEGSMSGAGQSRGHRLGLHIDSDWPEVLLINDYAVFMGYLSMV
Pi4 MDRLWAAPPLSSPLSYPVARGGGAWPTHPAAELGCLAEEGSMSGAGQSRGHRLGLHIDSDWPEVLLINDYAVFMGYLSMV
IRIS_313-11786 MDRLWAAPPLSSPLSYPVARGGGAWPTHPAAELGCLAEEGSMSGAGQSRGHRLGLHIDSDWPEVLLINDYAVFMGYLSMV
Amane MDRLWAAPPLSSPLSYPVARGGGAWPTHPAAELGCLAEEGSMSGAGQSRGHRLGLHIDSDWPEVLLINDYAVFMGYLSMV
e
IRIS_313-11708 MDRLWAAPPLSSPLSYPVARGGGAWPTHPAAELGCLAEEGSMSGAGQSRGHRLGLHIDSDWPEVLLINDYAVFMGYLSMV
IRIS_313-10841 MDRLWAAPPLSSPLSYPVARGGGAWPTHPAAELGCLAEEGSMSGAGQSRGHRLGLHIDSDWPEVLLINDYAVFMGYLSMV
CO39 MDRLWAAPPLSSPLSYPVARGGGAWPTHPAAELGCLAEEGSMSGAGQSRGHRLGLHIDSDWPEVLLINDYAVFMGYLSMV
IRIS_313-10879 MDRLWAAPPLSSPLSYPVARGGGAWPTHPAAELGCLAEEGSMSGAGQSRGHRLGLHIDSDWPEVLLINDYAVFMGYLSMV
IRIS_313-10985 MDRLWAAPPLSSPLSYPVARGGGAWPTHPAAELGCLAEEGSMSGAGQSRGHRLGLHIDSDWPEVLLINDYAVFMGYLSMV
c
IRIS_313-12048 MDRLWAAPPLSSPLSYPVARGGGAWPTHPAAELGCLAEEGSMSGAGQSRGHRLGLHIDSDWPEVLLINDYAVFMGYLSMV
IRIS_313-12029 MDRLWAAPPLSSPLSYPVARGGGAWPTHPAAELGCLAEEGSMSGAGQSRGHRLGLHIDSDWPEVLLINDYAVFMGYLSMV
IRIS_313-10059 MDRLWAAPPLSSPLSYPVARGGGAWPTHPAAELGCLAEEGSMSGAGQSRGHRLGLHIDSDWPEVLLINDYAVFMGYLSMV
Pi1 MDRLWAAPPLSSPLSYPVARGGGAWPTHPAAELGCLAEEGSMSGAGQSRGHRLGLHIDSDWPEVLLINDYAVFMGYLSMV
O.longistaminata ....MSGAGQSRGHRLGLHIDSDWPEVLLINDYAVFMGYLSMV
IRIS_313-12190 MDRLWAAPPLSSPLSYPVARGGGAWPTHPAAELGCLAEEGSMSGAGQSRGHRLGLHIDSDWPEVLLINDYAVFMGYLSMV
IRIS_313-10314 MDRLWAAPPPSSPLSYPTTRGGRTWPTHPAAELGCLAEEGSMSGAGQSRGHRLGLHIDSDWPEVLLINDYAVFMGYLSMV
b
IRIS_313-10738 MDRLWAAPPPSSPLSYPTTRGGRTWPTHPAAELGCLAEEGSMSGAGQSRGHRLGLHIDSDWPEVLLINDYAVFMGYLSMV
IRIS_313-8554 MDRLWAAPPPSSPLSYPTTRGGRTWPTHPAAELGCLAEEGSMSGAGQSRGHRLGLHIDSDWPEVLLINDYAVFMGYLSMV
IRIS_313-11480 MDRLWAAPPPSSPLSYPTTRGGRTWPTHPAAELGCLAEEGSMSGAGQSRGHRLGLHIDSDWPEVLLINDYAVFMGYLSMV
IRIS_313-8568 MDRLWAAPPPSSPLSYPTTRGGRTWPTHPAAELGCLAEEGSMSGAGQSRGHRLGLHIDSDWPEVLLINDYAVFMGYLSMV
IRIS_313-8813 MDRLWAAPPPSSPLSYPTTRGGRTWPTHPAAELGCLAEEGSMSGAGQSRGHRLGLHIDSDWPEVLLINDYAVFMGYLSMV
90 100 110 120 130 140 150 160
Nipponbare VTGTGFLVLTWSTVILLGGFVSMLSNKDFWSLTVITLVQTRIFDVFLNGKVSHIGYSLKRLCKAARFIALPHNHKKVGFR
IRIS_313-7914 VTGTGFLVLTWSTVILLGGFVSMLSNKDFWSLTVITLVQTRIFDVFLNGKVSHIGYSLKRLCKAARFIALPHNHKKVGFR
AZUCENA VTGTGFLVLTWSTVILLGGFVSMLSNKDFWSLTVITLVQTRIFDVFLNGKVSHIGYSLKRLCKAARFIALPHNHKKVGFR
d
YT16 VTGTGFLVLTWSTVILLGGFVSMLSNKDFWSLTVITLVQTRIFDVFLNGKVSHIGYSLKRLCKAARFIALPHNHKKVGFR
IRIS_313-11163 VTGTGFLVLTWSTVILLGGFVSMLSNKDFWSLTVITLVQTRIFDVFLNGKVSHIGYSLKRLCKAARFIALPHNHKKVGFR
IRIS_313-11127 VTGTGFLVLTWSTVILLGGFVSMLSNKDFWSLTVITLVQTRIFDVFLNGKVSHIGYSLKRLCKAARFIALPHNHKKVGFR
IRIS_313-11360 VTGTGFLVLTWSTVILLGGFVSMLSNKDFWSLTVITLVQTRIFDVFLNGKVSHIGYSLKRLCKAARFIALPHNHKKVGFR
BHA VTGTGFLVLTWSTVILLGGFVSMLSNKDFWSLTVITLVQTRIFDVFLNGKVSHIGYSLKRLCKAARFIALPHNHKKVGFR
Katy VTGTGFLVLTWSTVILLGGFVSMLSNKDFWSLTVITLVQTRIFDVFLNGKVSHIGYSLKRLCKAARFIALPHNHKKVGFR
a
IR64 VTGTGFLVLTWSTVILLGGFVSMLSNKDFWSLTVITLVQTRIFDVFLNGKVSHIGYSLKRLCKAARFIALPHNHKKVGFR
Pi4 VTGTGFLVLTWSTVILLGGFVSMLSNKDFWSLTVITLVQTRIFDVFLNGKVSHIGYSLKRLCKAARFIALPHNHKKVGFR
IRIS_313-11786 VTGTGFLVLTWSTVILLGGFVSMLSNKDFWSLTVITLVQTRIFDVFLNGKVSHIGYSLKRLCKAARFIALPHNHKKVGFR
Amane VTGTGFLVLTWSTVILLGGFVSMLSNKDFWSLTVITLVQTRIFDVFLNGKVSHIGYSLKRLCKAARFIALPHNHKKVGFR
e
IRIS_313-11708 VTGTGFLVLTWSTVILLGGFVSMLSNKDFWSLTVITLVQTRIFDVFLNGKVSHIGYSLKRLCKAARFIALPHNHKKVGFR
IRIS_313-10841 VTGTGFLVLTWSTVILLGGFVSMLSNKDFWSLTVITLVQTRIFDVFLNGKVSHIGYSLKRLCKAARFIALPHNHKKVGFR
CO39 VTGTGFLVLTWSTVILLGGFVSMLSNKDFWSLTVITLVQTRIFDVFLNGKVSHIGYSLKRLCKAARFIALPHNHKKVGFR
IRIS_313-10879 VTGTGFLVLTWSTVILLGGFVSMLSNKDFWSLTVITLVQTRIFDVFLNGKVSHIGYSLKRLCKAARFIALPHNHKKVGFR
IRIS_313-10985 VTGTGFLVLTWSTVILLGGFVSMLSNKDFWSLTVITLVQTRIFDVFLNGKVSHIGYSLKRLCKAARFIALPHNHKKVGFR
c
IRIS_313-12048 VTGTGFLVLTWSTVILLGGFVSMLSNKDFWSLTVITLVQTRIFDVFLNGKVSHIGYSLKRLCKAARFIALPHNHKKVGFR
IRIS_313-12029 VTGTGFLVLTWSTVILLGGFVSMLSNKDFWSLTVITLVQTRIFDVFLNGKVSHIGYSLKRLCKAARFIALPHNHKKVGFR
IRIS_313-10059 VTGTGFLVLTWSTVILLGGFVSMLSNKDFWSLTVITLVQTRIFDVFLNGKVSHIGYSLKRLCKAARFIALPHNHKKVGFR
Pi1 VTGTGFLVLTWSTVILLGGFVSMLSNKDFWSLTVITLVQTRIFDVFLNGKVSHIGYSLKRLCKAARFIALPHNHKKVGFR
O.longistaminata VTGTGFLVLTWSTVILLGGFVSMLSNKDFWSLTVITLVQTRIFDVFLNGKVSHIGYSLKRLCKAARFIALPHNHKKVGFR
IRIS_313-12190 VTGTGFLVLTWSTVILLGGFVSMLSNKDFWSLTVITLVQTRIFDVFLNGKVSHIGYSLKRLCKAARFIALPHNHKKVGFR
IRIS_313-10314 VTGTGFLVLTWSTVILLGGFVSMLSNKDFWSLTVITLVQTRIFDVFLNGKVSHIGYSLKRLCKAARFIALPHNHKKVGFR
b
IRIS_313-10738 VTGTGFLVLTWSTVILLGGFVSMLSNKDFWSLTVITLVQTRIFDVFLNGKVSHIGYSLKRLCKAARFIALPHNHKKVGFR
IRIS_313-8554 VTGTGFLVLTWSTVILLGGFVSMLSNKDFWSLTVITLVQTRIFDVFLNGKVSHIGYSLKRLCKAARFIALPHNHKKVGFR
IRIS_313-11480 VTGTGFLVLTWSTVILLGGFVSMLSNKDFWSLTVITLVQTRIFDVFLNGKVSHIGYSLKRLCKAARFIALPHNHKKVGFR
IRIS_313-8568 VTGTGFLVLTWSTVILLGGFVSMLSNKDFWSLTVITLVQTRIFDVFLNGKVSHIGYSLKRLCKAARFIALPHNHKKVGFR
IRIS_313-8813 VTGTGFLVLTWSTVILLGGFVSMLSNKDFWSLTVITLVQTRIFDVFLNGKVSHIGYSLKRLCKAARFIALPHNHKKVGFR

```
                            170       180       190       200       210       220       230       240
                              .         .         .         .         .         .         .         .
  Nipponbare         GAVRVLVFTIVLCPLFLLYMFGLFVSPWISLWRLIQQDYGVTAGDSSSKAHLQPALVVLYSLALFQGVLFYYRAISAWEE
  IRIS_313-7914      GAVRVLVFTIVLCPLFLLYMFGLFVSPWISLWRLIQQDYGVTAGDSSSKAHLQPALVVLYSLALFQGVLFYYRAISAWEE
  AZUCENA            GAVRVLVFTIVLCPLFLLYMFGLFVSPWISLWRLIQQDYGVTAGDSSSKAHLQPALVVLYSLALFQGVLFYYRAISAWEE
d YT16               GAVRVLVFTIVLCPLFLLYMFGLFVSPWISLWRLIQQDYGVTAGDSSSKAHLQPALVVLYSLALFQGVLFYYRAISAWEE
  IRIS_313-11163     GAVRVLVFTIVLCPLFLLYMFGLFVSPWISLWRLIQQDYGVTAGDSSSKAHLQPALVVLYSLALFQGVLFYYRAISAWEE
  IRIS_313-11127     GAVRVLVFTIVLCPLFLLYMFGLFVSPWISLWRLIQQDYGVTAGDSSSKAHLQPALVVLYSLALFQGVLFYYRAISAWEE
  IRIS_313-11360     GAVRVLVFTIVLCPLFLLYMFGLFVSPWISLWRLIQQDYGVTAGDSSSKAHLQPALVVLYSLALFQGVLFYYRAISAWEE
  BHA                GAVRVLVFTIVLCPLFLLYMFGLFVSPWISLWRLIQQDYGVTAGDSSSKAHLQPALVVLYSLALFQGVLFYYRAISAWEE
  Katy               GAVRVLVFTIVLCPLFLLYMFGLFVSPWISLWRLIQQDYGVTAGDSSSKAHLQPALVVLYSLALFQGVLFYYRAISAWEE
a IR64               GAVRVLVFTIVLCPLFLLYMFGLFVSPWISLWRLIQQDYGVTAGDSSSKAHLQPALVVLYSLALFQGVLFYYRAISAWEE
  Pi4                GAVRVLVFTIVLCPLFLLYMFGLFVSPWISLWRLIQQDYGVTAGDSSSKAHLQPALVVLYSLALFQGVLFYYRAISAWEE
  IRIS_313-11786     GAVRVLVFTIVLCPLFLLYMFGLFVSPWISLWRLIQQDYGVTAGDSSSKAHLQPALVVLYSLALFQGVLFYYRAISAWEE
  Amane              GAVRVLVFTIVLCPLFLLYMFGLFVSPWISLWRLIQQDYGVTAGDSSSKAHLQPALVVLYSLALFQGVLFYYRAISAWEE
e IRIS_313-11708     GAVRVLVFTIVLCPLFLLYMFGLFVSPWISLWRLIQQDYGVTAGDSSSKAHLQPALVVLYSLALFQGVLFYYRAISAWEE
  IRIS_313-10841     GAVRVLVFTIVLCPLFLLYMFGLFVSPWISLWRLIQQDYGVTAGDSSSKAHLQPALVVLYSLALFQGVLFYYRAISAWEE
  CO39               GAVRVLVFTIVLCPLFLLYMFGLFVSPWISLWRLIQQDYGVTAGDSSSKAHLQPALVVLYSLALFQGVLFYYRAISAWEE
  IRIS_313-10879     GAVRVLVFTIVLCPLFLLYMFGLFVSPWISLWRLIQQDYGVTAGDSSSKAHLQPALVVLYSLALFQGVLFYYRAISAWEE
  IRIS_313-10985     GAVRVLVFTIVLCPLFLLYMFGLFVSPWISLWRLIQQDYGVTAGDSSSKAHLQPALVVLYSLALFQGVLFYYRAISAWEE
  IRIS_313-12048     GAVRVLVFTIVLCPLFLLYMFGLFVSPWISLWRLIQQDYGVTAGDSSSKAHLQPALVVLYSLALFQGVLFYYRAISAWEE
c IRIS_313-12029     GAVRVLVFTIVLCPLFLLYMFGLFVSPWISLWRLIQQDYGVTAGDSSSKAHLQPALVVLYSLALFQGVLFYYRAISAWEE
  IRIS_313-10059     GAVRVLVFTIVLCPLFLLYMFGLFVSPWISLWRLIQQDYGVTAGDSSSKAHLQPALVVLYSLALFQGVLFYYRAISAWEE
  Pi1                GAVRVLVFTIVLCPLFLLYMFGLFVSPWISLWRLIQQDYGVTAGDSSSKAHLQPALVVLYSLALFQGVLFYYRAISAWEE
  O.longistaminata   GAVRVLVFTIVLCPLFLLYMFGLFVSPWISLWRLIQQDYGVTAGDSSSKAHLQPALVVLYSLALFQGVLFYYRAISAWEE
  IRIS_313-12190     GAVRVLVFTIVLCPLFLLYMFGLFVSPWISLWRLIQQDYGVTAGDSSSKAHLQPALVVLYSLALFQGVLFYYRAISAWEE
  IRIS_313-10314     GAVRVLVFTIVLCPLFLLYMFGLFVSPWISLWRLIQQDYGVTAGDSSSKAHLQPALVVLYSLALFQGVLFYYRAISAWEE
  IRIS_313-10738     GAVRVLVFTIVLCPLFLLYMFGLFVSPWISLWRLIQQDYGVTAGDSSSKAHLQPALVVLYSLALFQGVLFYYRAISAWEE
b IRIS_313-8554      GAVRVLVFTIVLCPLFLLYMFGLFVSPWISLWRLIQQDYGVTAGDSSSKAHLQPALVVLYSLALFQGVLFYYRAISAWEE
  IRIS_313-11480     GAVRVLVFTIVLCPLFLLYMFGLFVSPWISLWRLIQQDYGVTAGDSSSKAHLQPALVVLYSLALFQGVLFYYRAISAWEE
  IRIS_313-8568      GAVRVLVFTIVLCPLFLLYMFGLFVSPWISLWRLIQQDYGVTAGDSSSKAHLQPALVVLYSLALFQGVLFYYRAISAWEE
  IRIS_313-8813      GAVRVLVFTIVLCPLFLLYMFGLFVSPWISLWRLIQQDYGVTAGDSSSKAHLQPALVVLYSLALFQGVLFYYRAISAWEE
```

```
                            250       260       270       280       290       300       310
                              .         .         .         .         .         .         .
  Nipponbare         QKLVKDVADKYMFDTVSRSSVSDYLHEIKVGCENDPSFARGRNLITYAVKLMESTSPDGYLSGARILDTLIKFNRD.DAS
  IRIS_313-7914      QKLVKDVADKYMFDTVSRSSVSDYLHEIKVGCENDPSFARGRNLITYAVKLMESTSPDGYLSGARILDTLIKFNRD.DAS
  AZUCENA            QKLVKDVADKYMFDTVSRSSVSDYLHEIKVGCENDPSFARGRNLITYAVKLMESTSPDGYLSGARILDTLIKFNRD.DAS
d YT16               QKLVKDVADKYMFDTVSRSSVSDYLHEIKVGCENDPSFARGRNLITYAVKLMESTSPDGYLSGARILDTLIKFNRD.DAS
  IRIS_313-11163     QKLVKDVADKYMFDTVSRSSVSDYLHEIKVGCENDPSFARGRNLITYAVKLMESTSPDGYLSGARILDTLIKFNRD.DAS
  IRIS_313-11127     QKLVKDVADKYMFDTVSRSSVSDYLHEIKVGCENDPSFARGRNLITYAVKLMESTSPDGYLSGARILDTLIKFNRD.DAS
  IRIS_313-11360     QKLVKDVADKYMFDTVSRSSVSDYLHEIKVGCENDPSFARGRNLITYAVKLMESTSPDGYLSGARILDTLIKFNRD.DAS
  BHA                QKLVKDVADKYMFDTVSRSSVSDYLHEIKVGCENDPSFARGRNLITYAVKLMESTSPDGYLSGARILDTLIKFNRD.DAS
  Katy               QKLVKDVADKYMFDTVSRSSVSDYLHEIKVGCENDPSFARGRNLITYAVKLMESTSPDGYLSGARILDTLIKFNRD.DAS
a IR64               QKLVKDVADKYMFDTVSRSSVSDYLHEIKVGCENDPSFARGRNLITYAVKLMESTSPDGYLSGARILDTLIKFNRD.DAS
  Pi4                QKLVKDVADKYMFDTVSRSSVSDYLHEIKVGCENDPSFARGRNLITYAVKLMESTSPDGYLSGARILDTLIKFNRD.DAS
  IRIS_313-11786     QKLVKDVADKYMFDTVSRSSVSDYLHEIKVGCENDPSFARGRNLITYAVKLMESTSPDGYLSGARILDTLIKFNRD.DAS
  Amane              QKLVKDVADKYMFDTVSRSSVSDYLHEIKVGCENDPSFARGRNLITYAVKLMESTSPDGYLSGARILDTLIKFNRD.DAS
e IRIS_313-11708     QKLVKDVADKYMFDTVSRSSVSDYLHEIKVGCENDPSFARGRNLITYAVKLMESTSPDGYLSGARILDTLIKFNRD.DAS
  IRIS_313-10841     QKLVKDVADKYMFDTVSRSSVSDYLHEIKVGCENDPSFARGRNLITYAVKLMESTSPDGYLSGARILDTLIKFNRD.DAS
  CO39               QKLVKDVADKYMFDTVSRSSVSDYLHEIKVGCENDPSFARGRNLITYAVKLMESTSPDGYLSGARILDTLIKFNRD.DAS
  IRIS_313-10879     QKLVKDVADKYMFDTVSRSSVSDYLHEIKVGCENDPSFARGRNLITYAVKLMESTSPDGYLSGARILDTLIKFNRD.DAS
  IRIS_313-10985     QKLVKDVADKYMFDTVSRSSVSDYLHEIKVGCENDPSFARGRNLITYAVKLMESTSPDGYLSGARILDTLIKFNRD.DAS
  IRIS_313-12048     QKLVKDVADKYMFDTVSRSSVSDYLHEIKVGCENDPSFARGRNLITYAVKLMESTSPDGYLSGARILDTLIKFNRD.DAS
c IRIS_313-12029     QKLVKDVADKYMFDTVSRSSVSDYLHEIKVGCENDPSFARGRNLITYAVKLMESTSPDGYLSGARILDTLIKFNRD.DAS
  IRIS_313-10059     QKLVKDVADKYMFDTVSRSSVSDYLHEIKVGCENDPSFARGRNLITYAVKLMESTSPDGYLSGARILDTLIKFNRD.DAS
  Pi1                QKLVKDVADKYMFDTVSRSSVSDYLHEIKVGCENDPSFARGRNLITYAVKLMESTSPDGYLSGARILDTLIKFNRD.DAS
  O.longistaminata   QKLVKDVADKYMFDTVSCSSVSDYLHEIKVGCENDPSFARGRNLITYAVKLMESTSPDGYLSGARILDTLIMFNRDADAS
  IRIS_313-12190     QKLVKDVADKYMFDTVSRSSVSDYLHEIKVGCENDPSFARGRNLITYAVKLMESTSPDGYLSGARILDTLIKFNRDADAS
  IRIS_313-10314     QKLVKDVADKYMFDTVSRSSVSDYLHEIKVGCENDPSFARGRNLITYAVKLMESTSPDGYLSGARILDTLIKFNRDADAS
  IRIS_313-10738     QKLVKDVADKYMFDTVSRSSVSDYLHEIKVGCENDPSFARGRNLITYAVKLMESTSPDGYLSGARILDTLIKFNRDADAS
b IRIS_313-8554      QKLVKDVADKYMFDTVSRSSVSDYLHEIKVGCENDPSFARGRNLITYAVKLMESTSPDGYLSGARILDTLIKFNRDADAS
  IRIS_313-11480     QKLVKDVADKYMFDTVSRSSVSDYLHEIKVGCENDPSFARGRNLITYAVKLMESTSPDGYLSGARILDTLIKFNRDADAS
  IRIS_313-8568      QKLVKDVADKYMFDTVSRSSVSDYLHEIKVGCENDPSFARGRNLITYAVKLMESTSPDGYLSGARILDTLIKFNRDADAS
  IRIS_313-8813      QKLVKDVADKYMFDTVSRSSVSDYLHEIKVGCENDPSFARGRNLITYAVKLMESTSPDGYLSGARILDTLIKFNRDADAS
```

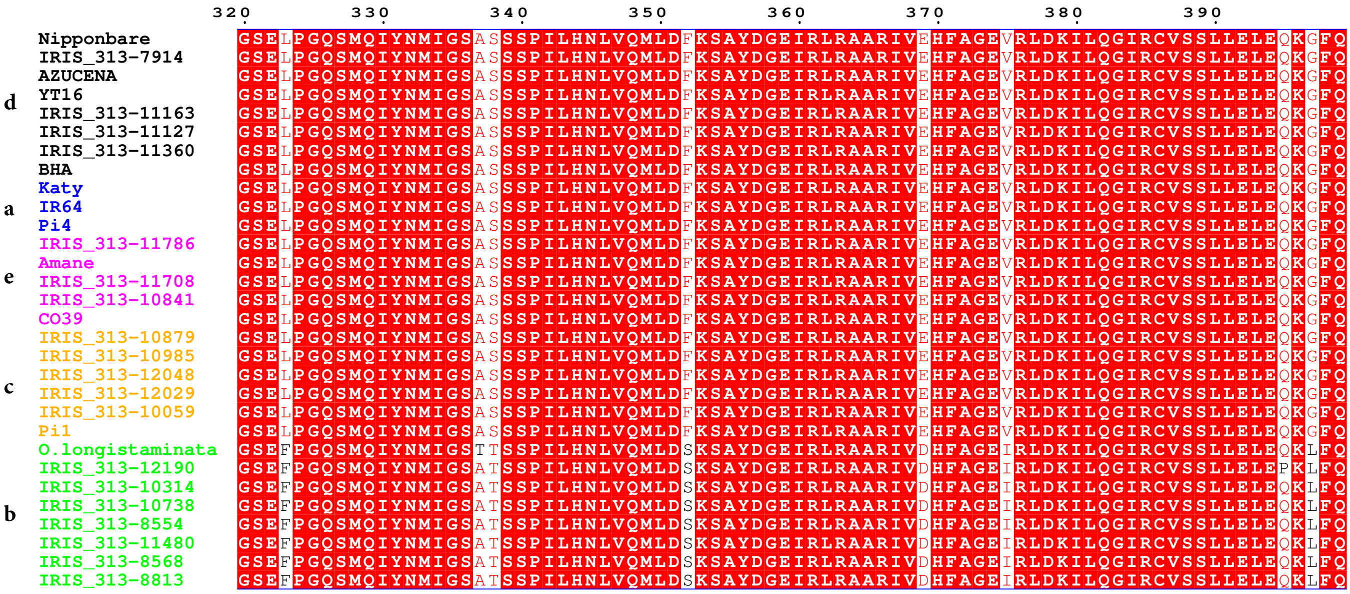

320 330 340 350 360 370 380 390
d
a
e
c
b
Nipponbare GSELPGQSMQIYNMIGSASSSPILHNLVQMLDFKSAYDGEIRLRAARIVEHFAGEVRLDKILQGIRCVSSLLELEQKGFQ
IRIS_313-7914 GSELPGQSMQIYNMIGSASSSPILHNLVQMLDFKSAYDGEIRLRAARIVEHFAGEVRLDKILQGIRCVSSLLELEQKGFQ
AZUCENA GSELPGQSMQIYNMIGSASSSPILHNLVQMLDFKSAYDGEIRLRAARIVEHFAGEVRLDKILQGIRCVSSLLELEQKGFQ
YT16 GSELPGQSMQIYNMIGSASSSPILHNLVQMLDFKSAYDGEIRLRAARIVEHFAGEVRLDKILQGIRCVSSLLELEQKGFQ
IRIS_313-11163 GSELPGQSMQIYNMIGSASSSPILHNLVQMLDFKSAYDGEIRLRAARIVEHFAGEVRLDKILQGIRCVSSLLELEQKGFQ
IRIS_313-11127 GSELPGQSMQIYNMIGSASSSPILHNLVQMLDFKSAYDGEIRLRAARIVEHFAGEVRLDKILQGIRCVSSLLELEQKGFQ
IRIS_313-11360 GSELPGQSMQIYNMIGSASSSPILHNLVQMLDFKSAYDGEIRLRAARIVEHFAGEVRLDKILQGIRCVSSLLELEQKGFQ
BHA GSELPGQSMQIYNMIGSASSSPILHNLVQMLDFKSAYDGEIRLRAARIVEHFAGEVRLDKILQGIRCVSSLLELEQKGFQ
Katy GSELPGQSMQIYNMIGSASSSPILHNLVQMLDFKSAYDGEIRLRAARIVEHFAGEVRLDKILQGIRCVSSLLELEQKGFQ
IR64 GSELPGQSMQIYNMIGSASSSPILHNLVQMLDFKSAYDGEIRLRAARIVEHFAGEVRLDKILQGIRCVSSLLELEQKGFQ
Pi4 GSELPGQSMQIYNMIGSASSSPILHNLVQMLDFKSAYDGEIRLRAARIVEHFAGEVRLDKILQGIRCVSSLLELEQKGFQ
IRIS_313-11786 GSELPGQSMQIYNMIGSASSSPILHNLVQMLDFKSAYDGEIRLRAARIVEHFAGEVRLDKILQGIRCVSSLLELEQKGFQ
Amane GSELPGQSMQIYNMIGSASSSPILHNLVQMLDFKSAYDGEIRLRAARIVEHFAGEVRLDKILQGIRCVSSLLELEQKGFQ
IRIS_313-11708 GSELPGQSMQIYNMIGSASSSPILHNLVQMLDFKSAYDGEIRLRAARIVEHFAGEVRLDKILQGIRCVSSLLELEQKGFQ
IRIS_313-10841 GSELPGQSMQIYNMIGSASSSPILHNLVQMLDFKSAYDGEIRLRAARIVEHFAGEVRLDKILQGIRCVSSLLELEQKGFQ
CO39 GSELPGQSMQIYNMIGSASSSPILHNLVQMLDFKSAYDGEIRLRAARIVEHFAGEVRLDKILQGIRCVSSLLELEQKGFQ
IRIS_313-10879 GSELPGQSMQIYNMIGSASSSPILHNLVQMLDFKSAYDGEIRLRAARIVEHFAGEVRLDKILQGIRCVSSLLELEQKGFQ
IRIS_313-10985 GSELPGQSMQIYNMIGSASSSPILHNLVQMLDFKSAYDGEIRLRAARIVEHFAGEVRLDKILQGIRCVSSLLELEQKGFQ
IRIS_313-12048 GSELPGQSMQIYNMIGSASSSPILHNLVQMLDFKSAYDGEIRLRAARIVEHFAGEVRLDKILQGIRCVSSLLELEQKGFQ
IRIS_313-12029 GSELPGQSMQIYNMIGSASSSPILHNLVQMLDFKSAYDGEIRLRAARIVEHFAGEVRLDKILQGIRCVSSLLELEQKGFQ
IRIS_313-10059 GSELPGQSMQIYNMIGSASSSPILHNLVQMLDFKSAYDGEIRLRAARIVEHFAGEVRLDKILQGIRCVSSLLELEQKGFQ
Pi1 GSELPGQSMQIYNMIGSASSSPILHNLVQMLDFKSAYDGEIRLRAARIVEHFAGEVRLDKILQGIRCVSSLLELEQKGFQ
O.longistaminata GSEFPGQSMQIYNMIGSTTSSPILHNLVQMLDSKSAYDGEIRLRAARIVDHFAGEIRLDKILQGIRCVSSLLELEQKLFQ
IRIS_313-12190 GSEFPGQSMQIYNMIGSATSSPILHNLVQMLDSKSAYDGEIRLRAARIVDHFAGEIRLDKILQGIRCVSSLLELEPKLFQ
IRIS_313-10314 GSEFPGQSMQIYNMIGSATSSPILHNLVQMLDSKSAYDGEIRLRAARIVDHFAGEIRLDKILQGIRCVSSLLELEQKLFQ
IRIS_313-10738 GSEFPGQSMQIYNMIGSATSSPILHNLVQMLDSKSAYDGEIRLRAARIVDHFAGEIRLDKILQGIRCVSSLLELEQKLFQ
IRIS_313-8554 GSEFPGQSMQIYNMIGSATSSPILHNLVQMLDSKSAYDGEIRLRAARIVDHFAGEIRLDKILQGIRCVSSLLELEQKLFQ
IRIS_313-11480 GSEFPGQSMQIYNMIGSATSSPILHNLVQMLDSKSAYDGEIRLRAARIVDHFAGEIRLDKILQGIRCVSSLLELEQKLFQ
IRIS_313-8568 GSEFPGQSMQIYNMIGSATSSPILHNLVQMLDSKSAYDGEIRLRAARIVDHFAGEIRLDKILQGIRCVSSLLELEQKLFQ
IRIS_313-8813 GSEFPGQSMQIYNMIGSATSSPILHNLVQMLDSKSAYDGEIRLRAARIVDHFAGEIRLDKILQGIRCVSSLLELEQKLFQ

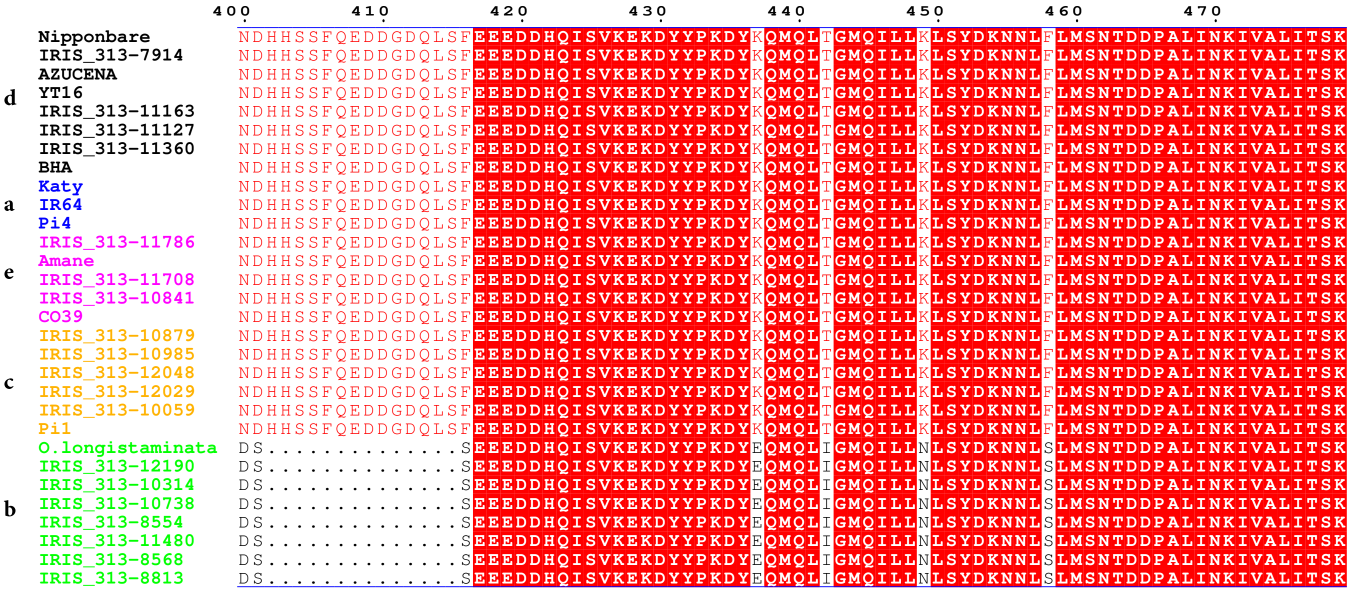

400 410 420 430 440 450 460 470
d
a
e
c
b
Nipponbare NDHHSSFQEDDGDQLSFEEEDDHQISVKEKDYYPKDYKQMQLTGMQILLKLSYDKNNLFLMSNTDDPALINKIVALITSK
IRIS_313-7914 NDHHSSFQEDDGDQLSFEEEDDHQISVKEKDYYPKDYKQMQLTGMQILLKLSYDKNNLFLMSNTDDPALINKIVALITSK
AZUCENA NDHHSSFQEDDGDQLSFEEEDDHQISVKEKDYYPKDYKQMQLTGMQILLKLSYDKNNLFLMSNTDDPALINKIVALITSK
YT16 NDHHSSFQEDDGDQLSFEEEDDHQISVKEKDYYPKDYKQMQLTGMQILLKLSYDKNNLFLMSNTDDPALINKIVALITSK
IRIS_313-11163 NDHHSSFQEDDGDQLSFEEEDDHQISVKEKDYYPKDYKQMQLTGMQILLKLSYDKNNLFLMSNTDDPALINKIVALITSK
IRIS_313-11127 NDHHSSFQEDDGDQLSFEEEDDHQISVKEKDYYPKDYKQMQLTGMQILLKLSYDKNNLFLMSNTDDPALINKIVALITSK
IRIS_313-11360 NDHHSSFQEDDGDQLSFEEEDDHQISVKEKDYYPKDYKQMQLTGMQILLKLSYDKNNLFLMSNTDDPALINKIVALITSK
BHA NDHHSSFQEDDGDQLSFEEEDDHQISVKEKDYYPKDYKQMQLTGMQILLKLSYDKNNLFLMSNTDDPALINKIVALITSK
Katy NDHHSSFQEDDGDQLSFEEEDDHQISVKEKDYYPKDYKQMQLTGMQILLKLSYDKNNLFLMSNTDDPALINKIVALITSK
IR64 NDHHSSFQEDDGDQLSFEEEDDHQISVKEKDYYPKDYKQMQLTGMQILLKLSYDKNNLFLMSNTDDPALINKIVALITSK
Pi4 NDHHSSFQEDDGDQLSFEEEDDHQISVKEKDYYPKDYKQMQLTGMQILLKLSYDKNNLFLMSNTDDPALINKIVALITSK
IRIS_313-11786 NDHHSSFQEDDGDQLSFEEEDDHQISVKEKDYYPKDYKQMQLTGMQILLKLSYDKNNLFLMSNTDDPALINKIVALITSK
Amane NDHHSSFQEDDGDQLSFEEEDDHQISVKEKDYYPKDYKQMQLTGMQILLKLSYDKNNLFLMSNTDDPALINKIVALITSK
IRIS_313-11708 NDHHSSFQEDDGDQLSFEEEDDHQISVKEKDYYPKDYKQMQLTGMQILLKLSYDKNNLFLMSNTDDPALINKIVALITSK
IRIS_313-10841 NDHHSSFQEDDGDQLSFEEEDDHQISVKEKDYYPKDYKQMQLTGMQILLKLSYDKNNLFLMSNTDDPALINKIVALITSK
CO39 NDHHSSFQEDDGDQLSFEEEDDHQISVKEKDYYPKDYKQMQLTGMQILLKLSYDKNNLFLMSNTDDPALINKIVALITSK
IRIS_313-10879 NDHHSSFQEDDGDQLSFEEEDDHQISVKEKDYYPKDYKQMQLTGMQILLKLSYDKNNLFLMSNTDDPALINKIVALITSK
IRIS_313-10985 NDHHSSFQEDDGDQLSFEEEDDHQISVKEKDYYPKDYKQMQLTGMQILLKLSYDKNNLFLMSNTDDPALINKIVALITSK
IRIS_313-12048 NDHHSSFQEDDGDQLSFEEEDDHQISVKEKDYYPKDYKQMQLTGMQILLKLSYDKNNLFLMSNTDDPALINKIVALITSK
IRIS_313-12029 NDHHSSFQEDDGDQLSFEEEDDHQISVKEKDYYPKDYKQMQLTGMQILLKLSYDKNNLFLMSNTDDPALINKIVALITSK
IRIS_313-10059 NDHHSSFQEDDGDQLSFEEEDDHQISVKEKDYYPKDYKQMQLTGMQILLKLSYDKNNLFLMSNTDDPALINKIVALITSK
Pi1 NDHHSSFQEDDGDQLSFEEEDDHQISVKEKDYYPKDYKQMQLTGMQILLKLSYDKNNLFLMSNTDDPALINKIVALITSK
O.longistaminata DS..............SEEEDDHQISVKEKDYYPKDYEQMQLIGMQILLNLSYDKNNLSLMSNTDDPALINKIVALITSK
IRIS_313-12190 DS..............SEEEDDHQISVKEKDYYPKDYEQMQLIGMQILLNLSYDKNNLSLMSNTDDPALINKIVALITSK
IRIS_313-10314 DS..............SEEEDDHQISVKEKDYYPKDYEQMQLIGMQILLNLSYDKNNLSLMSNTDDPALINKIVALITSK
IRIS_313-10738 DS..............SEEEDDHQISVKEKDYYPKDYEQMQLIGMQILLNLSYDKNNLSLMSNTDDPALINKIVALITSK
IRIS_313-8554 DS..............SEEEDDHQISVKEKDYYPKDYEQMQLIGMQILLNLSYDKNNLSLMSNTDDPALINKIVALITSK
IRIS_313-11480 DS..............SEEEDDHQISVKEKDYYPKDYEQMQLIGMQILLNLSYDKNNLSLMSNTDDPALINKIVALITSK
IRIS_313-8568 DS..............SEEEDDHQISVKEKDYYPKDYEQMQLIGMQILLNLSYDKNNLSLMSNTDDPALINKIVALITSK
IRIS_313-8813 DS..............SEEEDDHQISVKEKDYYPKDYEQMQLIGMQILLNLSYDKNNLSLMSNTDDPALINKIVALITSK

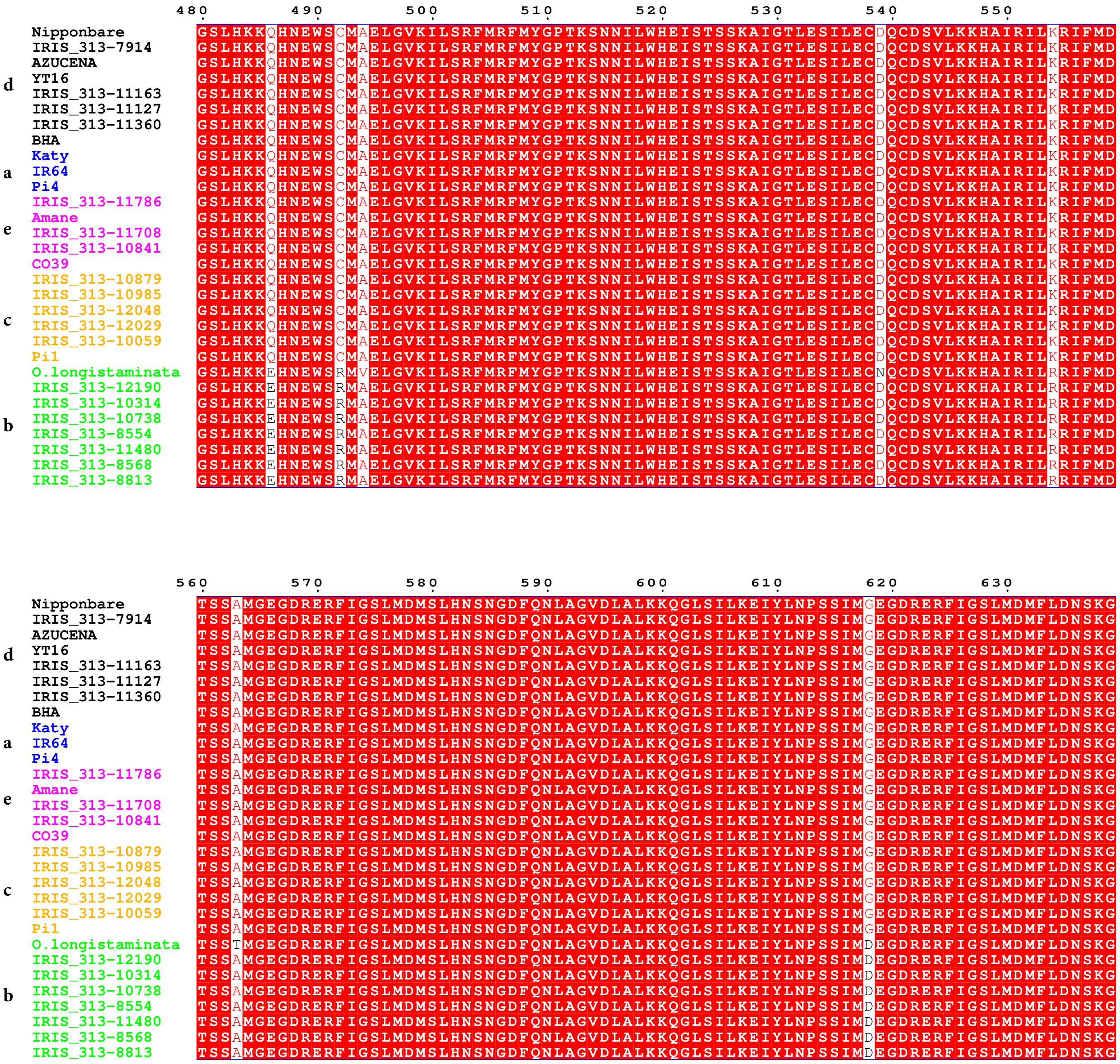
480 490 500 510 520 530 540 550
Nipponbare GSLHKKQHNEWSCMAELGVKILSRFMRFMYGPTKSNNILWHEISTSSKAIGTLESILECDQCDSVLKKHAIRILKRIFMD
IRIS_313-7914 GSLHKKQHNEWSCMAELGVKILSRFMRFMYGPTKSNNILWHEISTSSKAIGTLESILECDQCDSVLKKHAIRILKRIFMD
AZUCENA GSLHKKQHNEWSCMAELGVKILSRFMRFMYGPTKSNNILWHEISTSSKAIGTLESILECDQCDSVLKKHAIRILKRIFMD
d
YT16 GSLHKKQHNEWSCMAELGVKILSRFMRFMYGPTKSNNILWHEISTSSKAIGTLESILECDQCDSVLKKHAIRILKRIFMD
IRIS_313-11163 GSLHKKQHNEWSCMAELGVKILSRFMRFMYGPTKSNNILWHEISTSSKAIGTLESILECDQCDSVLKKHAIRILKRIFMD
IRIS_313-11127 GSLHKKQHNEWSCMAELGVKILSRFMRFMYGPTKSNNILWHEISTSSKAIGTLESILECDQCDSVLKKHAIRILKRIFMD
IRIS_313-11360 GSLHKKQHNEWSCMAELGVKILSRFMRFMYGPTKSNNILWHEISTSSKAIGTLESILECDQCDSVLKKHAIRILKRIFMD
BHA GSLHKKQHNEWSCMAELGVKILSRFMRFMYGPTKSNNILWHEISTSSKAIGTLESILECDQCDSVLKKHAIRILKRIFMD
Katy GSLHKKQHNEWSCMAELGVKILSRFMRFMYGPTKSNNILWHEISTSSKAIGTLESILECDQCDSVLKKHAIRILKRIFMD
a
IR64 GSLHKKQHNEWSCMAELGVKILSRFMRFMYGPTKSNNILWHEISTSSKAIGTLESILECDQCDSVLKKHAIRILKRIFMD
Pi4 GSLHKKQHNEWSCMAELGVKILSRFMRFMYGPTKSNNILWHEISTSSKAIGTLESILECDQCDSVLKKHAIRILKRIFMD
IRIS_313-11786 GSLHKKQHNEWSCMAELGVKILSRFMRFMYGPTKSNNILWHEISTSSKAIGTLESILECDQCDSVLKKHAIRILKRIFMD
Amane GSLHKKQHNEWSCMAELGVKILSRFMRFMYGPTKSNNILWHEISTSSKAIGTLESILECDQCDSVLKKHAIRILKRIFMD
e
IRIS_313-11708 GSLHKKQHNEWSCMAELGVKILSRFMRFMYGPTKSNNILWHEISTSSKAIGTLESILECDQCDSVLKKHAIRILKRIFMD
IRIS_313-10841 GSLHKKQHNEWSCMAELGVKILSRFMRFMYGPTKSNNILWHEISTSSKAIGTLESILECDQCDSVLKKHAIRILKRIFMD
CO39 GSLHKKQHNEWSCMAELGVKILSRFMRFMYGPTKSNNILWHEISTSSKAIGTLESILECDQCDSVLKKHAIRILKRIFMD
IRIS_313-10879 GSLHKKQHNEWSCMAELGVKILSRFMRFMYGPTKSNNILWHEISTSSKAIGTLESILECDQCDSVLKKHAIRILKRIFMD
IRIS_313-10985 GSLHKKQHNEWSCMAELGVKILSRFMRFMYGPTKSNNILWHEISTSSKAIGTLESILECDQCDSVLKKHAIRILKRIFMD
IRIS_313-12048 GSLHKKQHNEWSCMAELGVKILSRFMRFMYGPTKSNNILWHEISTSSKAIGTLESILECDQCDSVLKKHAIRILKRIFMD
c
IRIS_313-12029 GSLHKKQHNEWSCMAELGVKILSRFMRFMYGPTKSNNILWHEISTSSKAIGTLESILECDQCDSVLKKHAIRILKRIFMD
IRIS_313-10059 GSLHKKQHNEWSCMAELGVKILSRFMRFMYGPTKSNNILWHEISTSSKAIGTLESILECDQCDSVLKKHAIRILKRIFMD
Pi1 GSLHKKQHNEWSCMAELGVKILSRFMRFMYGPTKSNNILWHEISTSSKAIGTLESILECDQCDSVLKKHAIRILKRIFMD
O.longistaminata GSLHKKEHNEWSRMVELGVKILSRFMRFMYGPTKSNNILWHEISTSSKAIGTLESILECNQCDSVLKKHAIRILRRIFMD
IRIS_313-12190 GSLHKKEHNEWSRMAELGVKILSRFMRFMYGPTKSNNILWHEISTSSKAIGTLESILECDQCDSVLKKHAIRILRRIFMD
IRIS_313-10314 GSLHKKEHNEWSRMAELGVKILSRFMRFMYGPTKSNNILWHEISTSSKAIGTLESILECDQCDSVLKKHAIRILRRIFMD
b
IRIS_313-10738 GSLHKKEHNEWSRMAELGVKILSRFMRFMYGPTKSNNILWHEISTSSKAIGTLESILECDQCDSVLKKHAIRILRRIFMD
IRIS_313-8554 GSLHKKEHNEWSRMAELGVKILSRFMRFMYGPTKSNNILWHEISTSSKAIGTLESILECDQCDSVLKKHAIRILRRIFMD
IRIS_313-11480 GSLHKKEHNEWSRMAELGVKILSRFMRFMYGPTKSNNILWHEISTSSKAIGTLESILECDQCDSVLKKHAIRILRRIFMD
IRIS_313-8568 GSLHKKEHNEWSRMAELGVKILSRFMRFMYGPTKSNNILWHEISTSSKAIGTLESILECDQCDSVLKKHAIRILRRIFMD
IRIS_313-8813 GSLHKKEHNEWSRMAELGVKILSRFMRFMYGPTKSNNILWHEISTSSKAIGTLESILECDQCDSVLKKHAIRILRRIFMD
560 570 580 590 600 610 620 630
Nipponbare TSSAMGEGDRERFIGSLMDMSLHNSNGDFQNLAGVDLALKKQGLSILKEIYLNPSSIMGEGDRERFIGSLMDMFLDNSKG
IRIS_313-7914 TSSAMGEGDRERFIGSLMDMSLHNSNGDFQNLAGVDLALKKQGLSILKEIYLNPSSIMGEGDRERFIGSLMDMFLDNSKG
AZUCENA TSSAMGEGDRERFIGSLMDMSLHNSNGDFQNLAGVDLALKKQGLSILKEIYLNPSSIMGEGDRERFIGSLMDMFLDNSKG
d
YT16 TSSAMGEGDRERFIGSLMDMSLHNSNGDFQNLAGVDLALKKQGLSILKEIYLNPSSIMGEGDRERFIGSLMDMFLDNSKG
IRIS_313-11163 TSSAMGEGDRERFIGSLMDMSLHNSNGDFQNLAGVDLALKKQGLSILKEIYLNPSSIMGEGDRERFIGSLMDMFLDNSKG
IRIS_313-11127 TSSAMGEGDRERFIGSLMDMSLHNSNGDFQNLAGVDLALKKQGLSILKEIYLNPSSIMGEGDRERFIGSLMDMFLDNSKG
IRIS_313-11360 TSSAMGEGDRERFIGSLMDMSLHNSNGDFQNLAGVDLALKKQGLSILKEIYLNPSSIMGEGDRERFIGSLMDMFLDNSKG
BHA TSSAMGEGDRERFIGSLMDMSLHNSNGDFQNLAGVDLALKKQGLSILKEIYLNPSSIMGEGDRERFIGSLMDMFLDNSKG
Katy TSSAMGEGDRERFIGSLMDMSLHNSNGDFQNLAGVDLALKKQGLSILKEIYLNPSSIMGEGDRERFIGSLMDMFLDNSKG
a
IR64 TSSAMGEGDRERFIGSLMDMSLHNSNGDFQNLAGVDLALKKQGLSILKEIYLNPSSIMGEGDRERFIGSLMDMFLDNSKG
Pi4 TSSAMGEGDRERFIGSLMDMSLHNSNGDFQNLAGVDLALKKQGLSILKEIYLNPSSIMGEGDRERFIGSLMDMFLDNSKG
IRIS_313-11786 TSSAMGEGDRERFIGSLMDMSLHNSNGDFQNLAGVDLALKKQGLSILKEIYLNPSSIMGEGDRERFIGSLMDMFLDNSKG
Amane TSSAMGEGDRERFIGSLMDMSLHNSNGDFQNLAGVDLALKKQGLSILKEIYLNPSSIMGEGDRERFIGSLMDMFLDNSKG
e
IRIS_313-11708 TSSAMGEGDRERFIGSLMDMSLHNSNGDFQNLAGVDLALKKQGLSILKEIYLNPSSIMGEGDRERFIGSLMDMFLDNSKG
IRIS_313-10841 TSSAMGEGDRERFIGSLMDMSLHNSNGDFQNLAGVDLALKKQGLSILKEIYLNPSSIMGEGDRERFIGSLMDMFLDNSKG
CO39 TSSAMGEGDRERFIGSLMDMSLHNSNGDFQNLAGVDLALKKQGLSILKEIYLNPSSIMGEGDRERFIGSLMDMFLDNSKG
IRIS_313-10879 TSSAMGEGDRERFIGSLMDMSLHNSNGDFQNLAGVDLALKKQGLSILKEIYLNPSSIMGEGDRERFIGSLMDMFLDNSKG
IRIS_313-10985 TSSAMGEGDRERFIGSLMDMSLHNSNGDFQNLAGVDLALKKQGLSILKEIYLNPSSIMGEGDRERFIGSLMDMFLDNSKG
IRIS_313-12048 TSSAMGEGDRERFIGSLMDMSLHNSNGDFQNLAGVDLALKKQGLSILKEIYLNPSSIMGEGDRERFIGSLMDMFLDNSKG
c
IRIS_313-12029 TSSAMGEGDRERFIGSLMDMSLHNSNGDFQNLAGVDLALKKQGLSILKEIYLNPSSIMGEGDRERFIGSLMDMFLDNSKG
IRIS_313-10059 TSSAMGEGDRERFIGSLMDMSLHNSNGDFQNLAGVDLALKKQGLSILKEIYLNPSSIMGEGDRERFIGSLMDMFLDNSKG
Pi1 TSSAMGEGDRERFIGSLMDMSLHNSNGDFQNLAGVDLALKKQGLSILKEIYLNPSSIMGEGDRERFIGSLMDMFLDNSKG
O.longistaminata TSSTMGEGDRERFIGSLMDMSLHNSNGDFQNLAGVDLALKKQGLSILKEIYLNPSSIMDEGDRERFIGSLMDMFLDNSKG
IRIS_313-12190 TSSAMGEGDRERFIGSLMDMSLHNSNGDFQNLAGVDLALKKQGLSILKEIYLNPSSIMDEGDRERFIGSLMDMFLDNSKG
IRIS_313-10314 TSSAMGEGDRERFIGSLMDMSLHNSNGDFQNLAGVDLALKKQGLSILKEIYLNPSSIMDEGDRERFIGSLMDMFLDNSKG
b
IRIS_313-10738 TSSAMGEGDRERFIGSLMDMSLHNSNGDFQNLAGVDLALKKQGLSILKEIYLNPSSIMDEGDRERFIGSLMDMFLDNSKG
IRIS_313-8554 TSSAMGEGDRERFIGSLMDMSLHNSNGDFQNLAGVDLALKKQGLSILKEIYLNPSSIMDEGDRERFIGSLMDMFLDNSKG
IRIS_313-11480 TSSAMGEGDRERFIGSLMDMSLHNSNGDFQNLAGVDLALKKQGLSILKEIYLNPSSIMDEGDRERFIGSLMDMFLDNSKG
IRIS_313-8568 TSSAMGEGDRERFIGSLMDMSLHNSNGDFQNLAGVDLALKKQGLSILKEIYLNPSSIMDEGDRERFIGSLMDMFLDNSKG
IRIS_313-8813 TSSAMGEGDRERFIGSLMDMSLHNSNGDFQNLAGVDLALKKQGLSILKEIYLNPSSIMDEGDRERFIGSLMDMFLDNSKG

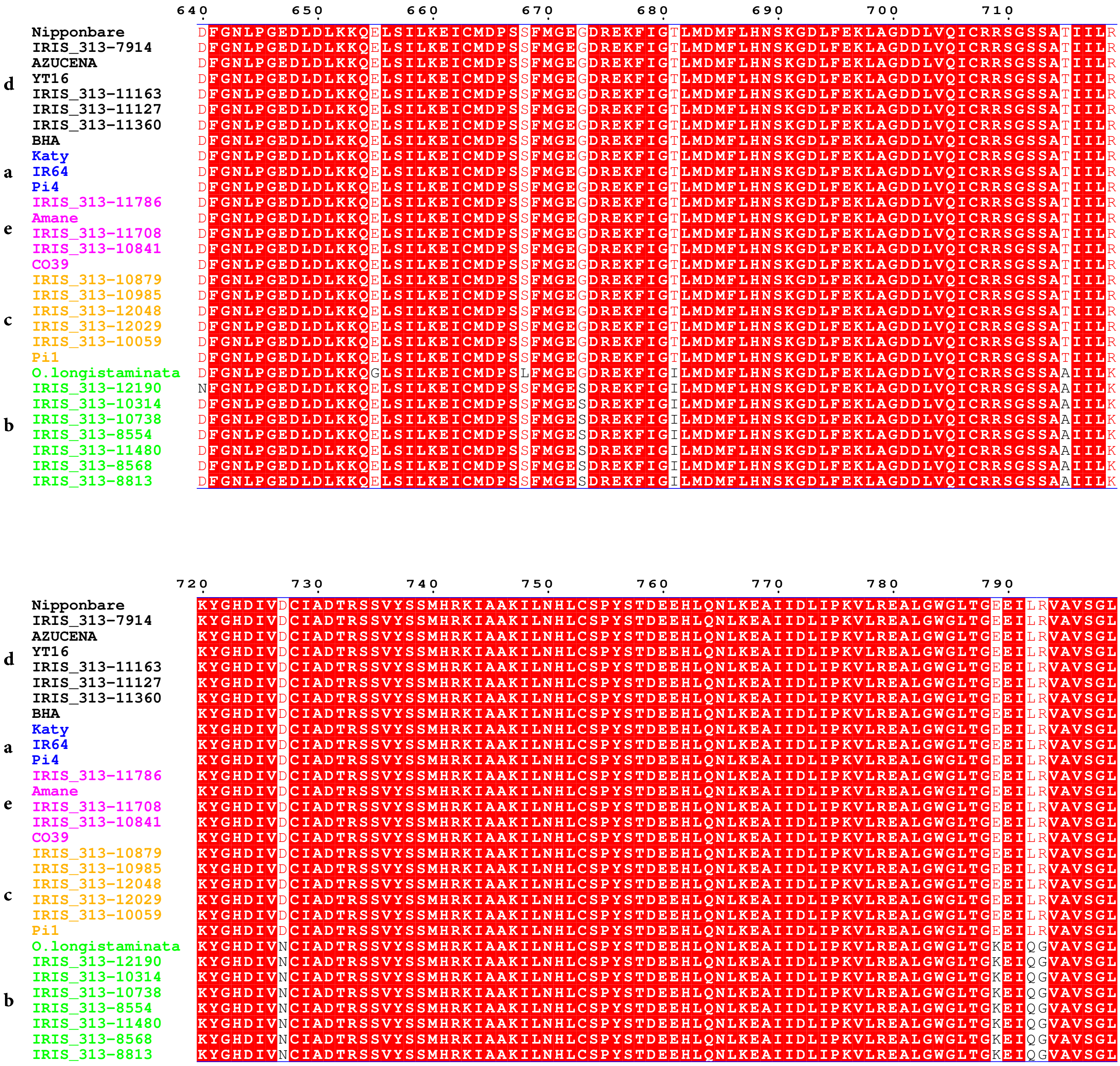
640 650 660 670 680 690 700 710
Nipponbare DFGNLPGEDLDLKKQELSILKEICMDPSSFMGEGDREKFIGTLMDMFLHNSKGDLFEKLAGDDLVQICRRSGSSATIILR
IRIS_313-7914 DFGNLPGEDLDLKKQELSILKEICMDPSSFMGEGDREKFIGTLMDMFLHNSKGDLFEKLAGDDLVQICRRSGSSATIILR
AZUCENA DFGNLPGEDLDLKKQELSILKEICMDPSSFMGEGDREKFIGTLMDMFLHNSKGDLFEKLAGDDLVQICRRSGSSATIILR
d YT16 DFGNLPGEDLDLKKQELSILKEICMDPSSFMGEGDREKFIGTLMDMFLHNSKGDLFEKLAGDDLVQICRRSGSSATIILR
IRIS_313-11163 DFGNLPGEDLDLKKQELSILKEICMDPSSFMGEGDREKFIGTLMDMFLHNSKGDLFEKLAGDDLVQICRRSGSSATIILR
IRIS_313-11127 DFGNLPGEDLDLKKQELSILKEICMDPSSFMGEGDREKFIGTLMDMFLHNSKGDLFEKLAGDDLVQICRRSGSSATIILR
IRIS_313-11360 DFGNLPGEDLDLKKQELSILKEICMDPSSFMGEGDREKFIGTLMDMFLHNSKGDLFEKLAGDDLVQICRRSGSSATIILR
BHA DFGNLPGEDLDLKKQELSILKEICMDPSSFMGEGDREKFIGTLMDMFLHNSKGDLFEKLAGDDLVQICRRSGSSATIILR
Katy DFGNLPGEDLDLKKQELSILKEICMDPSSFMGEGDREKFIGTLMDMFLHNSKGDLFEKLAGDDLVQICRRSGSSATIILR
a IR64 DFGNLPGEDLDLKKQELSILKEICMDPSSFMGEGDREKFIGTLMDMFLHNSKGDLFEKLAGDDLVQICRRSGSSATIILR
Pi4 DFGNLPGEDLDLKKQELSILKEICMDPSSFMGEGDREKFIGTLMDMFLHNSKGDLFEKLAGDDLVQICRRSGSSATIILR
IRIS_313-11786 DFGNLPGEDLDLKKQELSILKEICMDPSSFMGEGDREKFIGTLMDMFLHNSKGDLFEKLAGDDLVQICRRSGSSATIILR
Amane DFGNLPGEDLDLKKQELSILKEICMDPSSFMGEGDREKFIGTLMDMFLHNSKGDLFEKLAGDDLVQICRRSGSSATIILR
e IRIS_313-11708 DFGNLPGEDLDLKKQELSILKEICMDPSSFMGEGDREKFIGTLMDMFLHNSKGDLFEKLAGDDLVQICRRSGSSATIILR
IRIS_313-10841 DFGNLPGEDLDLKKQELSILKEICMDPSSFMGEGDREKFIGTLMDMFLHNSKGDLFEKLAGDDLVQICRRSGSSATIILR
CO39 DFGNLPGEDLDLKKQELSILKEICMDPSSFMGEGDREKFIGTLMDMFLHNSKGDLFEKLAGDDLVQICRRSGSSATIILR
IRIS_313-10879 DFGNLPGEDLDLKKQELSILKEICMDPSSFMGEGDREKFIGTLMDMFLHNSKGDLFEKLAGDDLVQICRRSGSSATIILR
IRIS_313-10985 DFGNLPGEDLDLKKQELSILKEICMDPSSFMGEGDREKFIGTLMDMFLHNSKGDLFEKLAGDDLVQICRRSGSSATIILR
c IRIS_313-12048 DFGNLPGEDLDLKKQELSILKEICMDPSSFMGEGDREKFIGTLMDMFLHNSKGDLFEKLAGDDLVQICRRSGSSATIILR
IRIS_313-12029 DFGNLPGEDLDLKKQELSILKEICMDPSSFMGEGDREKFIGTLMDMFLHNSKGDLFEKLAGDDLVQICRRSGSSATIILR
IRIS_313-10059 DFGNLPGEDLDLKKQELSILKEICMDPSSFMGEGDREKFIGTLMDMFLHNSKGDLFEKLAGDDLVQICRRSGSSATIILR
Pi1 DFGNLPGEDLDLKKQELSILKEICMDPSSFMGEGDREKFIGTLMDMFLHNSKGDLFEKLAGDDLVQICRRSGSSATIILR
O.longistaminata DFGNLPGEDLDLKKQGLSILKEICMDPSLFMGEGDREKFIGILMDMFLHNSKGDLFEKLAGDDLVQICRRSGSSAAIILK
IRIS_313-12190 NFGNLPGEDLDLKKQELSILKEICMDPSSFMGESDREKFIGILMDMFLHNSKGDLFEKLAGDDLVQICRRSGSSAAIILK
IRIS_313-10314 DFGNLPGEDLDLKKQELSILKEICMDPSSFMGESDREKFIGILMDMFLHNSKGDLFEKLAGDDLVQICRRSGSSAAIILK
b IRIS_313-10738 DFGNLPGEDLDLKKQELSILKEICMDPSSFMGESDREKFIGILMDMFLHNSKGDLFEKLAGDDLVQICRRSGSSAAIILK
IRIS_313-8554 DFGNLPGEDLDLKKQELSILKEICMDPSSFMGESDREKFIGILMDMFLHNSKGDLFEKLAGDDLVQICRRSGSSAAIILK
IRIS_313-11480 DFGNLPGEDLDLKKQELSILKEICMDPSSFMGESDREKFIGILMDMFLHNSKGDLFEKLAGDDLVQICRRSGSSAAIILK
IRIS_313-8568 DFGNLPGEDLDLKKQELSILKEICMDPSSFMGESDREKFIGILMDMFLHNSKGDLFEKLAGDDLVQICRRSGSSAAIILK
IRIS_313-8813 DFGNLPGEDLDLKKQELSILKEICMDPSSFMGESDREKFIGILMDMFLHNSKGDLFEKLAGDDLVQICRRSGSSAAIILK
720 730 740 750 760 770 780 790
Nipponbare KYGHDIVDCIADTRSSVYSSMHRKIAAKILNHLCSPYSTDEEHLQNLKEAIIDLIPKVLREALGWGLTGEEILRVAVSGL
IRIS_313-7914 KYGHDIVDCIADTRSSVYSSMHRKIAAKILNHLCSPYSTDEEHLQNLKEAIIDLIPKVLREALGWGLTGEEILRVAVSGL
AZUCENA KYGHDIVDCIADTRSSVYSSMHRKIAAKILNHLCSPYSTDEEHLQNLKEAIIDLIPKVLREALGWGLTGEEILRVAVSGL
d YT16 KYGHDIVDCIADTRSSVYSSMHRKIAAKILNHLCSPYSTDEEHLQNLKEAIIDLIPKVLREALGWGLTGEEILRVAVSGL
IRIS_313-11163 KYGHDIVDCIADTRSSVYSSMHRKIAAKILNHLCSPYSTDEEHLQNLKEAIIDLIPKVLREALGWGLTGEEILRVAVSGL
IRIS_313-11127 KYGHDIVDCIADTRSSVYSSMHRKIAAKILNHLCSPYSTDEEHLQNLKEAIIDLIPKVLREALGWGLTGEEILRVAVSGL
IRIS_313-11360 KYGHDIVDCIADTRSSVYSSMHRKIAAKILNHLCSPYSTDEEHLQNLKEAIIDLIPKVLREALGWGLTGEEILRVAVSGL
BHA KYGHDIVDCIADTRSSVYSSMHRKIAAKILNHLCSPYSTDEEHLQNLKEAIIDLIPKVLREALGWGLTGEEILRVAVSGL
Katy KYGHDIVDCIADTRSSVYSSMHRKIAAKILNHLCSPYSTDEEHLQNLKEAIIDLIPKVLREALGWGLTGEEILRVAVSGL
a IR64 KYGHDIVDCIADTRSSVYSSMHRKIAAKILNHLCSPYSTDEEHLQNLKEAIIDLIPKVLREALGWGLTGEEILRVAVSGL
Pi4 KYGHDIVDCIADTRSSVYSSMHRKIAAKILNHLCSPYSTDEEHLQNLKEAIIDLIPKVLREALGWGLTGEEILRVAVSGL
IRIS_313-11786 KYGHDIVDCIADTRSSVYSSMHRKIAAKILNHLCSPYSTDEEHLQNLKEAIIDLIPKVLREALGWGLTGEEILRVAVSGL
Amane KYGHDIVDCIADTRSSVYSSMHRKIAAKILNHLCSPYSTDEEHLQNLKEAIIDLIPKVLREALGWGLTGEEILRVAVSGL
e IRIS_313-11708 KYGHDIVDCIADTRSSVYSSMHRKIAAKILNHLCSPYSTDEEHLQNLKEAIIDLIPKVLREALGWGLTGEEILRVAVSGL
IRIS_313-10841 KYGHDIVDCIADTRSSVYSSMHRKIAAKILNHLCSPYSTDEEHLQNLKEAIIDLIPKVLREALGWGLTGEEILRVAVSGL
CO39 KYGHDIVDCIADTRSSVYSSMHRKIAAKILNHLCSPYSTDEEHLQNLKEAIIDLIPKVLREALGWGLTGEEILRVAVSGL
IRIS_313-10879 KYGHDIVDCIADTRSSVYSSMHRKIAAKILNHLCSPYSTDEEHLQNLKEAIIDLIPKVLREALGWGLTGEEILRVAVSGL
IRIS_313-10985 KYGHDIVDCIADTRSSVYSSMHRKIAAKILNHLCSPYSTDEEHLQNLKEAIIDLIPKVLREALGWGLTGEEILRVAVSGL
c IRIS_313-12048 KYGHDIVDCIADTRSSVYSSMHRKIAAKILNHLCSPYSTDEEHLQNLKEAIIDLIPKVLREALGWGLTGEEILRVAVSGL
IRIS_313-12029 KYGHDIVDCIADTRSSVYSSMHRKIAAKILNHLCSPYSTDEEHLQNLKEAIIDLIPKVLREALGWGLTGEEILRVAVSGL
IRIS_313-10059 KYGHDIVDCIADTRSSVYSSMHRKIAAKILNHLCSPYSTDEEHLQNLKEAIIDLIPKVLREALGWGLTGEEILRVAVSGL
Pi1 KYGHDIVDCIADTRSSVYSSMHRKIAAKILNHLCSPYSTDEEHLQNLKEAIIDLIPKVLREALGWGLTGEEILRVAVSGL
O.longistaminata KYGHDIVNCIADTRSSVYSSMHRKIAAKILNHLCSPYSTDEEHLQNLKEAIIDLIPKVLREALGWGLTGKEIQGVAVSGL
IRIS_313-12190 KYGHDIVNCIADTRSSVYSSMHRKIAAKILNHLCSPYSTDEEHLQNLKEAIIDLIPKVLREALGWGLTGKEIQGVAVSGL
IRIS_313-10314 KYGHDIVNCIADTRSSVYSSMHRKIAAKILNHLCSPYSTDEEHLQNLKEAIIDLIPKVLREALGWGLTGKEIQGVAVSGL
b IRIS_313-10738 KYGHDIVNCIADTRSSVYSSMHRKIAAKILNHLCSPYSTDEEHLQNLKEAIIDLIPKVLREALGWGLTGKEIQGVAVSGL
IRIS_313-8554 KYGHDIVNCIADTRSSVYSSMHRKIAAKILNHLCSPYSTDEEHLQNLKEAIIDLIPKVLREALGWGLTGKEIQGVAVSGL
IRIS_313-11480 KYGHDIVNCIADTRSSVYSSMHRKIAAKILNHLCSPYSTDEEHLQNLKEAIIDLIPKVLREALGWGLTGKEIQGVAVSGL
IRIS_313-8568 KYGHDIVNCIADTRSSVYSSMHRKIAAKILNHLCSPYSTDEEHLQNLKEAIIDLIPKVLREALGWGLTGKEIQGVAVSGL
IRIS_313-8813 KYGHDIVNCIADTRSSVYSSMHRKIAAKILNHLCSPYSTDEEHLQNLKEAIIDLIPKVLREALGWGLTGKEIQGVAVSGL

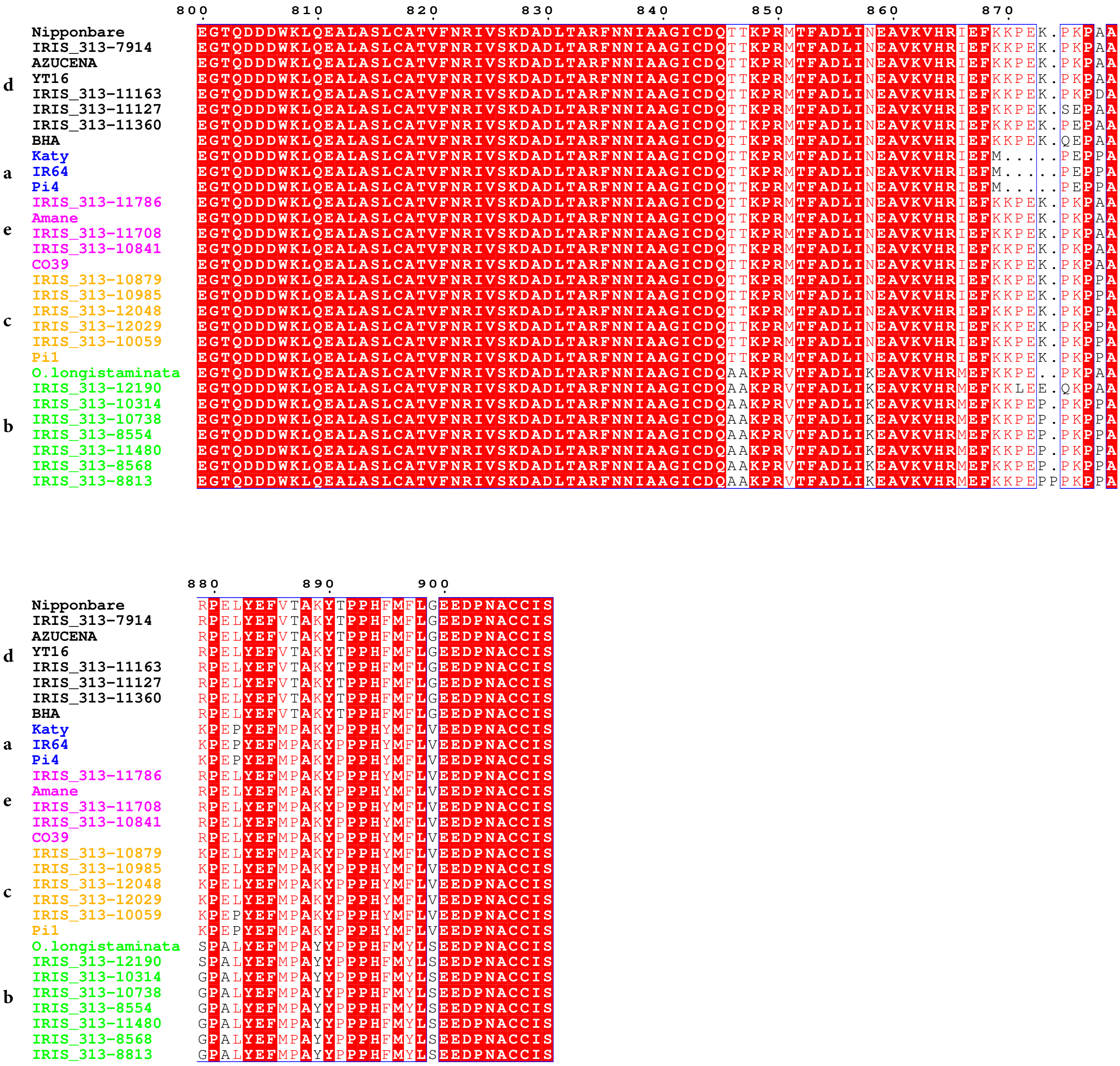

Supplementary Figure 4. Ptr amino acid alignment. Ptr amino acid alignment from the 10 accessions sequenced in this article, plus BHA, YT16, Pi1, Pi4, Amane, and Katy, CO39, Nipponbare, O. longistaminata and 12 other diverse rice genomes. Subgroup classifications for each group are labeled a-e and color coded. Subgroup **b** accessions IRIS_313-10314, IRIS_313-10738 and IRIS_313-8554 carry the chromosome 12 resistance associations to group two isolates. Subgroup **c** accessions IRIS_313-10059 and IRIS_313-10879 carry the chromosome 12 resistance associations to group one isolates.

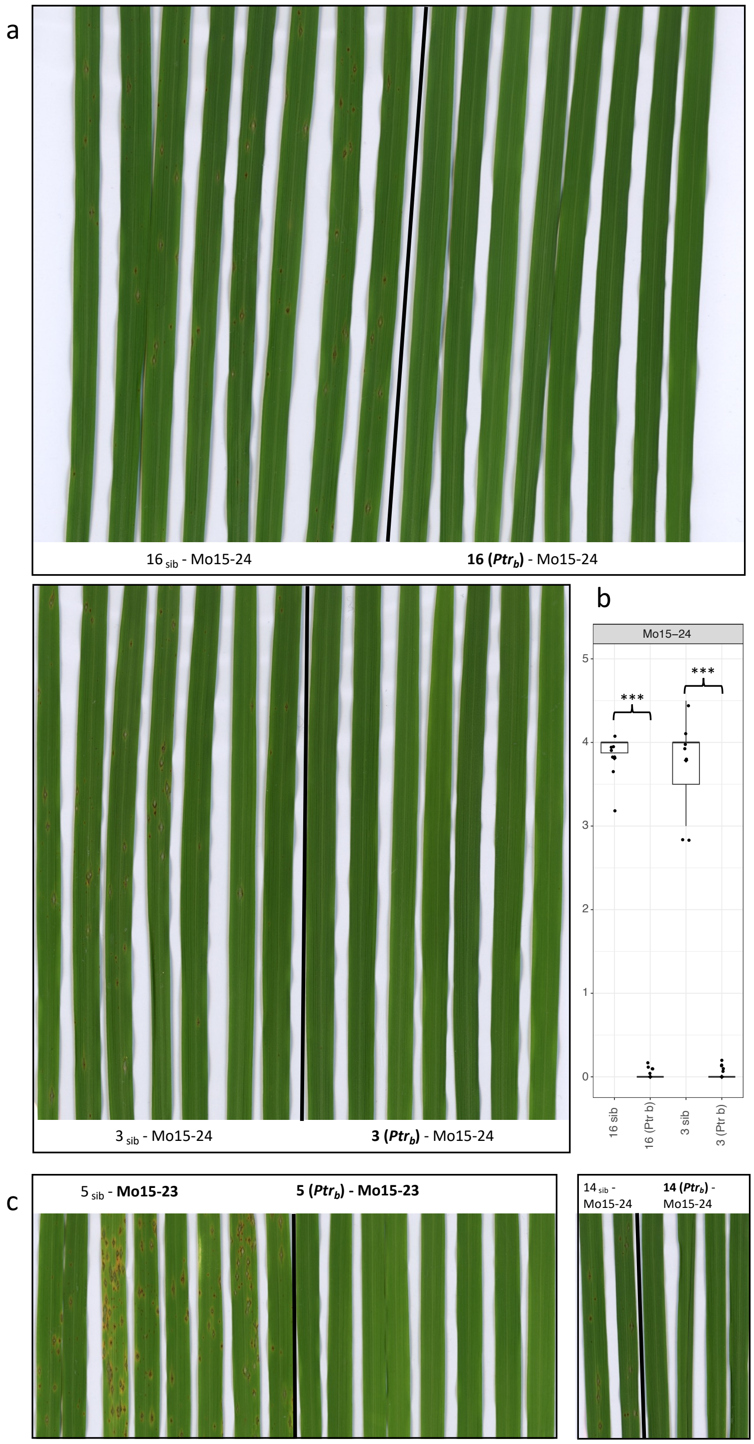

Supplementary Figure 5. Transgenic rice plants expressing the Mo15-23, 24 resistance associated allele of *Ptr* under its native promotor are resistant to rice blast. a,c Rice blast infection of T1 plants genotyped to differentiate non-transgenic segregant siblings (#sib) from plants containing the *Ptrb* transgene (**#*Ptrb***). Four-week-old plants were spray inoculated with *M. oryzae* isolate Mo15-24, except for 5sib* and 5 (***Ptrb***)* plants which were inoculated with Mo15-23 (panel **c**). Representative images of single leaves from individual plants were taken seven days post inoculation. Scoring data is represented by box plots in panel **b** where diamonds indicate the average infection score for each genotype n = 8 (transgenic line 16 plants) and 7 (transgenic line 3 plants). Significant differences, as determined by a two-sample t-test, between sibling and transgenic lines are indicated by an Asterix (*** = P<0.001).

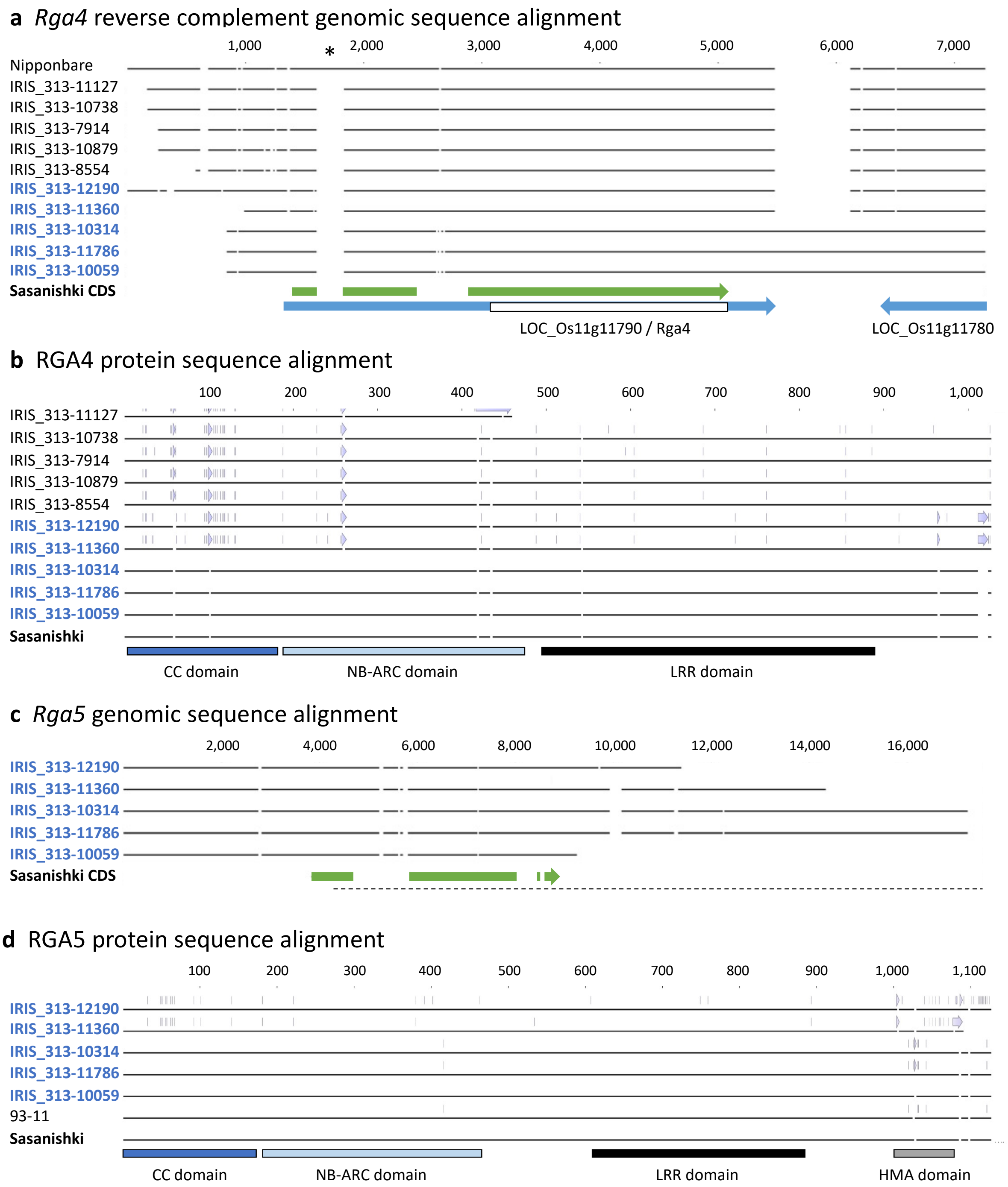

Supplementary Figure 6. ***Pia* locus genomic and protein sequence alignment schematics. a, c** Alignment schematics of *Rga4* and *5* genomic sequence showing positions of in-dels. **a** The Nipponbare genomic sequence features a tandem duplication in *Rga4* sequence resulting in a truncated *Rga4* gene (Position of duplication is marked*). The Nipponbare gene annotation LOC_Os11g11790 which overlaps *RGA4* is shown under the genomic sequence alignment. **a, c** The *RGA4* & *5* CDS from Sasanishki is indicated by green boxes in position of exon sequence. **b, d** RGA4 and 5 protein sequence alignments with positions of AA polymorphisms shown by pale blue vertical lines and AA in-dels shown as pale blue arrows. Positions of conserved protein domains are shown below protein sequence alignments. **a-d** Sequence from rice accessions carrying the C923-49 rice blast resistance association are shown in blue. Protein sequence alignments are included in subsequent supplementary figures.

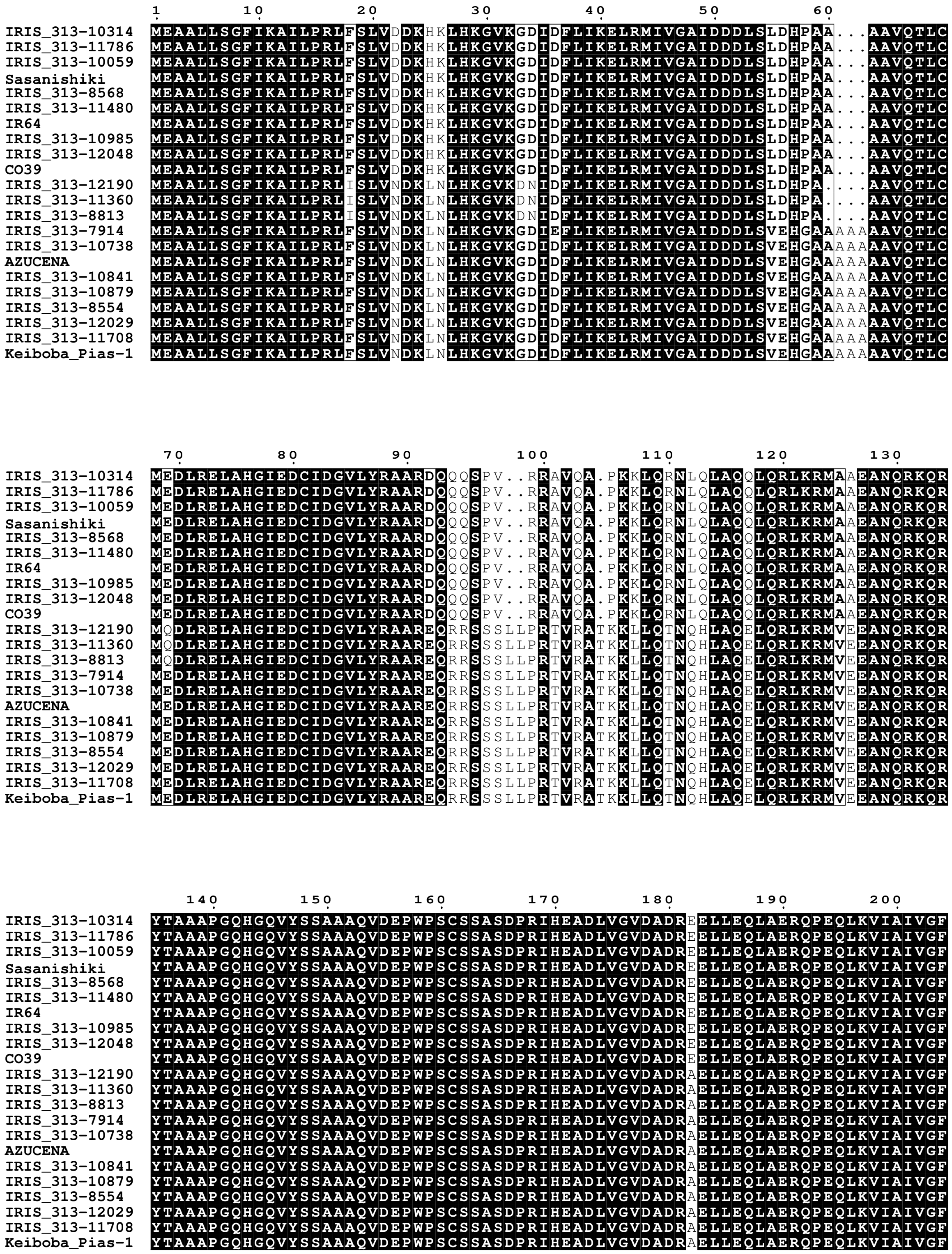
1 10 20 30 40 50 60
IRIS_313-10314 MEAALLSGFIKAILPRLFSLVDDKHKLHKGVKGDIDFLIKELRMIVGAIDDDLSLDHPAA...AAVQTLC
IRIS_313-11786 MEAALLSGFIKAILPRLFSLVDDKHKLHKGVKGDIDFLIKELRMIVGAIDDDLSLDHPAA...AAVQTLC
IRIS_313-10059 MEAALLSGFIKAILPRLFSLVDDKHKLHKGVKGDIDFLIKELRMIVGAIDDDLSLDHPAA...AAVQTLC
Sasanishiki MEAALLSGFIKAILPRLFSLVDDKHKLHKGVKGDIDFLIKELRMIVGAIDDDLSLDHPAA...AAVQTLC
IRIS_313-8568 MEAALLSGFIKAILPRLFSLVDDKHKLHKGVKGDIDFLIKELRMIVGAIDDDLSLDHPAA...AAVQTLC
IRIS_313-11480 MEAALLSGFIKAILPRLFSLVDDKHKLHKGVKGDIDFLIKELRMIVGAIDDDLSLDHPAA...AAVQTLC
IR64 MEAALLSGFIKAILPRLFSLVDDKHKLHKGVKGDIDFLIKELRMIVGAIDDDLSLDHPAA...AAVQTLC
IRIS_313-10985 MEAALLSGFIKAILPRLFSLVDDKHKLHKGVKGDIDFLIKELRMIVGAIDDDLSLDHPAA...AAVQTLC
IRIS_313-12048 MEAALLSGFIKAILPRLFSLVDDKHKLHKGVKGDIDFLIKELRMIVGAIDDDLSLDHPAA...AAVQTLC
CO39 MEAALLSGFIKAILPRLFSLVDDKHKLHKGVKGDIDFLIKELRMIVGAIDDDLSLDHPAA...AAVQTLC
IRIS_313-12190 MEAALLSGFIKAILPRLISLVNDKLNLHKGVKDNIDFLIKELRMIVGAIDDDLSLDHPA....AAVQTLC
IRIS_313-11360 MEAALLSGFIKAILPRLISLVNDKLNLHKGVKDNIDFLIKELRMIVGAIDDDLSLDHPA....AAVQTLC
IRIS_313-8813 MEAALLSGFIKAILPRLISLVNDKLNLHKGVKDNIDFLIKELRMIVGAIDDDLSLDHPA....AAVQTLC
IRIS_313-7914 MEAALLSGFIKAILPRLFSLVNDKLNLHKGVKGDIEFLIKELRMIVGAIDDDLSVEHGAAAAAAAVQTLC
IRIS_313-10738 MEAALLSGFIKAILPRLFSLVNDKLNLHKGVKGDIDFLIKELRMIVGAIDDDLSVEHGAAAAAAAVQTLC
AZUCENA MEAALLSGFIKAILPRLFSLVNDKLNLHKGVKGDIDFLIKELRMIVGAIDDDLSVEHGAAAAAAAVQTLC
IRIS_313-10841 MEAALLSGFIKAILPRLFSLVNDKLNLHKGVKGDIDFLIKELRMIVGAIDDDLSVEHGAAAAAAAVQTLC
IRIS_313-10879 MEAALLSGFIKAILPRLFSLVNDKLNLHKGVKGDIDFLIKELRMIVGAIDDDLSVEHGAAAAAAAVQTLC
IRIS_313-8554 MEAALLSGFIKAILPRLFSLVNDKLNLHKGVKGDIDFLIKELRMIVGAIDDDLSVEHGAAAAAAAVQTLC
IRIS_313-12029 MEAALLSGFIKAILPRLFSLVNDKLNLHKGVKGDIDFLIKELRMIVGAIDDDLSVEHGAAAAAAAVQTLC
IRIS_313-11708 MEAALLSGFIKAILPRLFSLVNDKLNLHKGVKGDIDFLIKELRMIVGAIDDDLSVEHGAAAAAAAVQTLC
Keiboba_Pias-1 MEAALLSGFIKAILPRLFSLVNDKLNLHKGVKGDIDFLIKELRMIVGAIDDDLSVEHGAAAAAAAVQTLC
70 80 90 100 110 120 130
IRIS_313-10314 MEDLRELAHGIEDCIDGVLYRAARDQQQSPV..RRAVQA.PKKLQRNLQLAQQLQRLKRMAAEANQRKQR
IRIS_313-11786 MEDLRELAHGIEDCIDGVLYRAARDQQQSPV..RRAVQA.PKKLQRNLQLAQQLQRLKRMAAEANQRKQR
IRIS_313-10059 MEDLRELAHGIEDCIDGVLYRAARDQQQSPV..RRAVQA.PKKLQRNLQLAQQLQRLKRMAAEANQRKQR
Sasanishiki MEDLRELAHGIEDCIDGVLYRAARDQQQSPV..RRAVQA.PKKLQRNLQLAQQLQRLKRMAAEANQRKQR
IRIS_313-8568 MEDLRELAHGIEDCIDGVLYRAARDQQQSPV..RRAVQA.PKKLQRNLQLAQQLQRLKRMAAEANQRKQR
IRIS_313-11480 MEDLRELAHGIEDCIDGVLYRAARDQQQSPV..RRAVQA.PKKLQRNLQLAQQLQRLKRMAAEANQRKQR
IR64 MEDLRELAHGIEDCIDGVLYRAARDQQQSPV..RRAVQA.PKKLQRNLQLAQQLQRLKRMAAEANQRKQR
IRIS_313-10985 MEDLRELAHGIEDCIDGVLYRAARDQQQSPV..RRAVQA.PKKLQRNLQLAQQLQRLKRMAAEANQRKQR
IRIS_313-12048 MEDLRELAHGIEDCIDGVLYRAARDQQQSPV..RRAVQA.PKKLQRNLQLAQQLQRLKRMAAEANQRKQR
CO39 MEDLRELAHGIEDCIDGVLYRAARDQQQSPV..RRAVQA.PKKLQRNLQLAQQLQRLKRMAAEANQRKQR
IRIS_313-12190 MQDLRELAHGIEDCIDGVLYRAAREQRRSSSLLPRTVRATKKLLQTNQHLAQELQRLKRMVEEANQRKQR
IRIS_313-11360 MQDLRELAHGIEDCIDGVLYRAAREQRRSSSLLPRTVRATKKLLQTNQHLAQELQRLKRMVEEANQRKQR
IRIS_313-8813 MQDLRELAHGIEDCIDGVLYRAAREQRRSSSLLPRTVRATKKLLQTNQHLAQELQRLKRMVEEANQRKQR
IRIS_313-7914 MEDLRELAHGIEDCIDGVLYRAAREQRRSSSLLPRTVRATKKLLQTNQHLAQELQRLKRMVEEANQRKQR
IRIS_313-10738 MEDLRELAHGIEDCIDGVLYRAAREQRRSSSLLPRTVRATKKLLQTNQHLAQELQRLKRMVEEANQRKQR
AZUCENA MEDLRELAHGIEDCIDGVLYRAAREQRRSSSLLPRTVRATKKLLQTNQHLAQELQRLKRMVEEANQRKQR
IRIS_313-10841 MEDLRELAHGIEDCIDGVLYRAAREQRRSSSLLPRTVRATKKLLQTNQHLAQELQRLKRMVEEANQRKQR
IRIS_313-10879 MEDLRELAHGIEDCIDGVLYRAAREQRRSSSLLPRTVRATKKLLQTNQHLAQELQRLKRMVEEANQRKQR
IRIS_313-8554 MEDLRELAHGIEDCIDGVLYRAAREQRRSSSLLPRTVRATKKLLQTNQHLAQELQRLKRMVEEANQRKQR
IRIS_313-12029 MEDLRELAHGIEDCIDGVLYRAAREQRRSSSLLPRTVRATKKLLQTNQHLAQELQRLKRMVEEANQRKQR
IRIS_313-11708 MEDLRELAHGIEDCIDGVLYRAAREQRRSSSLLPRTVRATKKLLQTNQHLAQELQRLKRMVEEANQRKQR
Keiboba_Pias-1 MEDLRELAHGIEDCIDGVLYRAAREQRRSSSLLPRTVRATKKLLQTNQHLAQELQRLKRMVEEANQRKQR
140 150 160 170 180 190 200
IRIS_313-10314 YTAAAPGQHGQVYSSAAAQVDEPWPSCSSASDPRIHEADLVGVDADREELLEQLAERQPEQLKVIAIVGF
IRIS_313-11786 YTAAAPGQHGQVYSSAAAQVDEPWPSCSSASDPRIHEADLVGVDADREELLEQLAERQPEQLKVIAIVGF
IRIS_313-10059 YTAAAPGQHGQVYSSAAAQVDEPWPSCSSASDPRIHEADLVGVDADREELLEQLAERQPEQLKVIAIVGF
Sasanishiki YTAAAPGQHGQVYSSAAAQVDEPWPSCSSASDPRIHEADLVGVDADREELLEQLAERQPEQLKVIAIVGF
IRIS_313-8568 YTAAAPGQHGQVYSSAAAQVDEPWPSCSSASDPRIHEADLVGVDADREELLEQLAERQPEQLKVIAIVGF
IRIS_313-11480 YTAAAPGQHGQVYSSAAAQVDEPWPSCSSASDPRIHEADLVGVDADREELLEQLAERQPEQLKVIAIVGF
IR64 YTAAAPGQHGQVYSSAAAQVDEPWPSCSSASDPRIHEADLVGVDADREELLEQLAERQPEQLKVIAIVGF
IRIS_313-10985 YTAAAPGQHGQVYSSAAAQVDEPWPSCSSASDPRIHEADLVGVDADREELLEQLAERQPEQLKVIAIVGF
IRIS_313-12048 YTAAAPGQHGQVYSSAAAQVDEPWPSCSSASDPRIHEADLVGVDADREELLEQLAERQPEQLKVIAIVGF
CO39 YTAAAPGQHGQVYSSAAAQVDEPWPSCSSASDPRIHEADLVGVDADREELLEQLAERQPEQLKVIAIVGF
IRIS_313-12190 YTAAAPGQHGQVYSSAAAQVDEPWPSCSSASDPRIHEADLVGVDADRAELLEQLAERQPEQLKVIAIVGF
IRIS_313-11360 YTAAAPGQHGQVYSSAAAQVDEPWPSCSSASDPRIHEADLVGVDADRAELLEQLAERQPEQLKVIAIVGF
IRIS_313-8813 YTAAAPGQHGQVYSSAAAQVDEPWPSCSSASDPRIHEADLVGVDADRAELLEQLAERQPEQLKVIAIVGF
IRIS_313-7914 YTAAAPGQHGQVYSSAAAQVDEPWPSCSSASDPRIHEADLVGVDADRAELLEQLAERQPEQLKVIAIVGF
IRIS_313-10738 YTAAAPGQHGQVYSSAAAQVDEPWPSCSSASDPRIHEADLVGVDADRAELLEQLAERQPEQLKVIAIVGF
AZUCENA YTAAAPGQHGQVYSSAAAQVDEPWPSCSSASDPRIHEADLVGVDADRAELLEQLAERQPEQLKVIAIVGF
IRIS_313-10841 YTAAAPGQHGQVYSSAAAQVDEPWPSCSSASDPRIHEADLVGVDADRAELLEQLAERQPEQLKVIAIVGF
IRIS_313-10879 YTAAAPGQHGQVYSSAAAQVDEPWPSCSSASDPRIHEADLVGVDADRAELLEQLAERQPEQLKVIAIVGF
IRIS_313-8554 YTAAAPGQHGQVYSSAAAQVDEPWPSCSSASDPRIHEADLVGVDADRAELLEQLAERQPEQLKVIAIVGF
IRIS_313-12029 YTAAAPGQHGQVYSSAAAQVDEPWPSCSSASDPRIHEADLVGVDADRAELLEQLAERQPEQLKVIAIVGF
IRIS_313-11708 YTAAAPGQHGQVYSSAAAQVDEPWPSCSSASDPRIHEADLVGVDADRAELLEQLAERQPEQLKVIAIVGF
Keiboba_Pias-1 YTAAAPGQHGQVYSSAAAQVDEPWPSCSSASDPRIHEADLVGVDADRAELLEQLAERQPEQLKVIAIVGF

```
                        210       220       230       240       250       260       270
IRIS_313-10314   CGLGKTALAAEAYNRETGGGRFERHAWVCAGHRSAREVLGELLRRLDADGRSFHGDSDAGQLCVDIRQQL
IRIS_313-11786   CGLGKTALAAEAYNRETGGGRFERHAWVCAGHRSAREVLGELLRRLDADGRSFHGDSDAGQLCVDIRQQL
IRIS_313-10059   CGLGKTALAAEAYNRETGGGRFERHAWVCAGHRSAREVLGELLRRLDADGRSFHGDSDAGQLCVDIRQQL
Sasanishiki      CGLGKTALAAEAYNRETGGGRFERHAWVCAGHRSAREVLGELLRRLDADGRSFHGDSDAGQLCVDIRQQL
IRIS_313-8568    CGLGKTALAAEAYNRETGGGRFERHAWVCAGHRSAREVLGELLRRLDADGRSFHGDSDAGQLCVDIRQQL
IRIS_313-11480   CGLGKTALAAEAYNRETGGGRFERHAWVCAGHRSAREVLGELLRRLDADGRSFHGDSDAGQLCVDIRQQL
IR64             CGLGKTALAAEAYNRETGGGRFERHAWVCAGHRSAREVLGELLRRLDADGRSFHGDSDAGQLCVDIRQQL
IRIS_313-10985   CGLGKTALAAEAYNRETGGGRFERHAWVCAGHRSAREVLGELLRRLDADGRSFHGDSDAGQLCVDIRQQL
IRIS_313-12048   CGLGKTALAAEAYNRETGGGRFERHAWVCAGHRSAREVLGELLRRLDADGRSFHGDSDAGQLCVDIRQQL
CO39             CGLGKTALAAEAYNRETGGGRFERHAWVCAGHRSAREVLGELLRRLDADGRSFHGDSDAGQLCVDIRQQL
IRIS_313-12190   CGLGKTALAAEAYNRETRGGRFERHAWVCAAHRSAREVLGELLRRIDA...ACHGDSDAGQLCVDIRQQL
IRIS_313-11360   CGLGKTALAAEAYNRETRGGRFERHAWVCAAHRSAREVLGELLRRIDA...ACHGDSDAGQLCVDIRQQL
IRIS_313-8813    CGLGKTALAAEAYNRETRGGRFERHAWVCAAHRSAREVLGELLRRIDA...ACHGDSDAGQLCVDIRQQL
IRIS_313-7914    CGLGKTALAAEAYNRETHGGRFERHAWVCAAHRSAREVLGELLRRIDA...ACHGDSDAGQLCVDIRQQL
IRIS_313-10738   CGLGKTALAAEAYNRETRGGRFERHAWVCAAHRSAREVLGELLRRIDA...ACHGDSDAGQLCVDIRQQL
AZUCENA          CGLGKTALAAEAYNRETRGGRFERHAWVCAAHRSAREVLGELLRRIDA...ACHGDSDAGQLCVDIRQQL
IRIS_313-10841   CGLGKTALAAEAYNRETRGGRFERHAWVCAAHRSAREVLGELLRRIDA...ACHGDSDAGQLCVDIRQQL
IRIS_313-10879   CGLGKTALAAEAYNRETRGGRFERHAWVCAAHRSAREVLGELLRRIDA...ACHGDSDAGQLCVDIRQQL
IRIS_313-8554    CGLGKTALAAEAYNRETRGGRFERHAWVCAAHRSAREVLGELLRRIDA...ACHGDSDAGQLCVDIRQQL
IRIS_313-12029   CGLGKTALAAEAYNRETRGGRFERHAWVCAAHRSAREVLGELLRRIDA...ACHGDSDAGQLCVDIRQQL
IRIS_313-11708   CGLGKTALAAEAYNRETRGGRFERHAWVCAAHRSAREVLGELLRRIDA...ACHGDSDAGQLCVDIRQQL
Keiboba_Pias-1   CGLGKTALAAEAYNRETRGGRFERHAWVCAAHRSAREVLGELLRRIDA...ACHGDSDAGQLCVDIRQQL
```

```
                        280       290       300       310       320       330       340
IRIS_313-10314   EKNRYFIVIDDIQTEDQWKSIKSAFPTDKDIGSRIVVTTTIQSVANACCSANGYLHKMSRLDKNCSKQLL
IRIS_313-11786   EKNRYFIVIDDIQTEDQWKSIKSAFPTDKDIGSRIVVTTTIQSVANACCSANGYLHKMSRLDKNCSKQLL
IRIS_313-10059   EKNRYFIVIDDIQTEDQWKSIKSAFPTDKDIGSRIVVTTTIQSVANACCSANGYLHKMSRLDKNCSKQLL
Sasanishiki      EKNRYFIVIDDIQTEDQWKSIKSAFPTDKDIGSRIVVTTTIQSVANACCSANGYLHKMSRLDKNCSKQLL
IRIS_313-8568    EKNRYFIVIDDIQTEDQWKSIKSAFPTDKDIGSRIVVTTTIQSVANACCSANGYLHKMSRLDKNCSKQLL
IRIS_313-11480   EKNRYFIVIDDIQTEDQWKSIKSAFPTDKDIGSRIVVTTTIQSVANACCSANGYLHKMSRLDKNCSKQLL
IR64             EKNRYFIVIDDIQTEDQWKSIKSAFPTDKDIGSRIVVTTTIQSVANACCSANGYLHKMSRLDKNCSKQLL
IRIS_313-10985   EKNRYFIVIDDIQTEDQWKSINSAFPTDKDIGSRIVVTTTIQSVANACCSANGYLHKMSRLDKNCSKQLL
IRIS_313-12048   EKNRYFIVIDDIQTEDQWKSIKSAFPTDKDIGSRIVVTTTIQSVANACCSANGYLHKMSRLDKNCSKQLL
CO39             EKNRYFIVIDDIQTEDQWKSIKSAFPTDKDIGSRIVVTTTIQSVANACCSANGYLHKMSRLDKNCSKQLL
IRIS_313-12190   EKNRYFIVIDDIQTEDQWKSIKSAFPTDKDIGSRIVVTTTIQSVANACCSANGYLHKMSRLDKNCSKQLL
IRIS_313-11360   EKNRYFIVIDDIQTEDQWKSIKSAFPTDKDIGSRIVVTTTIQSVANACCSANGYLHKMSRLDKNCSKQLL
IRIS_313-8813    EKNRYFIVIDDIQTEDQWKSIKSAFPTDKDIGSRIVVTTTIQSVANACCSANGYLHKMSRLDKNCSKQLL
IRIS_313-7914    EKNRYFIVIDDIQTEDQWKSIKSAFPTDKDIGSRIVVTTTIQSVANACCSANGYLHKMSRLDKNCSKQLL
IRIS_313-10738   EKNRYFIVIDDIQTEDQWKSIKSAFPTDKDIGSRIVVTTTIQSVANACCSANGYLHKMSRLDKNCSKQLL
AZUCENA          EKNRYFIVIDDIQTEDQWKSIKSAFPTDKDIGSRIVVTTTIQSVANACCSANGYLHKMSRLDKNCSKQLL
IRIS_313-10841   EKNRYFIVIDDIQTEDQWKSIKSAFPTDKDIGSRIVVTTTIQSVANACCSANGYLHKMSRLDKNCSKQLL
IRIS_313-10879   EKNRYFIVIDDIQTEDQWKSIKSAFPTDKDIGSRIVVTTTIQSVANACCSANGYLHKMSRLDKNCSKQLL
IRIS_313-8554    EKNRYFIVIDDIQTEDQWKSIKSAFPTDKDIGSRIVVTTTIQSVANACCSANGYLHKMSRLDKNCSKQLL
IRIS_313-12029   EKNRYFIVIDDIQTEDQWKSIKSAFPTDKDIGSRIVVTTTIQSVANACCSANGYLHKMSRLDKNCSKQLL
IRIS_313-11708   EKNRYFIVIDDIQTEDQWKSIKSAFPTDKDIGSRIVVTTTIQSVANACCSANGYLHKMSRLDKNCSKQLL
Keiboba_Pias-1   EKKRYFIVIDDIQTEDQWKSIKSAFPTDKDIGSRIVVTTTIQSVANACCSANGYLHKMSRLDKNCSKQLL
```

```
                        350       360       370       380       390       400       410
IRIS_313-10314   SKKACPERYSHYKQPDSAAILKKCDGQPLALVTIGEFLQANGWPTGPNCEDLCNRLHYHLENDKTLERMW
IRIS_313-11786   SKKACPERYSHYKQPDSAAILKKCDGQPLALVTIGEFLQANGWPTGPNCEDLCNRLHYHLENDKTLERMW
IRIS_313-10059   SKKACPERYSHYKQPDSAAILKKCDGQPLALVTIGEFLQANGWPTGPNCEDLCNRLHYHLENDKTLERMW
Sasanishiki      SKKACPERYSHYKQPDSAAILKKCDGQPLALVTIGEFLQANGWPTGPNCEDLCNRLHYHLENDKTLERMW
IRIS_313-8568    SKKACPERYSHYKQPDSAAILKKCDGQPLALVTIGEFLQANGWPTGPNCEDLCNRLHYHLENDKTLERMW
IRIS_313-11480   SKKACPERYSHYKQPDSAAILKKCDGQPLALVTIGEFLQANGWPTGPNCEDLCNRLHYHLENDKTLERMW
IR64             SKKACPERYSHYKQPDSAAILKKCDGQPLALVTIGEFLQANGWPTGPNCEDLCNRLHYHLENDKTLERMW
IRIS_313-10985   SKKACPERYSHYKQPDSAAILKKCDGQPLALVTIGEFLQANGWPTGPNCEDLCNRLHYHLENDKTLERMW
IRIS_313-12048   SKKACPERYSHYKQPDSAAILKKCDGQPLALVTIGEFLQANGWPTGPNCEDLCNRLHYHLENDKTLERMW
CO39             SKKACPERYSHYKQPDSAAILKKCDGQPLALVTIGEFLQANGWPMGPNCEDLCNRLHYHLENDKTLERMW
IRIS_313-12190   SKKACPERYSHYKQPDSAAILKKCDGQPLALVTIGEFLQANGWPTGPNCEDLCNRLHYHLENDKTLERMR
IRIS_313-11360   SKKACPERYSHYKQPDSAAILKKCDGQPLALVTIGEFLQANGWPTGPNCEDLCNRLHYHLENDKTLERMR
IRIS_313-8813    SKKACPERYSHYKQPDSAAILKKCDGQPLALVTIGEFLQANGWPTGPNCEDLCNRLHYHLENDKTLERMR
IRIS_313-7914    SKKACPERYSHYKQPDSAAILKKCDGQPLALVTIGEFLQANGWPTGPNCEDLCNRLHYHLENDKTLERMR
IRIS_313-10738   SKKACPERYSHYKQPDSAAILKKCDGQPLALVTIGEFLQANGWPTGPNCEDLCNRLHYHLENDKTLERMR
AZUCENA          SKKACPERYSHYKQPDSAAILKKCDGQPLALVTIGEFLQANGWPTGPNCEDLCNRLHYHLENDKTLERMR
IRIS_313-10841   SKKACPERYSHYKQPDSAAILKKCDGQPLALVTIGEFLQANGWPTGPNCEDLCNRLHYHLENDKTLERMR
IRIS_313-10879   SKKACPERYSHYKQPDSAAILKKCDGQPLALVTIGEFLQANGWPTGPNCEDLCNRLHYHLENDKTLERMR
IRIS_313-8554    SKKACPERYSHYKQPDSAAILKKCDGQPLALVTIGEFLQANGWPTGPNCEDLCNRLHYHLENDKTLERMR
IRIS_313-12029   SKKACPERYSHYKQPDSAAILKKCDGQPLALVTIGEFLQANGWPTGPNCEDLCNRLHYHLENDKTLERMR
IRIS_313-11708   SKKACPERYSHYKQPDSAAILKKCDGQPLALVTIGEFLQANGWPTGPNCEDLCNRLHYHLENDKTLERMR
Keiboba_Pias-1   SKKACPERYSHYKQPDSAAILKKCDGQPLALVTIGEFLQANGWPTGPNCEDLCNRLHYHLENDKTLERMR
```

```
                       420       430       440       450       460       470       480
IRIS_313-10314   RVLVRNYTSLPGHALKACLLYFGMFPSDHPIRRKSLLRRWLAEGFVEPLSSSSNIDSTAAFNVLMDRNII
IRIS_313-11786   RVLVRNYTSLPGHALKACLLYFGMFPSDHPIRRKSLLRRWLAEGFVEPLSSSSNIDSTAAFNVLMDRNII
IRIS_313-10059   RVLVRNYTSLPGHALKACLLYFGMFPSDHPIRRKSLLRRWLAEGFVEPLSSSSNIDSTAAFNVLMDRNII
Sasanishiki      RVLVRNYTSLPGHALKACLLYFGMFPSDHPIRRKSLLRRWLAEGFVEPLSSSSNIDSTAAFNVLMDRNII
IRIS_313-8568    RVLVRNYTSLPGHALKACLLYFGMFPSDHPIRRKSLLRRWLAEGFVEPLSSSSNIDSTAAFNVLMDRNII
IRIS_313-11480   RVLVRNYTSLPGHALKACLLYFGMFPSDHPIRRKSLLRRWLAEGFVEPLSSSSNIDSTAAFNVLMDRNII
IR64             RVLVRNYTSLPGHALKACLLYFGMFPSDHPIRRKSLLRRWLAEGFVEPLSSSSNIDSTAAFNVLMDRNII
IRIS_313-10985   RVLVRNYTSLPGHALKACLLYFGMFPSDHPIRRKSLLRRWLAEGFVEPLSSSSNIDSTAAFNVLMDRNII
IRIS_313-12048   RVLVRNYTSLPGHALKACLLYFGMFPSDHPIRRKSLLRRWLAEGFVEPLSSSSNIDSTAAFNVLMDRNII
CO39             RVLVRNYTSLPGHALKACLLYFGMFPSDHPIRRKSLLRRWLAEGFVEPLSSSSNIDSTAAFNVLMDRNII
IRIS_313-12190   RVLVRNYTSLPGHALKACLLYFGMFPSDHPIRRKSLLRRWLAEGFVEPVSSSSNLDSTAAFDVLMDRNII
IRIS_313-11360   RLLVRNYTSLPGHALKACLLYFGMFPSDHPIRRKSLLRRWLAEGFVEPVSSSSNLDSTAAFDVLMDRNII
IRIS_313-8813    RVLVRNYTSLPGHALKACLLYFGMFPSDHPIRRKSLLRRWLAEGFVEPVSSSSNLDSTAAFDVLMDRNII
IRIS_313-7914    RVLVRNYTSLPGHALKACLLYFGMFPSDHPIRRKSLLRRWLAEGFVEPVSSSSNLDSTAAFDVLMDRNII
IRIS_313-10738   RVLVRNYTSLPGHALKACLLYFGMFPSDHPIRRKSLLRRWLAEGFVEPVSSSSNLDSTAAFDVLMDRNII
AZUCENA          RVLVRNYTSLPGHALKACLLYFGMFPSDHPIRRKSLLRRWLAEGFVEPVSSSSNLDSTAAFDVLMDRNII
IRIS_313-10841   RVLVRNYTSLPGHALKACLLYFGMFPSDHPIRRKSLLRRWLAEGFVEPVSSSSNLDSTAAFDVLMDRNII
IRIS_313-10879   RVLVRNYTSLPGHALKACLLYFGMFPSDHPIRRKSLLRRWLAEGFVEPVSSSSNLDSTAAFDVLMDRNII
IRIS_313-8554    RVLVRNYTSLPGHALKACLLYFGMFPSDHPIRRKSLLRRWLAEGFVEPVSSSSNLDSTAAFDVLMDRNII
IRIS_313-12029   RVLVRNYTSLPGHALKACLLYFGMFPSDHPIRRKSLLRRWLAEGFVEPVSSSSNLDSTAAFDVLMDRNII
IRIS_313-11708   RVLVRNYTSLPGHALKACLLYFGMFPSDHPIRRKSLLRRWLAEGFVEPVSSSSNLDSTAAFDVLMDRNII
Keiboba_Pias-1   RVLVRNYTSLPGHALKACLLYFGMFPSDHPIRRKSLLRRWLAEGFVEPVSSSSNLDSTAAFDVLMDRNII
```

```
                       490       500       510       520       530       540       550
IRIS_313-10314   EPINVSNNDKVKTCQTYGMMREFISHMSISQNFVTFFCDDKFVPKYVRRLSLHGDTVVNGDNFNGIDLSL
IRIS_313-11786   EPINVSNNDKVKTCQTYGMMREFISHMSISQNFVTFFCDDKFVPKYVRRLSLHGDTVVNGDNFNGIDLSL
IRIS_313-10059   EPINVSNNDKVKTCQTYGMMREFISHMSISQNFVTFFCDDKFVPKYVRRLSLHGDTVVNGDNFNGIDLSL
Sasanishiki      EPINVSNNDKVKTCQTYGMMREFISHMSISQNFVTFFCDDKFVPKYVRRLSLHGDTVVNGDNFNGIDLSL
IRIS_313-8568    EPINVSNNDKVKTCQTYGMMREFISHMSISQNFVTFFCDDKFVPKYVRRLSLHGDTVVNGDNFNGIDLSL
IRIS_313-11480   EPINVSNNDKVKTCQTYGMMREFISHMSISQNFVTFFCDDKFVPKYVRRLSLHGDTVVNGDNFNGIDLSL
IR64             EPINVSNNDKVKTCQTYGMMREFISHMSISQNFVTFFCDDKFVPKYVRRLSLHGDTVVNGDNFNGIDLSL
IRIS_313-10985   EPINVSNNDKVKTCQTYGMMREFISHMSISQNFVTFFCDDKFVPKYVRRLSLHGDTVVNGDNFNGIDLSL
IRIS_313-12048   EPINVSNNDKVKTCQTYGMMREFISHMSISQNFVTFFCDDKFVPKYVRRLSLHGDTVVNGDNFNGIDLSL
CO39             EPINVSNNDKVKTCQTYGMMREFISHMSISQNFVTFFCDDKFVPKYVRRLSLHGDTVVNGDNFNGIDLSL
IRIS_313-12190   EPINVSNNDKVKTCQMYGMMREFISHMSISQNFVTFFCDDKFLPKYVRRLSLHGDTVVNGDNFNGIDLSL
IRIS_313-11360   EPINVSNNDKVKTCQMYGMMREFISHMSISQNFVTFFCDDKFLPKYVRRLSLHGDTVVNGDNFNGIDLSL
IRIS_313-8813    EPINVSNNDKVKTCQTYGMMREFISHMSISQNFVTFFCDDKFLPKYVRRLSLHGDTVVNGDNFNGIDLSL
IRIS_313-7914    EPINVSNNDKVKTCQTYGMMREFISHMSISQNFVTFFCDDKFLPKYVRRLSLHGDTVVNGDNFNGIDLSL
IRIS_313-10738   EPINVSNNDKVKTCQTYGMMREFISHMSISQNFVTFFCDDKFLPKYVRRLSLHGDTVVNGDNFNGIDLSL
AZUCENA          EPINVSNNDKVKTCQTYGMMREFISHMSISQNFVTFFCDDKFLPKYVRRLSLHGDTVVNGDNFNGIDLSL
IRIS_313-10841   EPINVSNNDKVKTCQTYGMMREFISHMSISQNFVTFFCDDKFLPKYVRRLSLHGDTVVNGDNFNGIDLSL
IRIS_313-10879   EPINVSNNDKVKTCQTYGMMREFISHMSISQNFVTFFCDDKFLPKYVRRLSLHGDTVVNGDNFNGIDLSL
IRIS_313-8554    EPINVSNNDKVKTCQTYGMMREFISHMSISQNFVTFFCDDKFLPKYVRRLSLHGDTVVNGDNFNGIDLSL
IRIS_313-12029   EPINVSNNDKVKTCQTYGMMREFISHMSISQNFVTFFCDDKFLPKYVRRLSLHGDTVVNGDNFNGIDLSL
IRIS_313-11708   EPINVSNNDKVKTCQTYGMMREFISHMSISQNFVTFFCDDKFLPKYVRRLSLHGDTVVNGDNFNGIDLSL
Keiboba_Pias-1   EPINVSNNDKVKTCQTYGMMREFISHMSISQNFVTFFCDDKFLPKYVRRLSLHGDTVVNGDNFNGIDLSL
                                 MHD
                                 motif
```

```
                       560       570       580       590       600       610       620
IRIS_313-10314   VRSLAVFGEAGTTVLDFSKYQLLRVLDLEKCDDLKDDHLKEICNLVLLKYLSLGGNISKLPKDIAKLKDL
IRIS_313-11786   VRSLAVFGEAGTTVLDFSKYQLLRVLDLEKCDDLKDDHLKEICNLVLLKYLSLGGNISKLPKDIAKLKDL
IRIS_313-10059   VRSLAVFGEAGTTVLDFSKYQLLRVLDLEKCDDLKDDHLKEICNLVLLKYLSLGGNISKLPKDIAKLKDL
Sasanishiki      VRSLAVFGEAGTTVLDFSKYQLLRVLDLEKCDDLKDDHLKEICNLVLLKYLSLGGNISKLPKDIAKLKDL
IRIS_313-8568    VRSLAVFGEAGTTVLDFSKYQLLRVLDLEKCDDLKDDHLKEICNLVLLKYLSLGGNISKLPKDIAKLKDL
IRIS_313-11480   VRSLAVFGEAGTTVLDFSKYQLLRVLDLEKCDDLKDDHLKEICNLVLLKYLSLGGNISKLPKDIAKLKDL
IR64             VRSLAVFGEAGTTVLDFSKYQLLRVLDLEKCDDLKDDHLKEICNLVLLKYLSLGGNISKLPKDIAKLKDL
IRIS_313-10985   VRSLAVFGEAGTTVLDFSKYQLLRVLDLEKCDDLKDDHLKEICNLVLLKYLSLGGNISKLPKDIAKLKDL
IRIS_313-12048   VRSLAVFGEAGTTVLDFSKYQLLRVLDLEKCDDLKDDHLKEICNLVLLKYLSLGGNISKLPKDIAKLKDL
CO39             VRSLAVFGEAGTTVLDFSKYQLLRVLDLEKCDDLKDDHLKEICNLVLLKYLSLGGNISKLPKDIAKLKDL
IRIS_313-12190   VRSLAVFGEAGTTVLDFSKYQLLRVLDLEKCDDLNDDHLKEICNLVLLKYLSLGGNISKLPKDIAKLKDL
IRIS_313-11360   VRSLAVFGEAGTTVLDFSKYQLLRVLDLEKCDDLNDDHLKEICNLVLLKYLSLGGNISKLPKDIAKLKDL
IRIS_313-8813    VRSLAVFGEAGTTVLDFSKYQLLRVLDLEKCDDLNDDHLKEICNLVLLKYLSLGGNISKLPKDIAKLKDL
IRIS_313-7914    VRSLAVFGEAGTTVLDFSKYQLLRILDLEKCDDLNDDHLKEICNLVLLKYLSLGGNISKLPKDIAKLKDL
IRIS_313-10738   VRSLVVFGEAGTTVLDFSKYQLLRVLDLEKCDDLNDDHLKEICNLVLLKYLSLGGNISKLPKDIAKLKDL
AZUCENA          VRSLVVFGEAGTTVLDFSKYQLLRVLDLEKCDDLNDDHLKEICNLVLLKYLSLGGNISKLPKDIAKLKDL
IRIS_313-10841   VRSLVVFGEAGTTVLDFSKYQLLRVLDLEKCDDLNDDHLKEICNLVLLKYLSLGGNISKLPKDIAKLKDL
IRIS_313-10879   VRSLAVFGEAGTTVLDFSKYQLLRVLDLEKCDDLNDDHLKEICNLVLLKYLSLGGNISKLPKDIAKLKDL
IRIS_313-8554    VRSLAVFGEAGTTVLDFSKYQLLRVLDLEKCDDLNDDHLKEICNLVLLKYLSLGGNISKLPKDIAKLKDL
IRIS_313-12029   VRSLAVFGEAGTTVLDFSKYQLLRVLDLEKCDDLNDDHLKEICNLVLLKYLSLGGNISKLPKDIAKLKDL
IRIS_313-11708   VRSLAVFGEAGTTVLDFSKYQLLRVLDLEKCDDLNDDHLKEICNLVLLKYLSLGGNISKLPKDIAKLKDL
Keiboba_Pias-1   VRSLAVFGEAGTTVLDFSKYQLLRVLDLEKCDDLNDDHLKEICNLVLLKYLSLGGNISKLPKDIAKLKDL
```

```
                     630       640       650       660       670       680       690
IRIS_313-10314  EALDVRRSKVKIMPVEVFGLPCLIHLLGKFKLSDKVKQKTEVQEFLLKGKSNLQTLAGFASNGSEGFLHL
IRIS_313-11786  EALDVRRSKVKIMPVEVFGLPCLIHLLGKFKLSDKVKQKTEVQEFLLKGKSNLQTLAGFASNGSEGFLHL
IRIS_313-10059  EALDVRRSKVKIMPVEVFGLPCLIHLLGKFKLSDKVKQKTEVQEFLLKGKSNLQTLAGFASNGSEGFLHL
Sasanishiki     EALDVRRSKVKIMPVEVFGLPCLIHLLGKFKLSDKVKQKTEVQEFLLKGKSNLQTLAGFASNGSEGFLHL
IRIS_313-8568   EALDVRRSKVKIMPVEVFGLPCLIHLLGKFKLSDKVKQKTEVQEFLLKGKSNLQTLAGFASNGSEGFLHL
IRIS_313-11480  EALDVRRSKVKIMPVEVFGLPCLIHLLGKFKLSDKVKQKTEVQEFLLKGKSNLQTLAGFASNGSEGFLHL
IR64            EALDVRRSKVKIMPVEVFGLPCLIHLLGKFKLSDKVKQKTEVQEFLLKGKSNLQTLAGFASNGSEGFLHL
IRIS_313-10985  EALDVRRSKVKIMPVEVFGLPCLIHLLGKFKLSDKVKQKTEVQEFLLKGKSNLQTLAGFASNGSEGFLHL
IRIS_313-12048  EALDVRRSKVKIMPVEVFGLPCLIHLLGKFKLSDKVKQKTEVQEFLLKGKSNLQTLAGFASNGSEGFLHL
CO39            EALDVRRSKVKIMPVEVFGLPCLIHLLGKFKLSDKVKQKTEVQEFLLKGKSNLQTLAGFASNGSEGFLHL
IRIS_313-12190  EALDVRRSKVKIMPVEVFGLPCLIHLLGKFKLSDKVKQKTEVQEFLLKGKSNLQTLAGFASNGSEGFLHL
IRIS_313-11360  EALDVRRSKVKIMPVEVFGLPCLIHLLGKFKLSDKVKQKTEVQEFLLKGKSNLQTLAGFASNGSEGFLHL
IRIS_313-8813   EALDVRRSKVKIMPVEVFGLPCLIHLLGKFKLSDKVKQKTEVQEFLLKGKSNLQTLAGFASNGSEGFLHL
IRIS_313-7914   EALDVRRSKVKIMPVEVFGLPCLIHLLGKFKLSDKVKQKTEVQEFLSKGKSNLQTLAGFASNGSEGFLHL
IRIS_313-10738  EALDVRRSKVKIMPVEVFGLPCLIHLLGKFKLSDKVKQKTEVQEFLSKGKSNLQTLAGFASNGSEGFLHL
AZUCENA         EALDVRRSKVKIMPVEVFGLPCLIHLLGKFKLSDKVKQKTEVQEFLSKGKSNLQTLAGFASNGSEGFLHL
IRIS_313-10841  EALDVRRSKVKIMPVEVFGLPCLIHLLGKFKLSDKVKQKTEVQEFLSKGKSNLQTLAGFASNGSEGFLHL
IRIS_313-10879  EALDVRRSKVKIMPVEVFGLPCLIHLLGKFKLSDKVKQKTEVQEFLSKGKSNLQTLAGFASNGSEGFLHL
IRIS_313-8554   EALDVRRSKVKIMPVEVFGLPCLIHLLGKFKLSDKVKQKTEVQEFLSKGKSNLQTLAGFASNGSEGFLHL
IRIS_313-12029  EALDVRRSKVKIMPVEVFGLPCLIHLLGKFKLSDKVKQKTEVQEFLSKGKSNLQTLAGFASNGSEGFLHL
IRIS_313-11708  EALDVRRSKVKIMPVEVFGLPCLIHLLGKFKLSDKVKQKTEVQEFLSKGKSNLQTLAGFASNGSEGFLHL
Keiboba_Pias-1  EALDVRRSKVKIMPVEVFGLPCLIHLLGKFKLSDKVKQKTEVQEFLLKGKSNLQTLAGFASNGSEGFLHL
```

```
                     700       710       720       730       740       750       760
IRIS_313-10314  MRYMNKLRKLKIWCTSSAGSTDWTDLREAIQQFILDEKEANIGTRSLSLHFSGCSEDAINSLKEPCYLSS
IRIS_313-11786  MRYMNKLRKLKIWCTSSAGSTDWTDLREAIQQFILDEKEANIGTRSLSLHFSGCSEDAINSLKEPCYLSS
IRIS_313-10059  MRYMNKLRKLKIWCTSSAGSTDWTDLREAIQQFILDEKEANIGTRSLSLHFSGCSEDAINSLKEPCYLSS
Sasanishiki     MRYMNKLRKLKIWCTSSAGSTDWTDLREAIQQFILDEKEANIGTRSLSLHFSGCSEDAINSLKEPCYLSS
IRIS_313-8568   MRYMNKLRKLKIWCTSSAGSTDWTDLREAIQQFILDEKEANIGTRSLSLHFSGCSEDAINSLKEPCYLSS
IRIS_313-11480  MRYMNKLRKLKIWCTSSAGSTDWTDLREAIQQFILDEKEANIGTRSLSLHFSGCSEDAINSLKEPCYLSS
IR64            MRYMNKLRKLKIWCTSSAGSTDWTDLREAIQQFILDEKEANIGTRSLSLHFSGCSEDAINSLKEPCYLSS
IRIS_313-10985  MRYMNKLRKLKIWCTSSAGSTDWTDLREAIQQFILDEKEANIGTRSLSLHFSGCSEDAINSLKEPCYLSS
IRIS_313-12048  MRYMNKLRKLKIWCTSSAGSTDWTDLREAIQQFILDEKEANIGTRSLSLHFSGCSEDAINSLKEPCYLSS
CO39            MRYMNKLRKLKIWCTSSAGSTDWTDLREAIQQFILDEKEANIGTRSLSLHFSGCSEDAINSLKEPCYLSS
IRIS_313-12190  MRYMNKLRKLKIWCMSSAGSTDWTDLREAIQQFILDEKEANIGTRSLSLHFTGCSEDAINSLKEPCYLSS
IRIS_313-11360  MRYMNKLRKLKIWCMSSAGSTDWTDLREAIQQFILDEKEANIGTRSLSLHFTGCSEDAINSLKEPCYLSS
IRIS_313-8813   MRYMNKLRKLKIWCMSSAGSTDWTDLREAIQQFILDEKEANIGTRSLSLHFTGCSEDAINSLKEPCYLSS
IRIS_313-7914   MRYMNKLRKLKIWCTSSAGSTDWTDLREAIQQFILDEKEANIGTRSLSLHFTGCSEDAINSLKEPCYLSS
IRIS_313-10738  MRYMNKLRKLKIWCTSSAGSTDWTDLREAIQQFILDEKEANIGTRSLSLHFTGCSEDAINSLKEPCYLSS
AZUCENA         MRYMNKLRKLKIWCTSSAGSTDWTDLREAIQQFILDEKEANIGTRSLSLHFTGCSEDAINSLKEPCYLSS
IRIS_313-10841  MRYMNKLRKLKIWCTSSAGSTDWTDLREAIQQFILDEKEANIGTRSLSLHFTGCSEDAINSLKEPCYLSS
IRIS_313-10879  MRYMNKLRKLKIWCTSSAGSTDWTDLREAIQQFILDEKEANIGTRSLSLHFTGCSEDAINSLKEPCYLSS
IRIS_313-8554   MRYMNKLRKLKIWCTSSAGSTDWTDLREAIQQFILDEKEANIGTRSLSLHFTGCSEDAINSLKEPCYLSS
IRIS_313-12029  MRYMNKLRKLKIWCTSSAGSTDWTDLREAIQQFILDEKEANIGTRSLSLHFTGCSEDAINSLKEPCYLSS
IRIS_313-11708  MRYMNKLRKLKIWCTSSAGSTDWTDLREAIQQFILDEKEANIGTRSLSLHFTGCSEDAINSLKEPCYLSS
Keiboba_Pias-1  MRYMNKLRKLKIWCTSSAGSTDWTDLREAIQQFILDEKEANIGTRSLSLHFTGCSEDAINSLKEPCYLSS
```

```
                     770       780       790       800       810       820       830
IRIS_313-10314  LKLHGNFPQLPQFVTSLRGLKELCLSSTKFTTGLLEALSNLSYLQYLKLVADELEKFIIKVQGFPRLLRL
IRIS_313-11786  LKLHGNFPQLPQFVTSLRGLKELCLSSTKFTTGLLEALSNLSYLQYLKLVADELEKFIIKVQGFPRLLRL
IRIS_313-10059  LKLHGNFPQLPQFVTSLRGLKELCLSSTKFTTGLLEALSNLSYLQYLKLVADELEKFIIKVQGFPRLLRL
Sasanishiki     LKLHGNFPQLPQFVTSLRGLKELCLSSTKFTTGLLEALSNLSYLQYLKLVADELEKFIIKVQGFPRLLRL
IRIS_313-8568   LKLHGNFPQLPQFVTSLRGLKELCLSSTKFTTGLLEALSNLSYLQYLKLVADELEKFIIKVQGFPRLLRL
IRIS_313-11480  LKLHGNFPQLPQFVTSLRGLKELCLSSTKFTTGLLEALSNLSYLQYLKLVADELEKFIIKVQGFPRLLRL
IR64            LKLHGNFPQLPQFVTSLRGLKELCLSSTKFTTGLLEALSNLSYLQYLKLVADELEKFIIKVQGFPRLLRL
IRIS_313-10985  LKLHGNFPQLPQFVTSLRGLKELCLSSTKFTTGLLEALSNLSYLQYLKLVADELEKFIIKVQGFPRLLRL
IRIS_313-12048  LKLHGNFPQLPQFVTSLRGLKELCLSSTKFTTGLLEALSNLSYLQYLKLVADELEKFIIKVQGFPRLLRL
CO39            LKLHGNFPQLPQFVTSLRGLKELCLSSTKFTTGL.EALSNLSYLQYLKLVADELEKFIIKVQGFPRLLRL
IRIS_313-12190  LKLHGNFPQLPQFVTSLRGLKELCLSSTKFTTGLLEALSNLSYLQYLKLVADELEKFIIKVQGFPRLLRL
IRIS_313-11360  LKLHGNFPQLPQFVTSLRGLKELCLSSTKFTTGLLEALSNLSYLQYLKLVADELEKFIIKVQGFPRLLRL
IRIS_313-8813   LKLHGNFPQLPQFVTSLRGLKELCLSSTKFTTGLLEALSNLSYLQYLKLVADELEKFIIKVQGFPRLLRL
IRIS_313-7914   LKLHGNFPQLPQFVTSLRGLKELCLSSTKFTTGLLEALSNLSYLQYLKLVADELEKFIIKVQGFPRLLRL
IRIS_313-10738  LKLHGNFPQLPQFVTSLRGLKELCLSSTKFTTGLLEALSNLSYLQYLKLVADELEKFIIKVQGFPRLLCL
AZUCENA         LKLHGNFPQLPQFVTSLRGLKELCLSSTKFTTGLLEALSNLSYLQYLKLVADELEKFIIKVQGFPRLLCL
IRIS_313-10841  LKLHGNFPQLPQFVTSLRGLKELCLSSTKFTTGLLEALSNLSYLQYLKLVADELEKFIIKVQGFPRLLCL
IRIS_313-10879  LKLHGNFPQLPQFVTSLRGLKELCLSSTKFTTGLLEALSNLSYLQYLKLVADELEKFIIKVQGFPRLLRL
IRIS_313-8554   LKLHGNFPQLPQFVTSLRGLKELCLSSTKFTTGLLEALSNLSYLQYLKLVADELEKFIIKVQGFPRLLRL
IRIS_313-12029  LKLHGNFPQLPQFVTSLRGLKELCLSSTKFTTGLLEALSNLSYLQYLKLVADELEKFIIKVQGFPRLLRL
IRIS_313-11708  LKLHGNFPQLPQFVTSLRGLKELCLSSTKFTTGLLEALSNLSYLQYLKLVADELEKFIIKVQGFPRLLRL
Keiboba_Pias-1  LKLHGNFPQLPQFVTSLRGLKELCLSSTKFTTGLLEALSNLSYLQYLKLVADELEKFIIKVQGFPRLLRL
```

```
                  840       850       860       870       880       890       900
IRIS_313-10314  CIVLQYPTFPVIEEGALPFLVTLQLLCKDLHGLSDIQIECFKHLQEVTLHSGVTPATRQEWVKAAKEHPN
IRIS_313-11786  CIVLQYPTFPVIEEGALPFLVTLQLLCKDLHGLSDIQIECFKHLQEVTLHSGVTPATRQEWVKAAKEHPN
IRIS_313-10059  CIVLQYPTFPVIEEGALPFLVTLQLLCKDLHGLSDIQIECFKHLQEVTLHSGVTPATRQEWVKAAKEHPN
Sasanishiki     CIVLQYPTFPVIEEGALPFLVTLQLLCKDLHGLSDIQIECFKHLQEVTLHSGVTPATRQEWVKAAKEHPN
IRIS_313-8568   CIVLQYPTFPVIEEGALPFLVTLQLLCKDLHGLSDIQIECFKHLQEVTLHSGVTPATRQEWVKAAKEHPN
IRIS_313-11480  CIVLQYPTFPVIEEGALPFLVTLQLLCKDLHGLSDIQIECFKHLQEVTLHSGVTPATRQEWVKAAKEHPN
IR64            CIVLQYPTFPVIEEGALPFLVTLQLLCKDLHGLSDIQIECFKHLQEVTLHSGVTPATRQEWVKAAKEHPN
IRIS_313-10985  CIVLQYPTFPVIEEGALPFLVTLQLLCKDLHGLSDIQIECFKHLQEVTLHSGVTPATRQEWVKAAKEHPN
IRIS_313-12048  CIVLQYPTFPVIEEGALPFLVTLQLLCKDLHGLSDIQIECFKHLQEVTLHSGVTPATRQEWVKAAKEHPN
CO39            CIVLQYPTFPVIEEGALPFLVTLQLLCKDLHGLSDIQIECFKHLQEVTLHSGVTPATRQEWVKAAKEHPN
IRIS_313-12190  CIVLQCPTFPVIEEGALPFLVTLQLLCKDLHGLSDIQIECFKHLQEVTLHSGVTPATRQEWVKAAKEHLN
IRIS_313-11360  CIVLQCPTFPVIEEGALPFLVTLQLLCKDLHGLSDIQIECFKHLQEVTLHSGVTPATRQEWVKAAKEHLN
IRIS_313-8813   CIVLQCPTFPVIEEGALPFLVTLQLLCKDLHGLSDIQIECFKHLQEVTLHSGVTPATRQEWVKAAKEHPN
IRIS_313-7914   CIVLQCPTFPVIEEGALPFLVTLQLLCKDLHGLSDIKIECFKHLQEVTLHSGVTPATRQEWVKAAKEHPN
IRIS_313-10738  CIVLQCPTFPVIEEGALPFLVTLQLLCKDLHGLSDIKIECFKHLQEVTLHSGVTPATRQEWVKAAKEHPN
AZUCENA         CIVLQCPTFPVIEEGALPFLVTLQLLCKDLHGLSDIKIECFKHLQEVTLHSGVTPATRQEWVKAAKEHPN
IRIS_313-10841  CIVLQCPTFPVIEEGALPFLVTLQLLCKDLHGLSDIKIECFKHLQEVTLHSGVTPATRQEWVKAAKEHPN
IRIS_313-10879  CIVLQCPTFPVIEEGALPFLVTLQLLCKDLHGLSDIQIECFKHLQEVTLHSGVTPATRQEWVKAAKEHPN
IRIS_313-8554   CIVLQCPTFPVIEEGALPFLVTLQLLCKDLHGLSDIQIECFKHLQEVTLHSGVTPATRQEWVKAAKEHPN
IRIS_313-12029  CIVLQCPTFPVIEEGALPFLVTLQLLCKDLHGLSDIQIECFKHLQEVTLHSGVTPATRQEWVKAAKEHPN
IRIS_313-11708  CIVLQCPTFPVIEEGALPFLVTLQLLCKDLHGLSDIQIECFKHLQEVTLHSGVTPATRQEWVKAAKEHPN
Keiboba_Pias-1  CIVLQCPTFPVIEEGALPFLVTLQLLCKDLHGLSDIQIECFKHLQEVTLHSGVTPATRQEWVKAAKEHPN

                  910       920       930       940       950       960       970
IRIS_313-10314  RPKVLLLKSVDTAESEHTDVDSVMEAVKSETTEYSIAPEGPEQV...NNKMQLDHGLESSSVLNKQNNFA
IRIS_313-11786  RPKVLLLKSVDTAESEHTDVDSVMEAVKSETTEYSIAPEGPEQV...NNKMQLDHGLESSSVLNKQNNFA
IRIS_313-10059  RPKVLLLKSVDTAESEHTDVDSVMEAVKSETTEYSIAPEGPEQV...NNKMQLDHGLESSSVLNKQNNFA
Sasanishiki     RPKVLLLKSVDTAESEHTDVDSVMEAVKSETTEYSIAPEGPEQV...NNKMQLDHGLESSSVLNKQNNFA
IRIS_313-8568   RPKVLLLKSVDTAESEHTDVDSVMEAVKSETTEYSIAPEGPEQV...NNKMQLDHGLESSSVLNKQNNFA
IRIS_313-11480  RPKVLLLKSVDTAESEHTDVDSVMEAVKSETTEYSIAPEGPEQV...NNKMQLDHGLESSSVLNKQNNFA
IR64            RPKVLLLKSVDTAESEHTDVDSVMEAVKSETTEYSIAPEGPEQV...NNKMQLDHGLESSSVLNKQNNFA
IRIS_313-10985  RPKVLLLKSVDTAESEHTDVDSVMEAVKSETTEYSIAPEGPEQV...NNKMQLDHGLESSSVLNKQNNFA
IRIS_313-12048  RPKVLLLKSVDTAESEHTDVDSVMEAVKSETTEYSIAPEGPEQV...NNKMQLDHGLESSSVLNKQNNFA
CO39            RPKVLLLKSVDTAESEHTDVDSVMEAVKSETTEYSIAPEGPEQV...NNKMQLDHGLESSSVLNKQNNFA
IRIS_313-12190  RPKVLLLKSVDTAESEHTDVDSVMEAVKSETTEYSIAPEGPEQVIDMNNKMQLDHRLESSSVLNKQNNFA
IRIS_313-11360  RPKVLLLKSVDTAESEHTDVDSVMEAVKSETTEYSIAPEGPEQVIDMNNKMQLDHGLESSSVLNKQNNFA
IRIS_313-8813   RPKVLLLKSVDTAESEHTDVDSVMEAVKSETTEYSIAPEGPEQVIDMNNKMQLDHGLESSSVLNKQNNFA
IRIS_313-7914   RPKVLLLKSVDTAESEHTDVDSVMEAVKSETTEYSIAPEGPEQVIDMNNKMQLDHGLESSSVLNKQNNFA
IRIS_313-10738  RPKVLLLKSVDTAESEHTDVDSVMEAVKSETTEYSIAPEEPEQVIDMNNKMQLDHGLESSSVLNKQNNFA
AZUCENA         RPKVLLLKSVDTAESEHTDVDSVMEAVKSETTEYSIAPEEPEQVIDMNNKMQLDHGLESSSVLNKQNNFA
IRIS_313-10841  RPKVLLLKSVDTAESEHTDVDSVMEAVKSETTEYSIAPEEPEQVIDMNNKMQLDHGLESSSVLNKQNNFA
IRIS_313-10879  RPKVLLLKSVDTAESEHTDVDSVMEAVKSETTEYSIAPEGPEQVIDMNNKMQLDHGLESSSVLNKQNNFA
IRIS_313-8554   RPKVLLLKSVDTAESEHTDVDSVMEAVKSETTEYSIAPEGPEQVIDMNNKMQLDHGLESSSVLNKQNNFA
IRIS_313-12029  RPKVLLLKSVDTAESEHTDVDSVMEAVKSETTEYSIAPEGPEQVIDMNNKMQLDHGLESSSVLNKQNNFA
IRIS_313-11708  RPKVLLLKSVDTAESEHTDVDSVMEAVKSETTEYSIAPEGPEQVIDMNNKMQLDHGLESSSVLNKQNNFA
Keiboba_Pias-1  RPKVLLLKSVDTAESEHTDVDSVMEAVKSETTEYYIAPEGPEQVIDMNNKMQLDHGLESSSVLNKQNNFA

                    980       990
IRIS_313-10314  DQSSSKDQLHYSFNNMGLSDVSCCE............
IRIS_313-11786  DQSSSKDQLHYSFNNMGLSDVSCCE............
IRIS_313-10059  DQSSSKDQLHYSFNNMGLSDVSCCE............
Sasanishiki     DQSSSKDQLHYSFNNMGLSDVSCCE............
IRIS_313-8568   DQSSSKDQLHYSFNNMGLSDVSCCE............
IRIS_313-11480  DQSSSKDQLHYSFNNMGLSDVSCCE............
IR64            DQSSSKDQLHYSFNNMGLSDVSCCE............
IRIS_313-10985  DQSSSKDQLHYSFNNMGLSDVSCCE............
IRIS_313-12048  DQSSSKDQLHYSFNNMGLSDVSCCE............
CO39            DQSSSKDQLHYSFNNMGLSDVSCCE............
IRIS_313-12190  DQSSSKDQLHYSFNNMGLSDVSPAVS...........
IRIS_313-11360  DQSSSKDQLHYSFNNMGLSDVSPAVS...........
IRIS_313-8813   DQSSSKDQLHYSFNNMGLSDVSPAVS...........
IRIS_313-7914   DQSSSKDQLHYSFNNMGLSDVSPAVS...........
IRIS_313-10738  DQSSSKDQLHYSFNNMGLSDVSPAVS...........
AZUCENA         DQSSSKDQLHYSFNNMGLSDVSPAVS...........
IRIS_313-10841  DQSSSKDQLHYSFNNMGLSDVSPAVS...........
IRIS_313-10879  DQSSSKDQLHYSFNNMGLSDVSPAVS...........
IRIS_313-8554   DQSSSKDQLHYSFNNMGLSDVSPAVS...........
IRIS_313-12029  DQSSSKDQLHYSFNNMGLSDVSPAVS...........
IRIS_313-11708  DQSSSKDQLHYSFNNMGLSDVSPAVS...........
Keiboba_Pias-1  DQSSSKDQLHYSFNNMGLSDVSPAVSELPNGMVPSCT
```

Supplementary Figure 7. RGA4 amino acid alignment. RGA4 amino acid alignment from the 10 accessions sequenced in this article, plus CO39, Sasanishiki, Azucena, Keiboba, and 12 other diverse rice genomes. The location of the MHD motif variant (TYG or MYG) required for RGA4 auto-activity upon release from RGA5 is labeled at alignment position 500.

```
                1        10        20        30        40        50        60        70        80
Sasanishiki     MDAPASFSLGAMGPLLRKLDSLLVAPEIRLPKPLKEGIELLKEDLEEIGVSLVEHSVVDSPTHKARFWMDEVRDLSYHIE
IRIS_313-10059  MDAPASFSLGAMGPLLRKLDSLLVAPEIRLPKPLKEGIELLKEDLEEIGVSLVEHSVVDSPTHKARFWMDEVRDLSYHIE
Aichi           MDAPASFSLGAMGPLLRKLDSLLVAPEIRLPKPLKEGIELLKEDLEEIGVSLVEHSVVDSPTHKARFWMDEVRDLSYHIE
IRIS_313-10985  MDAPASFSLGAMGPLLRKLDSLLVAPEIRLPKPLKEGIELLKEDLEEIGVSLVEHSVVDSPTHKARFWMDEVRDLSYHIE
IRIS_313-11786  MDAPASFSLGAMGPLLRKLDSLLVAPEIRLPKPLKEGIELLKEDLEEIGVSLVEHSVVDSPTHKARFWMDEVRDLSYHIE
IRIS_313-10314  MDAPASFSLGAMGPLLRKLDSLLVAPEIRLPKPLKEGIELLKEDLEEIGVSLVEHSVVDSPTHKARFWMDEVRDLSYHIE
IR64            MDAPASFSLGAMGPLLRKLDSLLVAPEIRLPKPLKEGIELLKEDLEEIGVSLVEHSVVDSPTHKARFWMDEVRDLSYHIE
IRIS_313-11480  MDAPASFSLGAMGPLLRKLDSLLVAPEIRLPKPLKEGIELLKEDLEEIGVSLVEHSVVDSPTHKARFWMDEVRDLSYHIE
93-11           MDAPASFSLGAMGPLLRKLDSLLVAPEIRLPKPLKEGIELLKEDLEEIGVSLVEHSVVDSPTHKARFWMDEVRDLSYHIE
IRIS_313-12048  MDAPASFSLGAMGPLLRKLDSLLVAPEIRLPKPLKEGIELLKEDLEEIGVSLVEHSVVDSPTHKARFWMDEVRDLSYHIE
CO39            MDAPASFSLGAMGPLLRKLDSLLVAPEIRLPKPLKEGIELLKEDLEEIGVSLVEHSVVDSPTHKARFWMDEVRDLSYHIE
IRIS_313-11360  MDAPASFSLGAMGPLLRKLDSLLVAPEIRLPEPLKDGIELLKEDVEEISAALLEQSMVDSPSHRARYWMDEVRDLSYHIE
IRIS_313-12190  MDAPASFSLGAMGPLLRKLDSLLVAPEIRLPEPLKDGIELLKEDVEEISAALLEQSMVDSPSHRARYWMDEVRDLSYHIE
IRIS_313-8813   MDAPASFSLGAMGPLLRKLDSLLVAPEIRLPEPLKDGIELLKEDVEEISAALLEQSMVDSPSHRARYWMDEVRDLSYHIE

                         90       100       110       120       130       140       150       160
Sasanishiki     DCIDTMFSMRSGGDDGKPRSERRHKVGRAKIDGFSKKPKPCTRMARIAELRALVREASERLERYQLGDVCGSSSPVVFTA
IRIS_313-10059  DCIDTMFSMRSGGDDGKPRSERRHKVGRAKIDGFSKKPKPCTRMARIAELRALVREASERLERYQLGDVCGSSSPVVFTA
Aichi           DCIDTMFSMRSGGDDGKPRSERRHKVGRAKIDGFSKKPKPCTRMARIAELRALVREASERLERYQLGDVCGSSSPVVFTA
IRIS_313-10985  DCIDTMFSMRSGGDDGKPRSERRHKVGRAKIDGFSKKPKPCTRMARIAELRALVREASERLERYQLGDVCGSSSPVVFTA
IRIS_313-11786  DCIDTMFSMRSGGDDGKPRSERRHKVGRAKIDGFSKKPKPCTRMARIAELRALVREASERLERYQLGDVCGSSSPVVFTA
IRIS_313-10314  DCIDTMFSMRSGGDDGKPRSERRHKVGRAKIDGFSKKPKPCTRMARIAELRALVREASERLERYQLGDVCGSSSPVVFTA
IR64            DCIDTMFSMRSGGDDGKPRSERRHKVGRAKIDGFSKKPKPCTRMARIAELRALVREASERLERYQLGDVCGSSSPVVFTA
IRIS_313-11480  DCIDTMFSMRSGGDDGKPRSERRHKVGRAKIDGFSKKPKPCTRMARIAELRALVREASERLERYQLGDVCGSSSPVVFTA
93-11           DCIDTMFSMRSGGDDGKPRSERRHKVGRAKIDGFSKKPKPCTRMARIAELRALVREASERLERYQLGDVCGSSSPVVFTA
IRIS_313-12048  DCIDTMFSMRSGGDDGKPRSERRHKVGRAKIDGFSKKPKPCTRMARIAELRALVREASERLERYQLGDVCGSSSPVVFTA
CO39            DCIDTMFSMRSGGDDGKPRSERRHKVGRAKIDGFSKKPKPCTRMARIAELRALVREASERLERYQLGDVCGSSSPVVFTA
IRIS_313-11360  DCIDTMFSVRSVGGDGKPRSVRRHKVGRAKIDGFSKKPKPCTRMARIAELRALVREASERRERYQLGDVCGSSSPVVFTA
IRIS_313-12190  DCIDTMFSVRSVGGDGKPRSVRRHKVGRAKIDGFSKKPKPCTRMARIAELRALVREASERRERYQLGDVCGSSSPVVFTA
IRIS_313-8813   DCIDTMFSVRSVGGDGKPRSVRRHKVGRAKIDGFSKKPKPCTRMARIAELRALVREASERRERYQLGDVCGSSSPVVFTA

                        170       180       190       200       210       220       230       240
Sasanishiki     DGRARPLHHGVSANLVGVDEFKTKLNRWLSDEEGPHLKVAAIVGPAGIGKTALATELYRDHRWQFECRAFVRASRKPDMQ
IRIS_313-10059  DGRARPLHHGVSANLVGVDEFKTKLNRWLSDEEGPHLKVAAIVGPAGIGKTALATELYRDHRWQFECRAFVRASRKPDMQ
Aichi           DGRARPLHHGVSANLVGVDEFKTKLNRWLSDEEGPHLKVAAIVGPAGIGKTALATELYRDHRWQFECRAFVRASRKPDMQ
IRIS_313-10985  DGRARPLHHGVSANLVGVDEFKTKLNRWLSDEEGPHLKVAAIVGPAGIGKTALATELYRDHRWQFECRAFVRASRKPDMQ
IRIS_313-11786  DGRARPLHHGVSANLVGVDEFKTKLNRWLSDEEGPHLKVAAIVGPAGIGKTALATELYRDHRWQFECRAFVRASRKPDMQ
IRIS_313-10314  DGRARPLHHGVSANLVGVDEFKTKLNRWLSDEEGPHLKVAAIVGPAGIGKTALATELYRDHRWQFECRAFVRASRKPDMQ
IR64            DGRARPLHHGVSANLVGVDEFKTKLNRWLSDEEGPHLKVAAIVGPAGIGKTALATELYRDHRWQFECRAFVRASRKPDMQ
IRIS_313-11480  DGRARPLHHGVSANLVGVDEFKTKLNRWLSDEEGPHLKVAAIVGPAGIGKTALATELYRDHRWQFECRAFVRASRKPDMQ
93-11           DGRARPLHHGVSANLVGVDEFKTKLNRWLSDEEGPHLKVAAIVGPAGIGKTALATELYRDHRWQFECRAFVRASRKPDMQ
IRIS_313-12048  DGRARPLHHGVSANLVGVDEFKTKLNRWLSDEEGPHLKVAAIVGPAGIGKTALATELYRDHRWQFECRAFVRASRKPDMQ
CO39            DGRARPLHHGVSANLVGVDEFKTKLNRWLSDEEGPHLKVAAIVGPAGIGKTALATELYRDHRWQFECRAFVRASRKPDMQ
IRIS_313-11360  DGRARPLHHGVSANLVGVDEPKTKLNRWLSDEEGPHLKVAAIVGPAGIGKTALATELYRDNRWQFECRAFVRASRKPDMQ
IRIS_313-12190  DGRARPLHHGVSANLVGVDEPKTKLNRWLSDEEGPHLKVAAIVGPAGIGKTALATELYRDRRWQFECRAFVRASRKPDMQ
IRIS_313-8813   DGRARPLHHGVSANLVGVDEPKTKLNRWLSDEEGPHLKVAAIVGPAGIGKTALATELYRDRRWQFECRAFVRASRKPDMQ

                        250       260       270       280       290       300       310       320
Sasanishiki     RLLGGILSQVQRRQRSSDAYADSTVQSLIDNLREHLQDRRYLIIIDGLWETAVWNIANSAFPDVNSFSRILITADIEQVA
IRIS_313-10059  RLLGGILSQVQRRQRSSDAYADSTVQSLIDNLREHLQDRRYLIIIDGLWETAVWNIANSAFPDVNSFSRILITADIEQVA
Aichi           RLLGGILSQVQRRQRSSDAYADSTVQSLIDNLREHLQDRRYLIIIDGLWETAVWNIANSAFPDVNSFSRILITADIEQVA
IRIS_313-10985  RLLGGILSQVQRRQRSSDAYADSTVQSLIDNLREHLQDRRYLIIIDGLWETAVWNIANSAFPDVNSFSRILITADIEQVA
IRIS_313-11786  RLLGGILSQVQRRQRSSDAYADSTVQSLIDNLREHLQDRRYLIIIDGLWETAVWNIANSAFPDVNSFSRILITADIEQVA
IRIS_313-10314  RLLGGILSQVQRRQRSSDAYADSTVQSLIDNLREHLQDRRYLIIIDGLWETAVWNIANSAFPDVNSFSRILITADIEQVA
IR64            RLLGGILSQVQRRQRSSDAYADSTVQSLIDNLREHLQDRRYLIIIDGLWETAVWNIANSAFPDVNSFSRILITADIEQVA
IRIS_313-11480  RLLGGILSQVQRRQRSSDAYADSTVQSLIDNLREHLQDRRYLIIIDGLWETAVWNIANSAFPDVNSFSRILITADIEQVA
93-11           RLLGGILSQVQRRQRSSDAYADSTVQSLIDNLREHLQDRRYLIIIDGLWETAVWNIANSAFPDVNSFSRILITADIEQVA
IRIS_313-12048  RLLGGILSQVQRRQRSSDAYADSTVQSLIDNLREHLQDRRYLIIIDGLWETAVWNIANSAFPDVNSFSRILITADIEQVA
CO39            RLLGGILSQVQRRQRSSDAYADSTVQSLIDNLREHLQDRRYLIIIDGLWETAVWNIANSAFPDVNSFSRILITADIEQVA
IRIS_313-11360  RLLGGILSQVQRRQRSSDAYADSTVQSLIDNLREHLQDRRYLIIIDGLWETAVWNIANSAFPDVNSFSRILITADIEQVA
IRIS_313-12190  RLLGGILSQVQRRQRSSDAYADSTVQSLIDNLREHLQDRRYLIIIDGLWETAVWNIANSAFPDVNSFSRILITADIEQVA
IRIS_313-8813   RLLGGILSQVQRRQRSSDAYADSTVQSLIDNLREHLQDRRYLIIIDGLWETAVWNIANSAFPDVNSFSRILITADIEQVA

                        330       340       350       360       370       380       390       400
Sasanishiki     LECCGYKYDYIMRMEPLGSLDSKKVFFNKVFGSEDQCPPELKEVSNTILEKCGGLPLAIISIAGLLGSQPENPVLWDYVT
IRIS_313-10059  LECCGYKYDYIMRMEPLGSLDSKKVFFNKVFGSEDQCPPELKEVSNTILEKCGGLPLAIISIAGLLGSQPENPVLWDYVT
Aichi           LECCGYKYDYIMRMEPLGSLDSKKVFFNKVFGSEDQCPPELKEVSNTILEKCGGLPLAIISIAGLLGSQPENPVLWDYVT
IRIS_313-10985  LECCGYKYDYIMRMEPLGSLDSKKVFFNKVFGSEDQCPPELKEVSNTILEKCGGLPLAIISIAGLLGSQPENPVLWDYVT
IRIS_313-11786  LECCGYKYDYIMRMEPLGSLDSKKVFFNKVFGSEDQCPPELKEVSNTILEKCGGLPLAIISIAGLLGSQPENPVLWDYVT
IRIS_313-10314  LECCGYKYDYIMRMEPLGSLDSKKVFFNKVFGSEDQCPPELKEVSNTILEKCGGLPLAIISIAGLLGSQPENPVLWDYVT
IR64            LECCGYKYDYIMRMEPLGSLDSKKVFFNKVFGSEDQCPPELKEVSNTILEKCGGLPLAIISIAGLLGSQPENPVLWDYVT
IRIS_313-11480  LECCGYKYDYIMRMEPLGSLDSKKVFFNKVFGSEDQCPPELKEVSNTILEKCGGLPLAIISIAGLLGSQPENPVLWDYVT
93-11           LECCGYKYDYIMRMEPLGSLDSKKVFFNKVFGSEDQCPPELKEVSNTILEKCGGLPLAIISIAGLLGSQPENPVLWDYVT
IRIS_313-12048  LECCGYKYDYIMRMEPLGSLDSKKVFFNKVFGSEDQCPPELKEVLNTILEKCGGLPLAIISIAGLLGSQPENPVLWDYVT
CO39            LECCGYKYDYIMRMEPLGSLDSKKVFFNKVFGSEDQCPPELKEVLNTILEKCGGLPLAIISIAGLLGSQPENPVLWDYVT
IRIS_313-11360  LECCGYKYDYIMRMEPLGSLDSKKVFFNKVFGSEDQCPPELKEVSNTILEKCGGLPLAIVSIAGLLGSQPENPVLWDYVT
IRIS_313-12190  LECCGYKYDYIMRMEPLGSLDSKKVFFNKVFGSEDQCPPELKEVSNTILEKCGGLPLAIVSIAGLLGSQPKNPVLWDYVT
IRIS_313-8813   LECCGYKYDYIMRMEPLGSLDSKKVFFNKVFGSEDQCPPELKEVSNTILEKCGGLPLAIVSIAGLLGSQPENPVLWDYVT
```

```
                        410       420       430       440       450       460       470       480
                          .         .         .         .         .         .         .         .
Sasanishiki      KYLCSSLGTNPTLKDVVKETLNLSYNSLPHPFKTCLLYLGMYPDGHIMLKADLMKQWSAEGFVSANEAKDTEEIVDKYFD
IRIS_313-10059   KYLCSSLGTNPTLKDVVKETLNLSYNSLPHPFKTCLLYLGMYPDGHIMLKADLMKQWSAEGFVSANEAKDTEEIVDKYFD
Aichi Asahi      KYLCSSLGTNPTLKDVVKETLNLSYNSLPHPFKTCLLYLGMYPDGHIMLKADLMKQWSAEGFVSANEAKDTEEIVDKYFD
IRIS_313-10985   KYLCSSLGTNPTLKDVVKETLNLSYNSLPHPFKTCLLYLGMYPDGHIMLKADLMKQWSAEGFVSANEAKDTEEIVDKYFD
IRIS_313-11786   KYLCSSLGTNPTLKDMVKETLNLSYNSLPHPFKTCLLYLGMYPDGHIMLKADLMKQWSAEGFVSANEAKDTEEIVDKYFD
IRIS_313-10314   KYLCSSLGTNPTLKDMVKETLNLSYNSLPHPFKTCLLYLGMYPDGHIMLKADLMKQWSAEGFVSANEAKDTEEIVDKYFD
IR64             KYLCSSLGTNPTLKDMVKETLNLSYNSLPHPFKTCLLYLGMYPDGHIMLKADLMKQWSAEGFVSANEAKDTEEIVDKYFD
IRIS_313-11480   KYLCSSLGTNPTLKDMVKETLNLSYNSLPHPFKTCLLYLGMYPDGHIMLKADLMKQWSAEGFVSANEAKDTEEIVDKYFD
93-11            KYLCSSLGTNPTLKDMVKETLNLSYNSLPHPFKTCLLYLGMYPDGHIMLKADLMKQWSAEGFVSANEAKDTEEIVDKYFD
IRIS_313-12048   KYLCSSLGTNPTLKDVVKETLNLSYNSLPHPFKTCLLYLGMYPDGHIMLKADLMKQWSAEGFVSANEAKDTEEIVDKYFD
CO39             KYLCSSLGTNPTLKDVVKETLNLSYNSLPHPFKTCLLYLGMYPDGHIMLKADLMKQWSAEGFVSANEAKDTEEIVDKYFD
IRIS_313-11360   KYLCSSLGTNPTLKDVVNETLNLSYNSLPHPFKTCLLYLGMYPDGHIMLKADLMKQWSAEGFVSANEAKDTEEIVDKYFD
IRIS_313-12190   KFLCSSLGTNPTLKDVVKETLNLSYNSLPHPFKTCLLYLGMYPDGHIMLKADLMKQWSAEGFISANEAKDTEEIVDKYFD
IRIS_313-8813    KFLCSSLGTNPTLKDVVKETLNLSYNSLPHPFKTCLLYLGMYPDGHIMLKADLMKQWSAEGFVSANEAKDTEEIVDKYFD
```

```
                        490       500       510       520       530       540       550       560
                          .         .         .         .         .         .         .         .
Sasanishiki      ELVNRGILEPVEINKNGKVLSCTLHHAVHDLVMPKFNDDKFTMSVDYSQTITGPSTMVRRLSLHFSSTRYATKPAGIILS
IRIS_313-10059   ELVNRGILEPVEINKNGKVLSCTLHHAVHDLVMPKFNDDKFTMSVDYSQTITGPSTMVRRLSLHFSSTRYATKPAGIILS
Aichi Asahi      ELVNRGILEPVEINKNGKVLSCTLHHAVHDLVMPKFNDDKFTMSVDYSQTITGPSTMVRRLSLHFSSTRYATKPAGIILS
IRIS_313-10985   ELVNRGILEPVEINKNGKVLSCTLHHAVHDLVMPKFNDDKFTMSVDYSQTITGPSTMVRRLSLHFSSTRYATKPAGIILS
IRIS_313-11786   ELVNRGILEPVEINKNGKVLSCTLHHAVHDLVMPKFNDDKFTMSVDYSQTITGPSTMVRRLSLHFSSTRYATKPAGIILS
IRIS_313-10314   ELVNRGILEPVEINKNGKVLSCTLHHAVHDLVMPKFNDDKFTMSVDYSQTITGPSTMVRRLSLHFSSTRYATKPAGIILS
IR64             ELVNRGILEPVEINKNGKVLSCTLHHAVHDLVMPKFNDDKFTMSVDYSQTITGPSTMVRRLSLHFSSTRYATKPAGIILS
IRIS_313-11480   ELVNRGILEPVEINKNGKVLSCTLHHAVHDLVMPKFNDDKFTMSVDYSQTITGPSTMVRRLSLHFSSTRYATKPAGIILS
93-11            ELVNRGILEPVEINKNGKVLSCTLHHAVHDLVMPKFNDDKFTMSVDYSQTITGPSTMVRRLSLHFSSTRYATKPAGIILS
IRIS_313-12048   ELVNRGILEPVEINKNGKVLSCTLHHAVHDLVMPKFNDDKFTMSVDYSQTITGPSTMVRRLSLHFSSTRYATKPAGIILS
CO39             ELVNRGILEPVEINKNGKVLSCTLHHAVHDLVMPKFNDDKFTMSVDYSQTITGPSTMVRRLSLHFSSTRYATKPAGIILS
IRIS_313-11360   ELVNRGILEPVEINKNGKVLSCTLHHAVHDLVMPKFNDDKFTMSVDYSQTITGTSTMVRRLSLHFSSTRYATKPAGIILS
IRIS_313-12190   ELVNRGILEPVEINKNGKVLSCTLHHAVHDLVMPKFNDDKFTMSVDYSQTITGPSTMVRRLSLHFSSTRYATKPAGIILS
IRIS_313-8813    ELVNRGILEPVEINKNGKVLSCTLHHAVHDLVMPKFNDDKFTMSVDYSQTITGPSTMVRRLSLHFSSTRYATKPAGIILS
```

```
                        570       580       590       600       610       620       630       640
                          .         .         .         .         .         .         .         .
Sasanishiki      RVRSLAFFGLLNCMPCIGEFKLLRVLILEFWGSHGEQRSLNLIPVCRLFQLRYLKTSGDVVVQLPAQISGLQYLETLEID
IRIS_313-10059   RVRSLAFFGLLNCMPCIGEFKLLRVLILEFWGSHGEQRSLNLIPVCRLFQLRYLKTSGDVVVQLPAQISGLQYLETLEID
Aichi Asahi      RVRSLAFFGLLNCMPCIGEFKLLRVLILEFWGSHGEQRSLNLIPVCRLFQLRYLKTSGDVVVQLPAQISGLQYLETLEID
IRIS_313-10985   RVRSLAFFGLLNCMPCIGEFKLLRVLILEFWGSHGEQRSLNLIPVCRLFQLRYLKTSGDVVVQLPAQISGLQYLETLEID
IRIS_313-11786   RVRSLAFFGLLNCMPCIGEFKLLRVLILEFWGSHGEQRSLNLIPVCRLFQLRYLKTSGDVVVQLPAQISGLQYLETLEID
IRIS_313-10314   RVRSLAFFGLLNCMPCIGEFKLLRVLILEFWGSHGEQRSLNLIPVCRLFQLRYLKTSGDVVVQLPAQISGLQYLETLEID
IR64             RVRSLAFFGLLNCMPCIGEFKLLRVLILEFWGSHGEQRSLNLIPVCRLFQLRYLKTSGDVVVQLPAQISGLQYLETLEID
IRIS_313-11480   RVRSLAFFGLLNCMPCIGEFKLLRVLILEFWGSHGEQRSLNLIPVCRLFQLRYLKTSGDVVVQLPAQISGLQYLETLEID
93-11            RVRSLAFFGLLNCMPCIGEFKLLRVLILEFWGSHGEQRSLNLIPVCRLFQLRYLKTSGDVVVQLPAQISGLQYLETLEID
IRIS_313-12048   RVRSLAFFGLLNCMPCIGEFKLLRVLILEFWGSHGEQRSLNLIPVCRLFQLRYLKTSGDVVVQLPAQISGLQYLETLEID
CO39             RVRSLAFFGLLNCMPCIGEFKLLRVLILEFWGSHGEQRSLNLIPVCRLFQLRYLKTSGDVVVQLPAQISGLQYLETLEID
IRIS_313-11360   RVRSLAFFGLLNCMPCIGEFKLLRVLILEFWGSHGEQRSLNLIPVCRLFQLRYLKTSGDVVVQLPAQISGLQYLETLEID
IRIS_313-12190   RVRSLAFFGLLNCMPCIGEFKLLRVLILEFWGSHGEQRSLNLIPVCILFQLRYLKTSGDVVVQLPAQISGLQYLETLEID
IRIS_313-8813    RVRSLAFFGLLNCMPCIGEFKLLRVLILEFWGSHGEQRSLNLIPVCILFQLRYLKTSGDVVVQLPAQISGLQYLETLEID
```

```
                        650       660       670       680       690       700       710       720
                          .         .         .         .         .         .         .         .
Sasanishiki      ARVSAVPFDLVHLPNLLHLQLQDETKLPDGIGCMRSLRTLQYFDLGNNSVDNLRGLGELTNLQDLHLSYSAPSSNEGLMI
IRIS_313-10059   ARVSAVPFDLVHLPNLLHLQLQDETKLPDGIGCMRSLRTLQYFDLGNNSVDNLRGLGELTNLQDLHLSYSAPSSNEGLMI
Aichi Asahi      ARVSAVPFDLVHLPNLLHLQLQDETKLPDGIGCMRSLRTLQYFDLGNNSVDNLRGLGELTNLQDLHLSYSAPSSNEGLMI
IRIS_313-10985   ARVSAVPFDLVHLPNLLHLQLQDETKLPDGIGCMRSLRTLQYFDLGNNSVDNLRGLGELTNLQDLHLSYSAPSSNEGLMI
IRIS_313-11786   ARVSAVPFDLVHLPNLLHLQLQDETKLPDGIGCMRSLRTLQYFDLGNNSVDNLRGLGELTNLQDLHLSYSAPSSNEGLMI
IRIS_313-10314   ARVSAVPFDLVHLPNLLHLQLQDETKLPDGIGCMRSLRTLQYFDLGNNSVDNLRGLGELTNLQDLHLSYSAPSSNEGLMI
IR64             ARVSAVPFDLVHLPNLLHLQLQDETKLPDGIGCMRSLRTLQYFDLGNNSVDNLRGLGELTNLQDLHLSYSAPSSNEGLMI
IRIS_313-11480   ARVSAVPFDLVHLPNLLHLQLQDETKLPDGIGCMRSLRTLQYFDLGNNSVDNLRGLGELTNLQDLHLSYSAPSSNEGLMI
93-11            ARVSAVPFDLVHLPNLLHLQLQDETKLPDGIGCMRSLRTLQYFDLGNNSVDNLRGLGELTNLQDLHLSYSAPSSNEGLMI
IRIS_313-12048   ARVSAVPFDLVHLPNLLHLQLQDETKLPDGIGCMRSLRTLQYFDLGNNSVDNLRGLGELTNLQDLHLSYSAPSSNEGLMI
CO39             ARVSAVPFDLVHLPNLLHLQLQDETKLPDGIGCMRSLRTLQYFDLGNNSVDNLRGLGELTNLQDLHLSYSAPSSNEGLMI
IRIS_313-11360   ARVSAVPFDLVHLPNLLHLQLQDETKLPDGIGCMRSLRTLQYFDLGNNSVDNLRGLGELTNLQDLHLSYSAPSSNEGLMI
IRIS_313-12190   ARVSAVPFDLVHLPNLLHLQLQDETKLPDGIGCMRSLRTLQYFDLGNNSVDNLRGLGELTNLQDLHLSYSAPSSNEGLMI
IRIS_313-8813    ARVSAVPFDLVHLPNLLHLQLQDETKLPDGIGCMRSLRTLQYFDLGNNSVDNLRGLGELTNLQDLHLSYSAPSSNEGLMI
```

```
                        730       740       750       760       770       780       790       800
                          .         .         .         .         .         .         .         .
Sasanishiki      NLNAITSSLSRLSNLKSLILSPGAISMVIFFDISSIISVVPVFLQRLELLPPICIFCRLPKSIGQLHKLCILKVSVRELL
IRIS_313-10059   NLNAITSSLSRLSNLKSLILSPGAISMVIFFDISSIISVVPVFLQRLELLPPICIFCRLPKSIGQLHKLCILKVSVRELL
Aichi Asahi      NLNAITSSLSRLSNLKSLILSPGAISMVIFFDISSIISVVPVFLQRLELLPPICIFCRLPKSIGQLHKLCILKVSVRELL
IRIS_313-10985   NLNAITSSLSRLSNLKSLILSPGAISMVIFFDISSIISVVPVFLQRLELLPPICIFCRLPKSIGQLHKLCILKVSVRELL
IRIS_313-11786   NLNAITSSLSRLSNLKSLILSPGAISMVIFFDISSIISVVPVFLQRLELLPPICIFCRLPKSIGQLHKLCILKVSVRELL
IRIS_313-10314   NLNAITSSLSRLSNLKSLILSPGAISMVIFFDISSIISVVPVFLQRLELLPPICIFCRLPKSIGQLHKLCILKVSVRELL
IR64             NLNAITSSLSRLSNLKSLILSPGAISMVIFFDISSIISVVPVFLQRLELLPPICIFCRLPKSIGQLHKLCILKVSVRELL
IRIS_313-11480   NLNAITSSLSRLSNLKSLILSPGAISMVIFFDISSIISVVPVFLQRLELLPPICIFCRLPKSIGQLHKLCILKVSVRELL
93-11            NLNAITSSLSRLSNLKSLILSPGAISMVIFFDISSIISVVPVFLQRLELLPPICIFCRLPKSIGQLHKLCILKVSVRELL
IRIS_313-12048   NLNAITSSLSRLSNLKSLILSPGAISMVIFFDISSIISVVPVFLQRLELLPPICIFCRLPKSIGQLHKLCILKVSVRELL
CO39             NLNAITSSLSRLSNLKSLILSPGAISMVIFFDISSIISVVPVFLQRLELLPPICIFCRLPKSIGQLHKLCILKVSVRELL
IRIS_313-11360   NLNAITSSLSRLSNLKSLILSPGAISMVIFFDISSIISVVPVFLQRLELLPPICIFCRLPKSIGQLHKLCILKVSVRELL
IRIS_313-12190   NLNAITSSLSRLSNLKSLILSPGAISMVFFFDISSIISMVPVFLQRLELLPPICIFCRLPKSIGQLHKLCILKVSVRELL
IRIS_313-8813    NLNAITSSLSRLSNLKSLILSPGAISMVFFFDISSIISMVPVFLQRLELLPPICIFCRLPKSIGQLHKLCILKVSVRELL
```

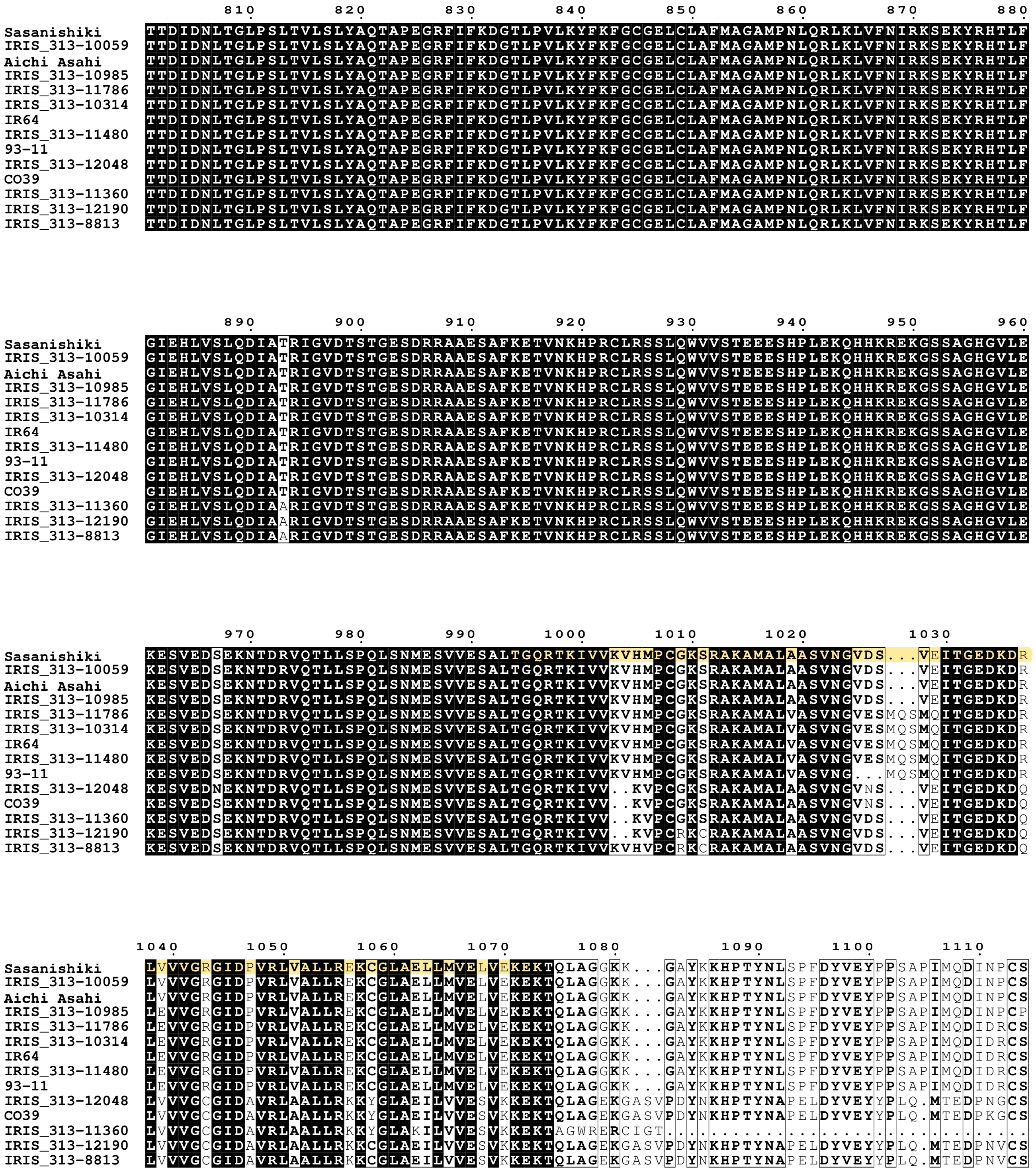

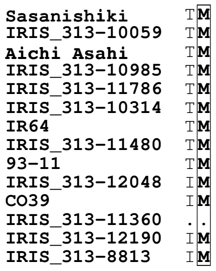

Supplementary Figure 8. RGA5 amino acid alignment. Where homology was detected to *RGA5*, amino acid sequence was extracted from any of the 10 accessions sequenced in this article, plus CO39, Sasanishiki, Aichi Asahi, 93-11 and 12 other diverse rice genomes. Where accessions did not contain *RGA5*, the *Pias-2* gene was observed and the protein sequence encoded by this gene can be found in a separate supplementary figure. The location of the HMA domain is highlighted in the Sasanishiki AA sequence.

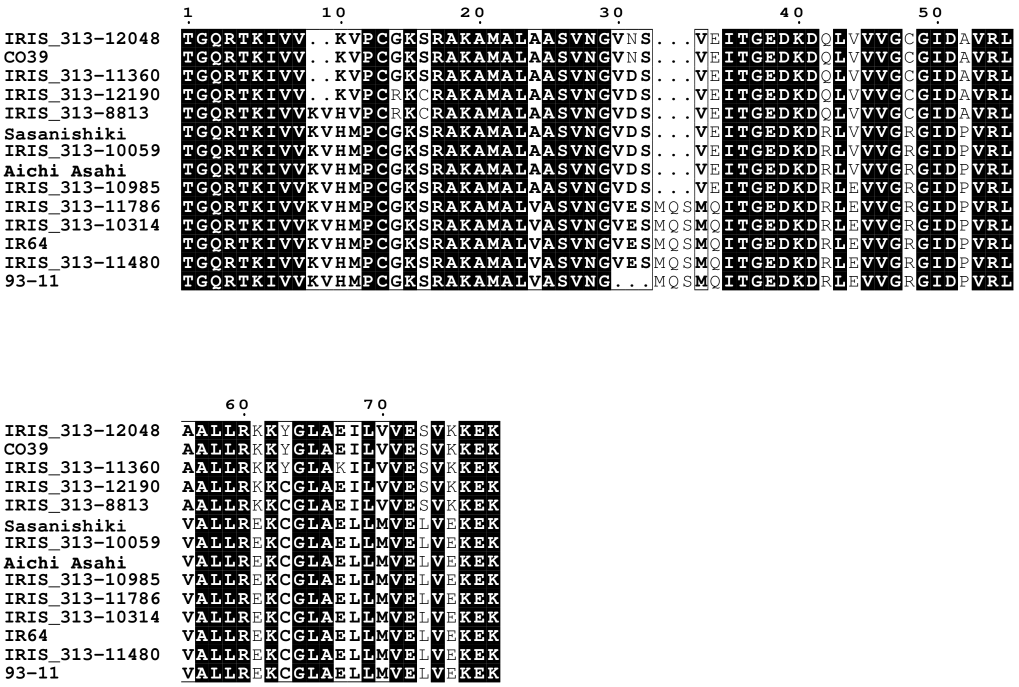

Supplementary Figure 9. RGA5 HMA domain amino acid alignment. Where homology was detected to *RGA5*, amino acid sequence was extracted from any of the 10 accessions sequenced in this article, plus CO39, Sasanishiki, Aichi Asahi, 93-11 and 12 other diverse rice genomes and the HMA domain sequence was extracted.

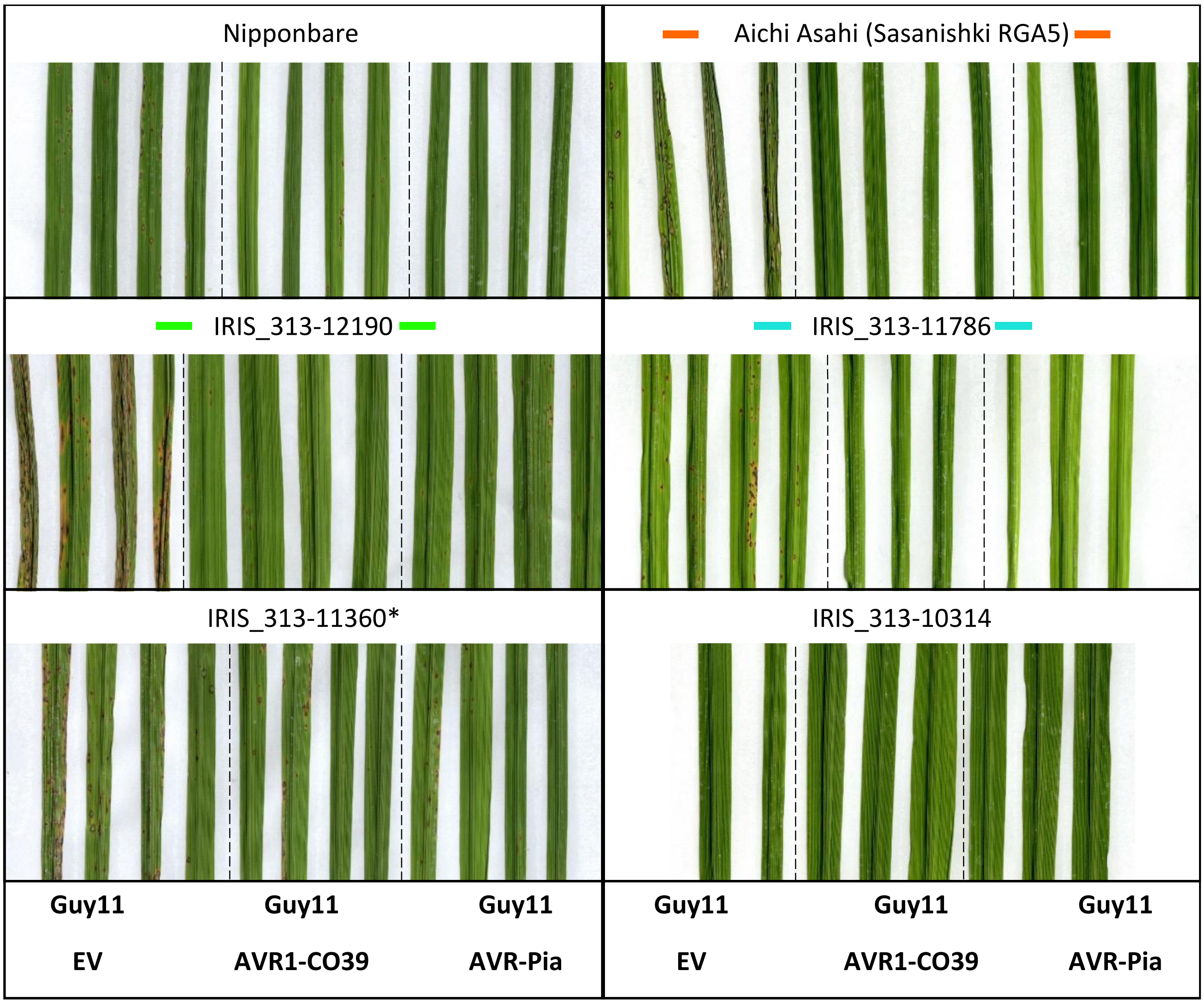

Supplementary Figure 10. C923-49 resistance associated accessions replicate infection test. Infection testing of C923-49 resistance associated accessions with transgenic *M. Oryzae* isolates expressing AVR1-CO39, AVR-Pia or carrying an empty vector (EV). Colored lines either side of accession names match the HMA domain sequence aligned in **Fig. 3 a** and modelled in **Fig. 3 b/ c**. IRIS_313-11360* contains a frameshift mutation after the HMA domain of *Rga5*.

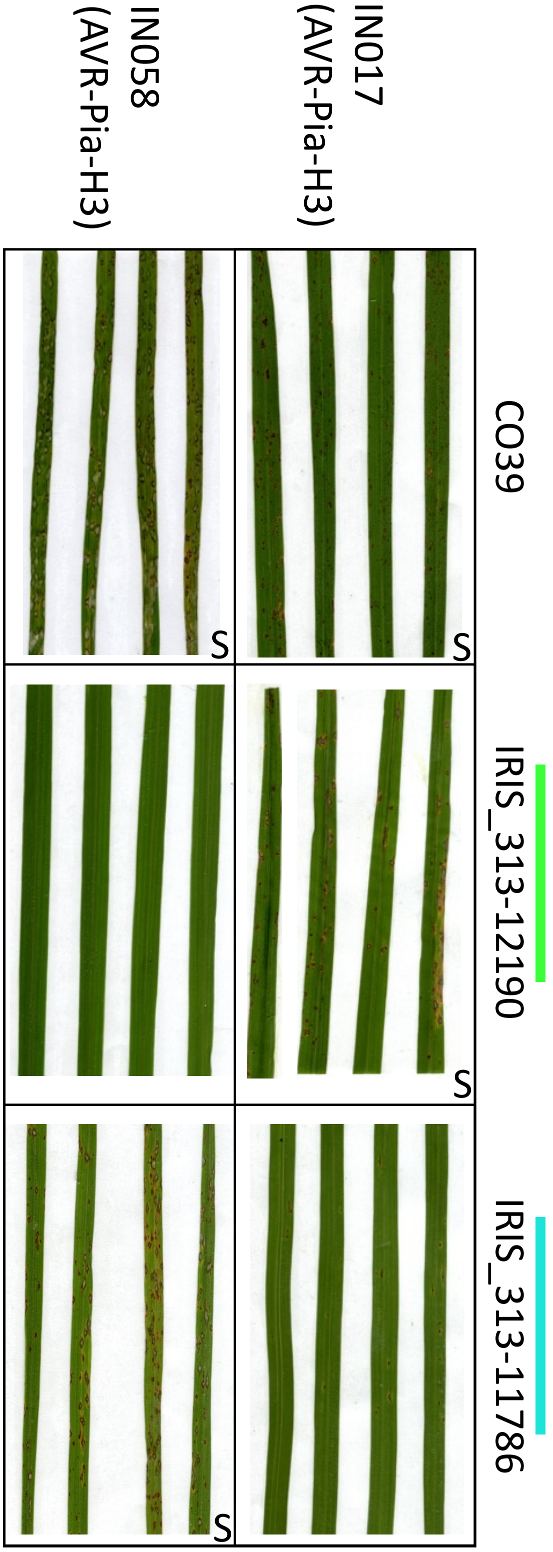

Supplementary Figure 11. The newly identified alleles of *Pia* from IRIS_313-11786 (variant 3) and IRIS_313-12190 (variant 5) do not confer resistance to *AVR-Pia-H3* expressing isolates. *AVR-Pia-H3* expressing isolates IN017 and IN058 were used to infect the novel *Pia* variant containing accessions IRIS_313-12190 and IRIS_313-11786. three-week-old plants were infected by spray inoculation. Images are taken seven days post inoculation. Panels labelled with an S indicate that a susceptibility response was observed between the isolate and genotype shown.

```
                1        10        20        30        40        50        60        70        80
IRIS_313-11163  MDAPVSFSLGAMGPLLRKLDSLPVAPEIRLPEPLKDGIELLKEDLEEIGAAVVEQSTVDSPSHRARYWMDEVRDLSYHIE
IRIS_313-10879  MDAPVSFSLGAMGPLLRKLDSLPVAPEIRLPEPLKDGIELLKEDLEEIGAAVVEQSTVDSPSHRARYWMDEVRDLSYHIE
IRIS_313-8554   MDAPVSFSLGAMGPLLRKLDSLPVAPEIRLPEPLKDGIELLKEDLEEIGAAVVEQSTVDSPSHRARYWMDEVRDLSYHIE
IRIS_313-12029  MDAPVSFSLGAMGPLLRKLDSLPVAPEIRLPEPLKDGIELLKEDLEEIGAAVVEQSTVDSPSHRARYWMDEVRDLSYHIE
Keiboba_Pias-2  MDAPVSFSLGAMGPLLRKLDSLPVAPEIRLPEPLKDGIELLKEDLEEIGAAVVEQSTVDSPSHRARYWMDEVRDLSYHIE
IRIS_313-10738  MDAPVSFSLGAMGPLLRKLDSLPVAPEIRLPEPLKDGIELLKEDLEEIGAALVEQSMVDSPSHRARYWMDEVRDLSYHIE
IRIS_313-11127  MDAPVSFSLGAMGPLLRKLDSLPVAPEIRLPEPLKDGIELLKEDLEEIGAALVEQSMVDSPSHRARYWMDEVRDLSYHIE
IRIS_313-10841  MDAPVSFSLGAMGPLLRKLDSLPVAPEIRLPEPLKDGIELLKEDLEEIGAALVEQSMVDSPSHRARYWMDEVRDLSYHIE
Azucena         MDAPVSFSLGAMGPLLRKLDSLPVAPEIRLPEPLKDGIELLKEDLEEIGAALVEQSMVDSPSHRARYWMDEVRDLSYHIE
Nipponbare      MDAPVSFSLGAMGPLLRKLDSLPVAPEIRLPEPLKDGIELLKEDLEEIGAALVEQSMVDSPSHRARYWMDEVRDLSYHIE
IRIS_313-7914   MDAPVSFSLGAMGPLLRKLDSLPVAPEIRLPEPLKDGIELLKEDLEEIGAALVEQSMVDSPSHRARYWMDEVRDLSYHIE
IRIS_313-11708  MDAPVSFSLGAMGPLLRKLDSLPVAPEIRLPEPLKDGIELLKEDLEEIGAALVEQSMVDSPSHRARYWMDEVRDLSYHIE

                         90       100       110       120       130       140       150       160
IRIS_313-11163  DCIDTMFSMRCGGDDGKPRSVRRHKVGRVKVDGFSKTQKPCTRLARIAELRALVREASERHERYQLGDGRASSSSSSSHR
IRIS_313-10879  DCIDTMFSMRCGGDDGKPRSVRRHKVGRVKVDGFSKTQKPCTRLARIAELRALVREASERHERYQLGDGRASSSSSSSHR
IRIS_313-8554   DCIDTMFSMRCGGDDGKPRSVRRHKVGRVKVDGFSKTQKPCTRLARIAELRALVREASERHERYQLGDGRASSSSSSSHR
IRIS_313-12029  DCIDTMFSMRCGGDDGKPRSVRRHKVGRVKVDGFSKTQKPCTRLARIAELRALVREASERHERYQLGDGRASSSSSSSHR
Keiboba_Pias-2  DCIDTMFSMRCGGDDGKPRSVRRHKVGRVKVDGFSKTQKPCTRLARIAELRALVREASERHERYQLGDGRASSSSSSSHR
IRIS_313-10738  DCIDTMFSMRCGGDDGKPRSVRRHKVGRVKVDGFSKTQKPCTRLARIAELRALVREASERHERYQLGDGRASSSSSSSHR
IRIS_313-11127  DCIDTMFSMRCGGDDGKPRSVRRHKVGRVKVDGFSKTQKPCTRLARIAELRALVREASERHERYQLGDGRASSSSSSSHR
IRIS_313-10841  DCIDTMFSMRCGGDDGKPRSVRRHKVGRVKVDGFSKTQKPCTRLARIAELRALVREASERHERYQLGDGRASSSSSSSHR
Azucena         DCIDTMFSMRCGGDDGKPRSVRRHKVGRVKVDGFSKTQKPCTRLARIAELRALVREASERHERYQLGDGRASSSSSSSHR
Nipponbare      DCIDTMFSMRCGGDDGKPRSVRRHKVGRVKVDGFSKTQKPCTRLARIAELRALVREASERHERYQLGDGRASSSSSSSHR
IRIS_313-7914   DCIDTMFSMRRGGDDGKPRSVRRHKVGRVKVDGFSKTQKPCTRLARIAELRALVREASERHERYQLGDGRASSSSSSSHR
IRIS_313-11708  DCIDTMFSMRCGGDDGKPRSVRRHKVGRVKVDGFSKTQKPCTRLARIAELRALVREASERHERYQLGDGRASSSSSSSHR

                        170       180       190       200       210       220       230       240
IRIS_313-11163  VFTAHGQVPAPCRNLVGMDEPKTKLTNMLTDEAELHMKVVCILGSAGIGKTTLAEQVYRKLRWQFDCHAFVRASRKPDMR
IRIS_313-10879  VFTAHGQVPAPCRNLVGMDEPKTKLTNMLTDEAELHMKVVCILGSAGIGKTTLAEQVYRKLRWQFDCHAFVRASRKPDMR
IRIS_313-8554   VFTAHGQVPAPCRNLVGMDEPKTKLTNMLTDEAELHMKVVCILGSAGIGKTTLAEQVYRKLRWQFDCHAFVRASRKPDMR
IRIS_313-12029  VFTAHGQVPAPCRNLVGMDEPKTKLTNMLTDEAELHMKVVCILGSAGIGKTTLAEQVYRKLRWQFDCHAFVRASRKPDMR
Keiboba_Pias-2  VFTAHGQVPAPCRNLVGMDEPKTKLTNMLTDEAELHMKVVCILGSAGIGKTTLAEQVYRKLRWQFDCHAFVRASRKPDMR
IRIS_313-10738  VFTAHGQVPAPCRNLVGMDEPKTKLTNMLTDEAELHMKVVCILGSAGIGKTTLAEQVYRKLRWQFDCHAFVRASRKPDMR
IRIS_313-11127  VFTAHGQVPAPCRNLVGMDEPKTKLTNMLTDEAELHMKVVCILGSAGIGKTTLAEQVYRKLRWQFDCHAFVRASRKPDMR
IRIS_313-10841  VFTAHGQVPAPCRNLVGMDEPKTKLTNMLTDEAELHMKVVCILGSAGIGKTTLAEQVYRKLRWQFDCHAFVRASRKPDMR
Azucena         VFTAHGQVPAPCRNLVGMDEPKTKLTNMLTDEAELHMKVVCILGSAGIGKTTLAEQVYRKLRWQFDCHAFVRASRKPDMR
Nipponbare      VFTAHGQVPAPCRNLVGMDEPKTKLTNMLTDEAELHMKVVCILGSAGIGKTTLAEQVYRKLRWQFDCHAFVRASRKPDMR
IRIS_313-7914   VFTAHGQVPAPCRNLVGMDEPKTKLTNMLTDEAELHMKVVCILGSAGIGKTTLAEQVYRKLRWQFDCHAFVRASRKPDMR
IRIS_313-11708  VFTAHGQVPAPCRNLVGMDEPKTKLTNMLTDEAELHMKVVCILGSAGIGKTTLAEQVYRKLRWQFDCHAFVRASRKPDMR

                        250       260       270       280       290       300       310       320
IRIS_313-11163  RLLGAILSQVQPRIRISDTSTVQSLIDNLWEYLQKKRYFIVIDELYETATWDIITSAFPEDNNCSRIMTTAGIEGVALEC
IRIS_313-10879  RLLGAILSQVQPRIRISDTSTVQSLIDNLWEYLQKKRYFIVIDELYETATWDIITSAFPEDNNCSRIMTTAGIEGVALEC
IRIS_313-8554   RLLGAILSQVQPRIRISDTSTVQSLIDNLWEYLQKKRYFIVIDELYETATWDIITSAFPEDNNCSRIMTTAGIEGVALEC
IRIS_313-12029  RLLGAILSQVQPRIRISDTSTVQSLIDNLWEYLQKKRYFIVIDELYETATWDIITSAFPEDNNCSRIMTTAGIEGVALEC
Keiboba_Pias-2  RLLGAILSQVQPRIRISDTSTVQSLIDNLWEYLQKKRYFIVIDELYETATWDIITSAFPEDNNCSRIMTTAGIEGVALEC
IRIS_313-10738  RLLGAILSQVQLRIRISDTSTVQSLIDNLWEYLQKKRYFIVIDELYETATWDIITSAFPEDNNCSRIMTTAGIEGVALEC
IRIS_313-11127  RLLGAILSQVQLRIRISDTSTVQSLIDNLWEYLQKKRYFIVIDELYETATWDIITSAFPEDNNCSRIMTTAGIEGVALEC
IRIS_313-10841  RLLGAILSQVQLRIRISDTSTVQSLIDNLWEYLQKKRYFIVIDELYETATWDIITSAFPEDNNCSRIMTTAGIEGVALEC
Azucena         RLLGAILSQVQLRIRISDTSTVQSLIDNLWEYLQKKRYFIVIDELYETATWDIITSAFPEDNNCSRIMTTAGIEGVALEC
Nipponbare      RLLGAILSQVQLRIRISDTSTVQSLIDNLWEYLQKKRYFIVIDELYETATWDIITSAFPEDNNCSRIMTTAGIEGVALEC
IRIS_313-7914   RLLGAILSQVQPRIRISDTSTVQSLIDNLWEYLQKKRYFIVIDELYETATWDIITSAFPEDNNCSRIMTTAGIEGVALEC
IRIS_313-11708  RLLGAILSQVQPRIRISDTSTVQSLIDNLWEYLQKKRYFIVIDELYETATWDIITSAFPEDNNCSRIMTTAGIEGVALEC

                        330       340       350       360       370       380       390       400
IRIS_313-11163  CSYHSVNIFQMKPLGLDDSAKLFFNRVFGSEQQCPYELSEVSYRITAKCGGLPLAVIIIAGLLASLPCKTELWYNIDGCL
IRIS_313-10879  CSYHSVNIFKMKPLGLDDSAKLFFNRVFGSEQQCPYELSEVSYRITAKCGGLPLAVIIIAGLLASLPCKTELWYNIDGCL
IRIS_313-8554   CSYHSVNIFKMKPLGLDDSAKLFFNRVFGSEQQCPYELSEVSYRITAKCGGLPLAVIIIAGLLASLPCKTELWYNIDGCL
IRIS_313-12029  CSYHSVNIFKMKPLGLDDSAKLFFNRVFGSEQQCPYELSEVSYRITAKCGGLPLAVIIIAGLLASLPCKTELWYNIDGCL
Keiboba_Pias-2  CSYHSVNIFKMKPLGLDDSAKLFFNRVFGSEQQCPYELSEVSYRITAKCGGLPLAVIIIAGLLASLPCKTELWYNIDGCL
IRIS_313-10738  CSYHSVNIFKMIPLGLDDSAKLFFNRVFGSEQQCPYELNEVSYRITAKCGGLPLAVIIIAGLLASLPCKTELWYNIDGCL
IRIS_313-11127  CSYHSVNIFKMIPLGLDDSAKLFFNRVFGSEQQCPYELNEVSYRITAKCGGLPLAVIIIAGLLASLPCKTELWYNIDGCL
IRIS_313-10841  CSYHSVNIFKMIPLGLDDSAKLFFNRVFGSEQQCPYELNEVSYRITAKCGGLPLAVIIIAGLLASLPCKTELWYNIDGCL
Azucena         CSYHSVNIFKMIPLGLDDSAKLFFNRVFGSEQQCPYELNEVSYRITAKCGGLPLAVIIIAGLLASLPCKTELWYNIDGCL
Nipponbare      CSYHSVNIFKMIPLGLDDSAKLFFNRVFGSEQQCPYELNEVSYRITAKCGGLPLAVIIIAGLLASLPCKTELWYNIDGCL
IRIS_313-7914   CSYHSVNIFKMKPLGLDDSAKLFFNRVFGSEQQCPYELNEVSYRITAKCGGLPLAVIIIAGLLASLPCKTELWYNIDGCL
IRIS_313-11708  CSYHSVNIFKMKPLGLDDSAKLFFNRVFGSEQQCPYELNEVSYRITAKCGGLPLAVIIIAGLLASLPCKTELWYNIDGCL
```

```
                      410       420       430       440       450       460       470       480
IRIS_313-11163  CSSVTTDIDLDEILKEIISLGYDNLPHYLKTCLLYLSLYSEGFIIWTADLLKQWISEGFIAVIDGEDIEEVAESYFYNLV
IRIS_313-10879  CSSVTTDIDLDEILKEIISLGYDNLPHYLKTCLLYLSLYSEGFIIWTADLLKQWISEGFIAVIDGEDIEEVAESYFYNLV
IRIS_313-8554   CSSVTTDIDLDEILKEIISLGYDNLPHYLKTCLLYLSLYSEGFIIWTADLLKQWISEGFIAVIDGEDIEEVAESYFYNLV
IRIS_313-12029  CSSVTTDIDLDEILKEIISLGYDNLPHYLKTCLLYLSLYSEGFIIWTADLLKQWISEGFIAVIDGEDIEEVAESYFYNLV
Keiboba_Pias-2  CSSVTTDIDLDEILKEIISLGYDNLPHYLKTCLLYLSLYSEGFIIWTADLLKQWISEGFIAVIDGEDIEEVAESYFYNLV
IRIS_313-10738  CSSVTTDIDLDEILKEIISLGYDNLPHYLKTCLLYLSLYSEGFIIWTADLLKQWISEGFIAVIDGEDIEEVAESYFYNLV
IRIS_313-11127  CSSVTTDIDLDEILKEIISLGYDNLPHYLKTCLLYLSLYSEGFIIWTADLLKQWISEGFIAVIDGEDIEEVAESYFYNLV
IRIS_313-10841  CSSVTTDIDLDEILKEIISLGYDNLPHYLKTCLLYLSLYSEGFIIWTADLLKQWISEGFIAVIDGEDIEEVAESYFYNLV
Azucena         CSSVTTDIDLDEILKEIISLGYDNLPHYLKTCLLYLSLYSEGFIIWTADLLKQWISEGFIAVIDGEDIEEVAESYFYNLV
Nipponbare      CSSVTTDIDLDEILKEIISLGYDNLPHYLKTCLLYLSLYSEGFIIWTADLLKQWISEGFIAVIDGEDIEEVAESYFYNLV
IRIS_313-7914   CSSVTTDIDLDEILKEIISLGYDNLPHYLKTCLLYLSLYSEGFIIWTADLLKQWISEGFIAVIDGEDIEEVAESYFYNLV
IRIS_313-11708  CSSVTTDIDLDEILKEIISLGYDNLPHYLKTCLLYLSLYSEGFIIWTADLLKQWISEGFIAVIDGEDIEEVAESYFYNLV
```

```
                      490       500       510       520       530       540       550       560
IRIS_313-11163  NRGMIQSVKTKYNNQVLCTVHHTVFDLIIHKSKEEKFISAIDYSQTMPGNSLEARRLSFHFSNTRYATEVAGITLSQVRS
IRIS_313-10879  NRGMIQSVKTKYNNQVLCTVHHTVFDLIIHKSKEEKFISAIDYSQTMPGNSLEARRLSFHFSNTRYATEVAGITLSQVRS
IRIS_313-8554   NRGMIQSVKTKYNNQVLCTVHHTVFDLIIHKSKEEKFISAIDYSQTMPGNSLEARRLSFHFSNTRYATEVAGITLSQVRS
IRIS_313-12029  NRGMIQSVKTKYNNQVLCTVHHTVFDLIIHKSKEEKFISAIDYSQTMPGNSLEARRLSFHFSNTRYATEVAGITLSQVRS
Keiboba_Pias-2  NRGMIQSVKTKYNNQVLCTVHHTVFDLIIHKSKEEKFISAIDYSQTMPGNSLEARRLSFHFSNTRYATEVAGITLSQVRS
IRIS_313-10738  NRGMIQSVKTKYNNQVLCTVHHTVFDLIIHKSKEEKFISAIDYSQTMPGNSLEARRLSFHFSNTRYATEVAGITLSQVRS
IRIS_313-11127  NRGMIQSVKTKYNNQVLCTVHHTVFDLIIHKSKEEKFISAIDYSQTMPGNSLEARRLSFHFSNTRYATEVAGITLSQVRS
IRIS_313-10841  NRGMIQSVKTKYNNQVLCTVHHTVFDLIIHKSKEEKFISAIDYSQTMPGNSLEARRLSFHFSNTRYATEVAGITLSQVRS
Azucena         NRGMIQSVKTKYNNQVLCTVHHTVFDLIIHKSKEEKFISAIDYSQTMPGNSLEARRLSFHFSNTRYATEVAGITLSQVRS
Nipponbare      NRGMIQSVKTKYNNQVLCTVHHTVFDLIIHKSKEEKFISAIDYSQTMPGNSLEARRLSFHFSNTRYATEVAGITLSQVRS
IRIS_313-7914   NRGMIQSVKTKYNNQVLCTVHHTVFDLIIHKSKEEKFISAIDYSQTMPGNSLEARRLSFHFSNTRYATEVAGITLSQVRS
IRIS_313-11708  NRGMIQSVKTKYNNQVLCTVHHTIFDLIIHKSKEEKFISAIDYSQTMPGNSLEARRLSFHFSNTRYATEVAGITLSQVRS
```

```
                      570       580       590       600       610       620       630       640
IRIS_313-11163  FAFLGLLKCMPSIMEFKLLRVLILEFWGDNHGCMSFNVARICRLFQLRYLKISSQIIIELPAQIRGLKYLETLEIDARVT
IRIS_313-10879  FAFLGLLKCMPSIMEFKLLRVLILEFWGDNHGCMSFNVARICRLFQLRYLKISSQIIIELPAQIRGLKYLETLEIDARVT
IRIS_313-8554   FAFLGLLKCMPSIMEFKLLRVLILEFWGDNHGCMSFNVARICRLFQLRYLKISSQIIIELPAQIRGLKYLETLEIDARVT
IRIS_313-12029  FAFLGLLKCMPSIMEFKLLRVLILEFWGDNHGCMSFNVARICRLFQLRYLKISSQIIIELPAQIRGLKYLETLEIDARVT
Keiboba_Pias-2  FAFLGLLKCMPSIMEFKLLRVLILEFWGDNHGCMSFNVARICRLFQLRYLKISSQIIIELPAQIRGLKYLETLEIDARVT
IRIS_313-10738  FAFLGLLKCMPSIMEFKLLRVLILEFWGDNHGCMSFNVARICRLFQLRYLKISSQIIIELPAQIRGLKYLETLEIDARVT
IRIS_313-11127  FAFLGLLKCMPSIMEFKLLRVLILEFWGDNHGCMSFNVARICRLFQLRYLKISSQIIIELPAQIRGLKYLETLEIDARVT
IRIS_313-10841  FAFLGLLKCMPSIMEFKLLRVLILEFWGDNHGCMSFNVARICRLFQLRYLKISSQIIIELPAQIRGLKYLETLEIDARVT
Azucena         FAFLGLLKCMPSIMEFKLLRVLILEFWGDNHGCMSFNVARICRLFQLRYLKISSQIIIELPAQIRGLKYLETLEIDARVT
Nipponbare      FAFLGLLKCMPSIMEFKLLRVLILEFWGDNHGCMSFNVARICRLFQLRYLKISSQIIIELPAQIRGLKYLETLEIDARVT
IRIS_313-7914   FAFLGLLKCMPSIMEFKLLRVLILEFWGDNHGCMSFNVAKICRLFQLRYLKISSQIIIELPAQIRGLKYLETLEIDARVT
IRIS_313-11708  FVFLGLLKCMPSIMEFKLLRVLILEFWGDNHGCMSFNVAKICRLFQLRYLKISSQIIIELPAQIGGLKYLETLEIDARVT
```

```
                      650       660       670       680       690       700       710       720
IRIS_313-11163  AVPSDIIHLRSLLHLYFQDGIVLPDGIGCIRSLRTLKYFDLGSNSEENIRSLGQLTNLRDLHLTCSAPKSNQQAKRNLVI
IRIS_313-10879  AVPSDIIHLRSLLHLYFQDGIVLPDGIGCIRSLRTLKYFDLGSNSEENIRSLGQLTNLRDLHLTCSAPKSNQQAKRNLVI
IRIS_313-8554   AVPSDIIHLRSLLHLYFQDGIVLPDGIGCIRSLRTLKYFDLGSNSEENIRSLGQLTNLRDLHLTCSAPKSNQQAKRNLVI
IRIS_313-12029  AVPSDIIHLRSLLHLYFQDGIVLPDGIGCIRSLRTLKYFDLGSNSEENIRSLGQLTNLRDLHLTCSAPKSNQQAKRNLVI
Keiboba_Pias-2  AVPSDIIHLRSLLHLYFQDGIVLPDGIGCIRSLRTLKYFDLGSNSEENIRSLGQLTNLRDLHLTCSAPKSNQQAKRNLVI
IRIS_313-10738  AIPSDIIHLRSLLHLYFQDGIVLPDGIGCIRSLRTLKYFDLGSNSEENIRSLGQLTNLRDLHLTCSAPKSNQQAKRNLVI
IRIS_313-11127  AIPSDIIHLRSLLHLYFQDGIVLPDGIGCIRSLRTLKYFDLGSNSEENIRSLGQLTNLRDLHLTCSAPKSNQQAKRNLVI
IRIS_313-10841  AIPSDIIHLRSLLHLYFQDGIVLPDGIGCIRSLRTLKYFDLGSNSEENIRSLGQLTNLRDLHLTCSAPKSNQQAKRNLVI
Azucena         AIPSDIIHLRSLLHLYFQDGIVLPDGIGCIRSLRTLKYFDLGSNSEENIRSLGQLTNLRDLHLTCSAPKSNQQAKRNLVI
Nipponbare      AIPSDIIHLRSLLHLYFQDGIVLPDGIGCIRSLRTLKYFDLGSNSEENIRSLGQLTNLRDLHLTCSAPKSNQQAKRNLVI
IRIS_313-7914   AVPSDIIHLRSLLHLYFQDGIVLPDGIGCIRSLRTLKYFDLGSNSEENIRSLGQLTNLRDLHLTCSAPKSNQQAKRNLVI
IRIS_313-11708  AVPSDIIHLRSLLHLYFQDGIVLPDGIGCIRSLRTLKYFDLGSNSEENIRSLGQLTNLRDLHLTCSAPKSNQQAKRNLVI
```

```
                      730       740       750       760       770       780       790       800
IRIS_313-11163  LASYTGKLGNLKSVKFSPGDSGMDISFLFYGIGISVDRSRTASSLPVSVRKLELPSICIFARLPDWIGQLRKLHTLNLAV
IRIS_313-10879  LASYTGKLGNLKSVKFSPGDSGMDISFLFYGIGISVDRSRTASSLPVSVRTLELPSICIFARLPDWIGQLRKLHTLNLAV
IRIS_313-8554   LASYTGKLGNLKSVKFSPGDSGMDISFLFYGIGISVDRSRTASSLPVSVRTLELPSICIFARLPDWIGQLRKLHTLNLAV
IRIS_313-12029  LASYTGKLGNLKSVKFSPGDSGMDISFLFYGIGISVDRSRTASSLPVSVRTLELPSICIFARLPDWIGQLRKLHTLNLAV
Keiboba_Pias-2  LASYTGKLGNLKSVKFSPGDSGMDISFLFYGIGISVDRSRTASSLPVSVRTLELPSICIFARLPDWIGQLRKLHTLNLAV
IRIS_313-10738  LASYTGKLGNLKSVKFSPGDSGMDISFLFYGIGISVDRSRTASSLPVSVRTLELPSICIFARLPDWIGQLRKLHTLNLAV
IRIS_313-11127  LASYTGKLGNLKSVKFSPGDSGMDISFLFYGIGISVDRSRTASSLPVSVRTLELPSICIFARLPDWIGQLRKLHTLNLAV
IRIS_313-10841  LASYTGKLGNLKSVKFSPGDSGMDISFLFYGIGISVDRSRTASSLPVSVRTLELPSICIFARLPDWIGQLRKLHTLNLAV
Azucena         LASYTGKLGNLKSVKFSPGDSGMDISFLFYGIGISVDRSRTASSLPVSVRTLELPSICIFARLPDWIGQLRKLHTLNLAV
Nipponbare      LASYTGKLGNLKSVKFSPGDSGMDISFLFYGIGISVDRSRTASSLPFSVRTLELPSICIFARLPDWIGQLRKLHTLNLAV
IRIS_313-7914   LASYTGKLGNLKSVKFSPGDSGMDISFLFYGIGISVDRSRTASSLPVSVRTLELPSICIFARLPDWIGQLRKLHTLNLAV
IRIS_313-11708  LASYTGKLGNLKSVKFSPGDSGMDISFLFYGIGISVDRSRTASSLPVSVRTLELPSICIFARLPDWIGQLRKLHTLNLAV
```

```
                      810       820       830       840       850       860       870       880
IRIS_313-11163  RELIENDIDSLAGLPDLIVLSMHIMKAPMERIVFNRKAFPVLKYFKFICGTLRMAFQAGAMANLHRLKLGFNAHKGEKYD
IRIS_313-10879  RELIENDIDSLAGLPDLIVLSMHIMKAPMERIVFNRKAFPVLKYFKFICGTLRMAFQAGAMANLHRLKLGFNAHKGEKYD
IRIS_313-8554   RELIENDIDSLAGLPDLIVLSMHIMKAPMERIVFNRKAFPVLKYFKFICGTLRMAFQAGAMANLHRLKLGFNAHKGEKYD
IRIS_313-12029  RELIENDIDSLAGLPDLIVLSMHIMKAPMERIVFNRKAFPVLKYFKFICGTLRMAFQAGAMANLHRLKLGFNAHKGEKYD
Keiboba_Pias-2  RELIENDIDSLAGLPDLIVLSMHIMKAPMERIVFNRKAFPVLKYFKFICGTLRMAFQAGAMANLHRLKLGFNAHKGEKYD
IRIS_313-10738  RELIENDIDSLAGLPDLIVLSMHIMKAPMERIVFNRKAFPVLKYFKFICGTLRMAFQAGAMANLHRLKLGFNAHKGEKYD
IRIS_313-11127  RELIENDIDSLAGLPDLIVLSMHIMKAPMERIVFNRKAFPVLKYFKFICGTLRMAFQAGAMANLHRLKLGFNAHKGEKYD
IRIS_313-10841  RELIENDIDSLAGLPDLIVLSMHIMKAPMERIVFNRKAFPVLKYFKFICGTLRMAFQAGAMANLHRLKLGFNAHKGEKYD
Azucena         RELIENDIDSLAGLPDLIVLSMHIMKAPMERIVFNRKAFPVLKYFKFICGTLRMAFQAGAMANLHRLKLGFNAHKGEKYD
Nipponbare      RELIENDIDSLAGLPDLIVLSMHIMKAPMERIVFNRKAFPVLKYFKFICGTLRMAFQAGAMANLHRLKLGFNAHKGEKYD
IRIS_313-7914   RELIENDIDSLAGLPDLIVLSMHVMKAPMERIVFNRKAFPVLKYFKFICGTLRMAFQAGAMANLHRLKLGFNAHKGEKYD
IRIS_313-11708  RELIENDIDSLAGLPDLIVLSMHVMKAPMERIVFNRKAFPVLKYFKFICGTLRMAFQAGAMANLHRLKLGFNAHKGEKYD
```

```
                      890       900       910       920       930       940       950       960
IRIS_313-11163  NILVGIEHLLNLKKIAVRIGGAAEAKESDRMAAEAALKEAIRKHLMFLDDLDIARVECVKEEYKCIKKKHKIKIEDSISE
IRIS_313-10879  NILVGIEHLLNLKKIAVRIGGAAEAKESDRMAAEAALKEAIRKHLMFLDDLDIARVECVKEEYKCIKKKHKIKIEDSISE
IRIS_313-8554   NILVGIEHLLNLKKIAVRIGGAAEAKESDRMAAEAALKEAIRKHLMFLDDLDIARVECVKEEYKCIKKKHKIKIEDSISE
IRIS_313-12029  NILVGIEHLLNLKKIAVRIGGAAEAKESDRMAAEAALKEAIRKHLMFLDDLDIARVECVKEEYKCIKKKHKIKIEDSISE
Keiboba_Pias-2  NILVGIEHLLNLKKIAVRIGGAAEAKESDRMAAEAALKEAIRKHLMFLDDLDIARVECVKEEYKCIKKKHKIKIEDSISE
IRIS_313-10738  NILVGIEHLLNLKKIAVRIGGAAEAKESDRMAAEAALKEAIRKHLMFLDDLDIARVECVKEEYKCIKKKHKIKIEDSISE
IRIS_313-11127  NILVGIEHLLNLKKIAVRIGGAAEAKESDRMAAEAALKEAIRKHLMFLDDLDIARVECVKEEYKCIKKKHKIKIEDSISE
IRIS_313-10841  NILVGIEHLLNLKKIAVRIGGAAEAKESDRMAAEAALKEAIRKHLMFLDDLDIARVECVKEEYKCIKKKHKIKIEDSISE
Azucena         NILVGIEHLLNLKKIAVRIGGAAEAKESDRMAAEAALKEAIRKHLMFLDDLDIARVECVKEEYKCIKKKHKIKIEDSISE
Nipponbare      NILVGIEHLLNLKKIAVRIGGAAEAKESDRMAAEAALKEAIRKHLMFLDDLDIARVECVKEEYKCIKKKHKIKIEDSISE
IRIS_313-7914   NILVGIEHLLNLKKIAVRIGGAAEAKESDRMAAEAALKEAIRKHLMFLDDLDIARVECVKEEYKCIKKKHKIKIEDSISE
IRIS_313-11708  NILVGIEHLLNLKKIAVRIGGAAEAKESDRMAAEAALKEAIRKHLMFLDDLDIARVECVKEEYKCIKKKHKIKIEDSISE
```

```
                      970       980       990      1000      1010      1020      1030      1040
IRIS_313-11163  KNGDSKKQHSVEKKAVWGKTMKNIADSGVFPEDYTMSREQRVAEGFVVGIEKCRAEDAAERIIRNVPVDYDGLGQVSTSK
IRIS_313-10879  KNGDSKKQHSVEKKAVWGKTMKNIADSGVFPEDYTMSREQRVAEGFVVGIEKCRAEDAAERIIRNVPVDYDGLGQVSTSK
IRIS_313-8554   KNGDSKKQHSVEKKAVWGKTMKNIADSGVFPEDYTMSREQRVAEGFVVGIEKCRAEDAAERIIRNVPVDYDGLGQVSTSK
IRIS_313-12029  KNGDSKKQHSVEKKAVWGKTMKNIADSGVFPEDYTMSREQRVAEGFVVGIEKCRAEDAAERIIRNVPVDYDGLGQVSTSK
Keiboba_Pias-2  KNGDSKKQHSVEKKAVWGKTMKNIADSGVFPEDYTMSREQRVAEGFVVGIEKCRAEDAAERIIRNVPVDYDGLGQVSTSK
IRIS_313-10738  KNGDSKKQHSVEKKAVWGKTMKNIADSGVFPEDYTMSREQRVAEGFVVGIEKCRAEDAAERIIRNVPVDYDGLGQVSTSK
IRIS_313-11127  KNGDSKKQHSVEKKAVWGKTMKNIADSGVFPEDYTMSREQRVAEGFVVGIEKCRAEDAAERIIRNVPVDYDGLGQVSTSK
IRIS_313-10841  KNGDSKKQHSVEKKAVWGKTMKNIADSGVFPEDYTMSREQRVAEGFVVGIEKCRAEDAAERIIRNVPVDYDGLGQVSTSK
Azucena         KNGDSKKQHSVEKKAVWGKTMKNIADSGVFPEDYTMSREQRVAEGFVVGIEKCRAEDAAERIIRNVPVDYDGLGQVSTSK
Nipponbare      KNGDSKKQHSVEKKAVWGKTMKNIADSGVFPEDYTMSREQRVAEGFVVGIEKCRAEDAAERIIRNVPVDYDGLGQVSTSK
IRIS_313-7914   KNGDSKKQHSVEKKAVWGKTMKNIADSGVFREDYTMSREQRVAEGFVVGIEKCRAEDAAERIIRNVPVDYDGLGQVSTSK
IRIS_313-11708  KNGDSKKQHSVEKKAVWGKTMKNIADSGVFPEDYTMSREQRVAEGFVVGIEKCRAEDAAERIIRNVPVDYDGLGQVSTSK
```

```
                     1050      1060      1070      1080      1090      1100      1110      1120
IRIS_313-11163  IQDHLPELAPRAVQNEKFGSSNDLSIMIQINKYARLPSYEWRDTDISKLNFRLLRAPMLLEAVTARCHLLDLILIGSNNI
IRIS_313-10879  IQDHLPELAPRAVQNEKFGSSNDLSIMIQINKYARLPSYEWRDTDISKLNFRLLRAPMLLEAVTARCHLLDLILIGSNNI
IRIS_313-8554   IQDHLPELAPRAVQNEKFGSSNDLSIMIQINKYARLPSYEWRDTDISKLNFRLLRAPMLLEAVTARCHLLDLILIGSNNI
IRIS_313-12029  IQDHLPELAPRAVQNEKFGSSNDLSIMIQINKYARLPSYEWRDTDISKLNFRLLRAPMLLEAVTARCHLLDLILIGSNNI
Keiboba_Pias-2  IQDHLPELAPRAVQNEKFGSSNDLSIMIQINKYARLPSYEWRDTDISKLNFRLLRAPMLLEAVTARCHLLDLILIGSNNI
IRIS_313-10738  IQDHLPELAPRAVQNEKFGSSNDLSIMIQINKYARLPSYEWRDTDISKLNFRLLRAPMLLEAVTARCHLLDLILIGSNNI
IRIS_313-11127  IQDHLPELAPRAVQNEKFGSSNDLSIMIQINKYARLPSYEWRDTDISKLNFRLLRAPMLLEAVTARCHLLDLILIGSNNI
IRIS_313-10841  IQDHLPELAPRAVQNEKFGSSNDLSIMIQINKYARLPSYEWRDTDISKLNFRLLRAPMLLEAVTARCHLLDLILIGSNNI
Azucena         IQDHLPELAPRAVQNEKFGSSNDLSIMIQINKYARLPSYEWRDTDISKLNFRLLRAPMLLEAVTARCHLLDLILIGSNNI
Nipponbare      IQDHLPELAPRAVQNEKFGSSNDLSIMIQINKYARLPSYEWRDTDISKLNFRLLRAPMLLEAVTARCHLLDLILIGSNNI
IRIS_313-7914   IQDHLPELAPRAVQNEKFGSSNDLSIMIQINKYARLPSYEWRDTDISKLNFRLLRAPMLLEAVTARCHLLDLILIGSNNI
IRIS_313-11708  IQDHLPELAPRAVQNEKFGSSNDLSIMIQINKYARLPSYEWRDTDISKLNFRLLRAPMLLEAVTARCHLLDLILIGSNNI
```

```
                     1130      1140      1150      1160      1170      1180      1190      1200
IRIS_313-11163  TVLDLGRPTITKLPASIECLPNLRYLRLQGTQLKSLSEVIVKMPTIRGLDIKNTKTEELPQGILRMKKLSHLSMGEKQKN
IRIS_313-10879  TVLDLGRPTITKLPASIECLPNLRYLRLQGTQLKSLSEVIVKMPTIRGLDIKNTKTEELPQGILRMKKLSHLSMGEKQKN
IRIS_313-8554   TVLDLGRPTITKLPASIECLPNLRYLRLQGTQLKSLSEVIVKMPTIRGLDIKNTKTEELPQGILRMKKLSHLSMGEKQKN
IRIS_313-12029  TVLDLGRPTITKLPASIECLPNLRYLRLQGTQLKSLSEVIVKMPTIRGLDIKNTKTEELPQGILRMKKLSHLSMGEKQKN
Keiboba_Pias-2  TVLDLGRPTITKLPASIECLPNLRYLRLQGTQLKSLSEVIVKMPTIRGLDIKNTKTEELPQGILRMKKLSHLSMGEKQKN
IRIS_313-10738  TVLDLGRPTITKLPASIECLPNLRYLRLQGTQLKSLSEVIVKMPTIRGLDIKNTKTEELPQGILRMKKLSHLSMGEKQKN
IRIS_313-11127  TVLDLGRPTITKLPASIECLPNLRYLRLQGTQLKSLSEVIVKMPTIRGLDIKNTKTEELPQGILRMKKLSHLSMGEKQKN
IRIS_313-10841  TVLDLGRPTITKLPASIECLPNLRYLRLQGTQLKSLSEVIVKMPTIRGLDIKNTKTEELPQGILRMKKLSHLSMGEKQKN
Azucena         TVLDLGRPTITKLPASIECLPNLRYLRLQGTQLKSLSEVIVKMPTIRGLDIKNTKTEELPQGILRMKKLSHLSMGEKQKN
Nipponbare      TVLDLGRPTITKLPASIECLPNLRYLRLQGTQLKSLSEVIVKMPTIRGLDIKNTKTEELPQGILRMKKLSHLSMGEKQKN
IRIS_313-7914   TVLDLGRPTITKLPASIECLPNLRYLRLRGTQLKSLSEVIVKMPTIRGLDIKNTKTEELPQGILRMKKLSHLSMGEKQKN
IRIS_313-11708  TVLDLGRPTITKLPASIECLPNLRYLRLRGTQLKSLSEVIVKMPTIRGLDIKNTKTEELPQGILRMKKLSHLSMGEKQKN
```

```
                         1210      1220      1230      1240      1250      1260      1270      1280
IRIS_313-11163  IQVFMEKMQTLAETVQDSDDLSDETEGIADDDEGEFSTRANASTPKVDEDEVDRRANNFIARFRKQITIRNSGFPKRRAA
IRIS_313-10879  IQVFMEKMQTLAETVQDSDDLSDETEGIADDDEGEFSTRANASTPKVDEDEVDRRANNFIARFRKQITIRNSGFPKRRAA
IRIS_313-8554   IQVFMEKMQTLAETVQDSDDLSDETEGIADDDEGEFSTRANASTPKVDEDEVDRRANNFIARFRKQITIRNSGFPKRRAA
IRIS_313-12029  IQVFMEKMQTLAETVQDSDDLSDETEGIADDDEGEFSTRANASTPKVDEDEVDRRANNFIARFRKQITIRNSGFPKRRAA
Keiboba_Pias-2  IQVFMEKMQTLAETVQDSDDLSDETEGIADDDEGEFSTRANASTPKVDEDEVDRRANNFIARFRKQITIRNSGFPKRRAA
IRIS_313-10738  IQVFMEKMQTLAETVQDSDDLSDETEGIAD.DEGEFSTRANASTPKVDEDEVDRRANNFIARFRKQITIRNSGFAKKESS
IRIS_313-11127  IQVFMEKMQTLAETVQDSDDLSDETEGIAD.DEGEFSTRANASTPKVDEDEVDRRANNFIARFRKQITIRNSGFAKKESS
IRIS_313-10841  IQVFMEKMQTLAETVQDSDDLSDETEGIAD.DEGEFSTRANASTPKVDEDEVDRRANNFIARFRKQITIRNSGFAKKESS
Azucena         IQVFMEKMQTLAETVQDSDDLSDETEGIAD.DEGEFSTRANASTPKVDEDEVDRRANNFIARFRKQITIRNSGFAKKESS
Nipponbare      IQVFMEKMQTLAETVQDSDDLSDETEGIAD.DEGEFSTRANASTPKVDEDEVDRRANNFIARFRKQITIRNSGFAKKESS
IRIS_313-7914   IQVFMEKMQTLAETVQDSDDLSDETEGIADDDEGEFSTRANASTPKVDEDEVDRRANNFIARFRKQITIRNSGFAKKESS
IRIS_313-11708  IQVFMEKMQTLAETVQDSDDLSDETEGIADDDEGEFSTRANAPTPKVDEDEVDRRANNFIARFRKQITIRNSGFAKKESS

                         1290      1300      1310      1320      1330      1340      1350      1360
IRIS_313-11163  STNDCGYEISMSANSPREVDDFKKKFDEIIARNRHTWKPIESPRSVKHGKYFVRCPPSLITSAVFSFFFLSIFLVWYSLR
IRIS_313-10879  STNDCGYEISMSANSPREVDDFKKKFDEIIARNRHTWKPIESPRSVKHGKYFVRCPPSLITSAVFSFFFLSIFLVWYSLR
IRIS_313-8554   STNDCGYEISMSANSPREVDDFKKKFDEIIARNRHTWKPIESPRSVKHGKYFVRCPPSLITSAVFSFFFLSIFLVWYSLR
IRIS_313-12029  STNDCGYEISMSANSPREVDDFKKKFDEIIARNRHTWKPIESPRSVKHGKYFVRCPPSLITSAVFSFFFLSIFLVWYSLR
Keiboba_Pias-2  STNDCGHEISMSANSPREVDDFKKKFDEIIARNRHTWKPIESPRSVKHGKYFVRCPPSLITSAVFSFFFLSIFLVWYSLR
IRIS_313-10738  IDERLWIRDLDECQ....LSKRGGRF......................................................
IRIS_313-11127  IDERLWIRDLDECQ....LSKRGGRF......................................................
IRIS_313-10841  IDERLWIRDLDECQ....LSKRGGRF......................................................
Azucena         IDERLWIRDLDECQ....LSKRGGRF......................................................
Nipponbare      IDERLWIRDLDECQ....LSKRGGRF......................................................
IRIS_313-7914   IDERLWIRDLDECQ....LSKRGGRF......................................................
IRIS_313-11708  IDKRLWIRDLDECQ....LSKRGGRF......................................................

IRIS_313-11163  FRILSGKP
IRIS_313-10879  FRILSGKP
IRIS_313-8554   FRILSGKP
IRIS_313-12029  FRILSGKP
Keiboba_Pias-2  FRILSGKP
IRIS_313-10738  ........
IRIS_313-11127  ........
IRIS_313-10841  ........
Azucena         ........
Nipponbare      ........
IRIS_313-7914   ........
IRIS_313-11708  ........
```

Supplementary Figure 12. Pias-2/LOC_Os11g11810 amino acid alignment. Where homology was detected to LOC_Os11g11810, amino acid sequence was extracted from any of the 10 accessions sequenced in this article, plus Azucena, Keiboba, Nipponbare, and 12 other diverse rice genomes. Where accessions did not containP ias-2/LOC_Os11g11810, the *RGA5* gene was observed and the protein sequence encoded by this gene can be found in a separate supplementary figure.

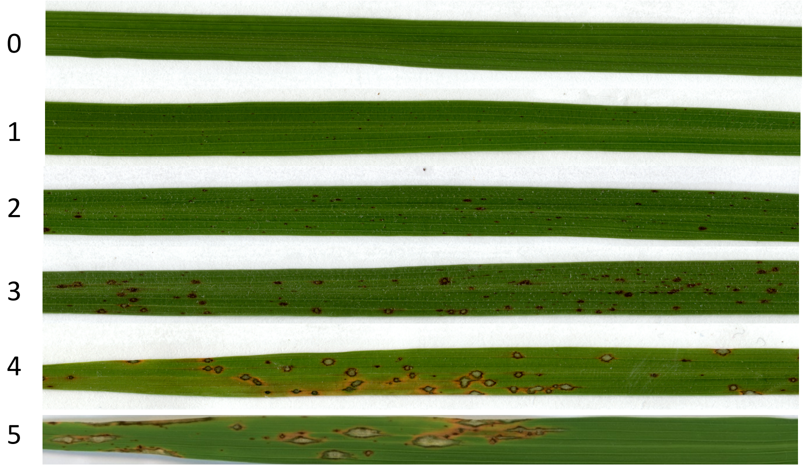

0. No macroscopic sign of infection

1. Small black dots

2. Large black spots – no tan centers

3. Open round lesions with small tan centers

4. Large round and elliptical lesions spanning leaf veins

5. Large elliptical lesions spanning leaf veins and coalescing

Supplementary Figure 13. Representative examples of disease severity scores used in this study. Leaves presented represent typical examples of phenotypes observed seven days post infection. Where no macroscopic signs of infection are observed, susceptible controls must carry expected lesion phenotypes after infection with the same inoculum to be sure that a viable infection took place. Lesion scores are based on the presence of the most severe lesion type as variation can exist across the leaf, particularly when severe infection occurs due to high levels of inoculum. The representative infection scores are based on the evaluation system published by JIRCAS (Hayashi, et al, 2009).

Supplementary Table 1. Summary of infection data used for each *M. oryzae* isolate infection. For each of the six inoculation tests performed, the number of rice accessions which had consistent disease severity scores (SD <1.3) are indicated in bold. Data collected from accessions meeting this criterion were used for GWAS. Input data and peak association information can be found in supplementary file 2 for C923.49, and supplementary file 1 for all other isolates.

***M. oryzae* isolate**	**Mo15-23**	**Mo15-24**	**M64-1-3-9-1**	**IK81-25**	**Mo15-125**	**C923-49**
Number of accessions with one or more biological replicates	480	476	458	494	479	490
**Number of accessions with disease severity SD <1.3**	**404**	**416**	**390**	**442**	**425**	**440**
Accessions with 10 biological replicates	233	292	400	195	421	304
Accessions with 9 biological replicates	36	21	20	45	15	28
Accessions with 8 biological replicates	60	38	17	80	12	28
Accessions with 7 biological replicates	55	44	11	59	4	32
Accessions with 6 biological replicates	49	35	7	47	8	41
Accessions with 5 biological replicates	27	10	6	43	5	24
Accessions with 4 biological replicates	13	19	9	9	2	13
Accessions with 3 biological replicates	5	10	2	7	2	10
Accessions with 2 biological replicates	4	5	3	6	8	7
Accessions with 1 biological replicate	1	1	1	3	2	3

Supplementary Table 2. Proportion of susceptible accessions carrying peak associated SNPs. In general, accessions carrying peak resistance associated SNPs to *M. oryzae* isolates had disease severity scores <3, consistent with the strong GWAS associations detected. Mo15-125 and IK81-25 peak resistance associations were exceptions with 22.22% and 29.49% of accessions having a susceptible average lesion score (≥ 3).

Isolate	*Peak SNP association*	*Proportion of susceptible accessions carrying association*
**Mo15-125**	Chr 12 : 10,926,845 **(A)**	24/108 associated accessions have average lesion score ≥3 (22.22 %)
**M64-1-3-9-1**	Chr 12 : 10,926,845 **(A)**	23/78 associated accessions have average lesion score ≥3 (29.49 %)
**IK81-25**	Chr 12 : 10,830,098 **(T)**	2/98 associated accessions have average lesion score ≥3 (2.04 %)
**C923-49**	Chr 11 : 6,540,075 **(A)**	1/196 associated accessions has average lesion score ≥3 (1.02 %)
**Mo15-23**	Chr 12 : 10,797,768 **(T)**	5/82 associated accessions have average lesion score ≥3 (6.10 %)
**Mo15-24**	Chr 12 : 10,806,802 **(C)**	4/87 associated accessions have average lesion score ≥3 (4.60 %)

Supplementary Tables 3 a,b. Virulence spectra and *Avr* gene presence/absence of *M. oryzae* isolates used in this study. Isolates used for diversity panel screening and GWAS are shown in **bold**. Isolates not in bold were used for subsequent specificity analysis and validation experiments. Information kindly provided by the International Rice Research Institute (IRRI). **a)** Virulence to *Pia*, *Pita* and *Pita2/Ptr* are **underlined and in bold.** Virulence spectrum was determined by infecting near-isogenic rice lines containing introgressions of the resistance genes listed. Where an isolate is not shown to be virulent to a gene listed, an avirulence response was reported in the corresponding near isogenic line. **b)** *Avr* gene presence/absence was determined by PCR. The presence of *Avr-Pita* genes which contradicts virulence testing on near-isogenic lines are shown in **bold**. This contradiction is consistent with observations that *Ptr/Pita2* is required for resistance to *Avr-Pita containing isolates*.

a) Isolate	*Virulent to near-isogenic rice lines containing:*
Mo15-125	***Pia***, *Piks, Pik, Pikp, Pikh,* ***Pi-ta***, *Pib, Pit, Pi1, Pi7, Pi12, Pi19, Pikm, Pi20,* ***Pi-ta2/Ptr***, *Pi11*
M64-1-3-9-1	***Pia***, *Pii, Piks, Pik, Pikp, Pib, Pit, Pi1, Pi3, Pi12, Pi19, Pi20, Pi11*
IK81-25	***Pia***, *Piks, Pikp, Pikh, Pizt, Pib, Pit, Pi1, Pi7, Pi12, Pi19, Pikm, Pi20,* ***Pi-ta***, *Pi11*
C923-49	*Pii, Piks, Pit, Pish, Pi3, Pi5, Pi19,* ***Pi-ta***
Mo15-23	***Pia***, *Piks, Pik, Pikp, Pikh, Piz5, Pizt,* ***Pi-ta***, *Pib, Pit, Pi1, Pi7, Pi12, Pi19, Pikm, Pi11*
Mo15-24	***Pia***, *Pii, Piks, Pikp,* ***Pi-ta***, *Pib, Pit, Pi3, Pi5, Pi7, Pi12, Pi19, Pi11*
M101-1-2-9-1	***Pia***, *Pii, Piks, Piz5, Pizt,* ***Pi-ta***, *Pit, Pi3, Pi19, Pi20, Pi11*
Ca89	***Pia***, *Pii, Piks, Piz, Pizt,* ***Pi-ta***, *Pib, Pit, Pish, Pi3, Pi5, Pi12, Pi19, Pi20,* ***Pi-ta2/Ptr***, *Pi11*

b) Isolate	*Presence of Avr genes as determined by PCR:*
Mo15-125	AvrPiz-t, Avrpii, Avr-Pik, Avr-Pi9, **Avr-Pita**
M64-1-3-9-1	AvrPiz-t, Avr-Pita
IK81-25	Avr-Pi9, **Avr-Pita**
C923-49	AvrPiz-t, Avr-Pia, Avr-Pik, Avrpib, Avr-Pi9, **Avr-Pita**
Mo15-24	AvrPiz-t, Avr-Pik, Avr-Pi9
M101-1-2-9-1	Avr-Pik, Avr-Pib, Avr-Pi9, **Avr-Pita**
Ca89	Avr-Pik, Avr-Pi9

Supplementary Table 4. Linked read sequencing (10x Genomics, Inc) *de novo* assembly statistics.

Genome assembly statistics following assembly using the 10X Genomics Supernova - 2.1.1 software package for d*e novo* assembly.

Accession	Total sequence length	Number of scaffolds	Scaffold N50	Number of contigs	Contig N50	Fold Coverage
IRIS_313-12190	427,606,848	12,202	4,158,354	22,621	42,536	62.7425
IRIS_313-11127	424,149,202	11,576	4,571,059	21,764	43,459	63.3593
IRIS_313-11360	427,369,838	12,303	3,690,457	22,405	44,083	63.4671
IRIS_313-7914	412,462,330	11,525	3,715,755	22,146	41,065	63.3939
IRIS_313-8554	419,730,278	12,563	3,205,731	23,357	39,580	62.324
IRIS_313-10314	419,189,211	12,856	2,026,036	23,689	41,008	61.6208
IRIS_313-10059	502,613,927	31,755	773,075	44,898	29,682	63.6699
IRIS_313-10879	427,931,591	12,764	3,896,250	23,025	43,059	62.1701
IRIS_313-11786	411,001,814	10,313	2,314,655	19,112	56,607	62.9281
IRIS_313-10738	419,096,195	11,832	3,389,810	20,766	54,370	65.4986

Supplementary table 5. *Ptr$_a$* and *Ptr$_b$* resistance or susceptibility responses to *M. oryzae* diagnostic isolates. *Ptra* NIL CO39 contains an introgression region containing *Ptra* in the CO39 genetic background. This line was resistant to all isolates tested but Ca89. However, given this introgressed region may contain other resistance genes, infection testing was also performed using *ptra* mutants #377 and #6599 in the IR64 background, which was subsequently crossed to CO39. In the case of Mo15-24, M101-1-2-9-1, and IK81-25 infection testing, the *ptra* mutants were resistant, but this response may be due to lingering sources of resistance carried in the IR64 background. The *ptra* mutants showed susceptibility when infected with Mo15-23 and M64-1-3-9-1, while *Ptrb* transgenics (Ptrb CO39) only gain resistance to Mo15-23 when compared to the susceptible parent line CO39, or non-transgenic siblings. Virulence responses of the various isolates to the presence of *Ptra* and *Ptrb* are summarised in the final two rows of the table.

Genotype	Mo15-23	Mo15-24	M64-1-3-9-1	M101-1-2-9-1	IK81-25	Ca89
Ptr$_a$ NIL CO39	R	R	R	R	R	S
Ptr$_a$ IR64	R	R	R	R	R	S
ptr$_a$ #377	S	R	S	R	R	S
ptr$_a$ #6599	S	R	S	R	R	S
CO39	S	S	S	S	S	S
Ptr$_b$ CO39	R	R	S	S	S	S
**Virulence summary**						
Ptr$_a$	**R**	**?**	**R**	**?**	**?**	**S**
Ptr$_b$	**R**	**R**	**S**	**S**	**S**	**S**

Supplementary Table 6. Summary of PISA (Proteins, Interfaces, Structures and Assemblies) analysis of the model structures AVR1-CO39/HMA complexes. Details of total interface area (Å2), solvation free energy gain upon complex formation (DiG), number of H-Bonds and number of involved residues are given. For the reference Sasanishki RGA5 HMA-AVR1CO39 the values given between parenthesis correspond to the initial PDB structure 5ZNG. For **IRIS-313-11786** (*) the ASVNGVESMQ-loop sequence was modelled by RCD+ server and the complex was refined on GalaxyWEB server.

Accession	**No. of interacting residues in AVR1-CO39**	**No. of interacting residues in RGA5 HMA**	**Interface area Å^2**	**D^{i}G kcal/mol**	**No. of H-Bonds**
Sasanishki	12 (12)	16 (16)	495 (493)	-4.7 (-4.6)	8 (7)
IRIS_313-11786 *	17	18	617	-7.9	9
93-11	12	16	502	-4.4	7
CO39	12	16	491	-5.0	7
IRIS_313-12190	12	16	489	-5.1	8

Supplementary Table 7. Summary of Guy11 isolate infection testing on *Pia* variant containing rice accessions. Accessions are color coded to match the HMA domain sequence aligned in **Fig. 3 a** and modelled in **Fig. 3 b/c**. IRIS_313-11360* contains a frameshift mutation after the HMA domain of *Rga5.* Resistance/susceptibility responses in white are indicative of functional RGA4 and RGA5, responses in grey are indicative of non-functional RGA4 and RGA5, and responses in black are indicative of accessions carrying resistance to the Guy11 strain which prevents assessment of RGA4 and RGA5 function using transgenic Guy11 expressing AVR1-CO39 or AVR-Pia.

Accession	Guy11_EV	Guy11_AVR1-CO39	Guy11_AVR-Pia	Functional RGA4 and RGA5
Aichi Asahi	**S**	**R**	**R**	**yes**
CO39	**S**	**R**	**R**	**yes**
Kitaake	**S**	**R**	**R**	**yes**
IRIS_313-11786	**S**	**R**	**R**	**yes**
IRIS_313-12190	**S**	**R**	**R**	**yes**
Maratelli	**S**	**S**	**S**	**no**
Nipponbare	**S**	**S**	**S**	**no**
IRIS_313-11360*	**S**	**S**	**S**	**no**
IRIS_313-10314	**R**	**R**	**R**	**~**
IRIS_313-10059	**R**	**R**	**R**	**~**
