# Supplementary material for: Genome-wide association analysis uncovers rice blast resistance alleles of Ptr and Pia
Source: Commun Biol. 2024 May 20;7:607. doi: 10.1038/s42003-024-06244-z (PMC11106262; doi:10.1038/s42003-024-06244-z)
Supplement: Supplementary file 2 — Description of Additional Supplementary Materials [file 42003_2024_6244_MOESM2_ESM.pdf]

Description of Additional Supplementary Files

File name: Supplementary Data 1

Description: GWAS source data and tabulated results. Source data behind graphs in paper.

File name: Supplementary Data 2

Description: *Pia* locus association and 3K rice genomes allele profiling.

File name: Supplementary Data 3

Description: *Ptr* genomic multiple sequence alignment.

File name: Supplementary Data 4

Description: Summary of sequence verified Ptr and RGA5 protein variants.

File name: Supplementary Data 5

Description: *Ptr* allele profiling of 3K rice genomes.

File name: Supplementary Data 6

Description: Primers used in this study.

File name: Supplementary Data 7

Description: Extracted protein sequence used in multiple sequence alignments

File name: Supplementary Data 8

Description: Example code and instruction for performing GWAS with compatible GWAS data file
