# Supplementary material for: Genome-wide association analysis uncovers rice blast resistance alleles of Ptr and Pia
Source: Commun Biol. 2024 May 20;7:607. doi: 10.1038/s42003-024-06244-z (PMC11106262; doi:10.1038/s42003-024-06244-z)
Supplement: Supplementary file 10 — Supplementary Data 8 [file 42003_2024_6244_MOESM10_ESM.zip › Example GWAS R script.rtf]

#### EXAMPLE GWAS processing using Mo15.125 input data found in: ‘riceblast complete dataset.txt’, & ‘3K RG 1M GWAS SNP Dataset, all chromosomes’ from: https://snp-seek.irri.org/_download.zul;jsessionid=33CA68A42B5FEBF3CFC04089C4EE1C26############ Mo15.125_Clean  ###### ########### load data#convert.snp.tped("1Mtped.tped","1Mtped.tfam","rice_blast_feb2018.raw",strand="+") # .map and .ped files can be generated with vcftools: vcftools --vcf yourfile.vcf --plink --out your_output_file --recode#data_association_mapping_BLAST <- load.gwaa.data(phe="riceblast complete dataset.txt",gen="rice_blast_feb2018.raw",force=TRUE) # pheno file should not be space-delimited#ricedescriptives.marker(data_association_mapping_BLAST)dir.create(path = "Mo15.125_Clean") data_Mo15.125_Cleanbinary.qt <- qtscore(Mo15.125_Clean, data_association_mapping_BLAST, trait="binomial")descriptives.scan(data_Mo15.125_Cleanbinary.qt, sort="Pc1df")#'Mo15.125_Clean' designated as column contains binomial data (0,1), Alternatively Mo15.125 can be included. tiff("Mo15.125_Clean/lamba_qt_Mo15.125_Clean_NC.tiff", height = 32, width = 32, units = 'cm', compression = "lzw", res = 300)par(mfrow=c(2,1))estlambda(data_Mo15.125_Cleanbinary.qt[, "P1df"], plot=TRUE,main="NC (Mo15.125_Clean) p1df")  estlambda(data_Mo15.125_Cleanbinary.qt[, "Pc1df"], plot=TRUE,main="NC (Mo15.125_Clean) pc1df") # your estimated lambad should as close as possible to 1. This allows you assessing the best model (correction vs correction)dev.off()write.table(results(data_Mo15.125_Cleanbinary.qt),"Mo15.125_Clean/Mo15.125_CleanBinary_no_correction_two.txt")output_Mo15.125_Clean <- (results(data_Mo15.125_Cleanbinary.qt))ncol(output_Mo15.125_Clean)head(output_Mo15.125_Clean)output_Mo15.125_Clean_qqman <- output_Mo15.125_Clean[,c(1,2,15)]colnames(output_Mo15.125_Clean_qqman) <-c("CHR","BP","P")str(output_Mo15.125_Clean_qqman)output_Mo15.125_Clean_qqman$CHR <-as.numeric(as.character(output_Mo15.125_Clean_qqman$CHR))unique(output_Mo15.125_Clean_qqman$CHR)str(output_Mo15.125_Clean_qqman)Mo15.125_examination<- read.table("Mo15.125_Clean/Mo15.125_CleanBinary_no_correction_two.txt")write.table(Mo15.125_examination[order(Mo15.125_examination$Pc1df),c(1,2,4,5,15)],"Mo15.125_Clean/Mo15.125_top_snps_two.txt")Mo15.125_top_snps <- (Mo15.125_examination[order(Mo15.125_examination$Pc1df),c(1,2,4,5,15)])#write a table for top 1000snps sorted by chromosomeMo15.125_top_1000snps <- Mo15.125_top_snps[1:1000,]write.table(Mo15.125_top_1000snps[order(Mo15.125_top_1000snps$Chromosome),],"Mo15.125_Clean/Mo15.125_top_1000snps_two.txt")# manipulate plots with qq man:tiff("Mo15.125_Clean/Mo15.125_Clean_manhattan_NC.tiff", height = 16, width = 32, units = 'cm', compression = "lzw", res = 300)manhattan(output_Mo15.125_Clean_qqman, genomewideline = FALSE, suggestiveline = FALSE)dev.off()#pdf("Mo15.125_Clean/MHplot_Mo15.125_CleanBinary_no_correction.pdf") # store plot#manhattan(output_Mo15.125_Clean_qqman,main="Mo15.125_Clean", genomewideline = FALSE, suggestiveline = FALSE)#dev.off()#manhattan(subset(output_Mo15.125_Clean_qqman, CHR==11),main="Chr11" ,xaxt="n", genomewideline = FALSE, suggestiveline = FALSE) # remove axis labels#label_axis=c("0Mb","5Mb","10Mb","15Mb","20Mb","25Mb","30Mb") # store new axis labels in "label_axis"#axis(1, at=c(0,5000000,10000000,15000000,20000000,25000000,30000000), labels=label_axis) # display new axis labels#manhattan(subset(output_Mo15.125_Clean_qqman, CHR==3),main="Chr3", xaxt="n", genomewideline = FALSE, suggestiveline = FALSE)#label_axis=c("0Mb","5Mb","10Mb","15Mb","20Mb","25Mb","30Mb","35Mb") # store new axis labels in "label_axis"#axis(1, at=c(0,5000000,10000000,15000000,20000000,25000000,30000000,35000000), labels=label_axis) # display new axis labels#manhattan(subset(output_Mo15.125_Clean_qqman, CHR==4),main="Chr4", xaxt="n", genomewideline = FALSE, suggestiveline = FALSE)#label_axis=c("0Mb","5Mb","10Mb","15Mb","20Mb","25Mb","30Mb","35Mb") # store new axis labels in "label_axis"#axis(1, at=c(0,5000000,10000000,15000000,20000000,25000000,30000000,35000000), labels=label_axis) # display new axis labels#manhattan(subset(output_Mo15.125_Clean_qqman, CHR==8),main="Chr8", xaxt="n", genomewideline = FALSE, suggestiveline = FALSE)#label_axis=c("0Mb","5Mb","10Mb","15Mb","20Mb","25Mb","30Mb","35Mb") # store new axis labels in "label_axis"#axis(1, at=c(0,5000000,10000000,15000000,20000000,25000000,30000000,35000000), labels=label_axis) # display new axis labelstiff("Mo15.125_Clean/Mo15.125_Clean_manhattan_NC_CHR12.tiff", height = 16, width = 32, units = 'cm', compression = "lzw", res = 300)manhattan(subset(output_Mo15.125_Clean_qqman, CHR==12),main="Chr12", xaxt="n", genomewideline = FALSE, suggestiveline = FALSE)label_axis=c("0Mb","5Mb","10Mb","15Mb","20Mb","25Mb","30Mb","35Mb") # store new axis labels in "label_axis"axis(1, at=c(0,5000000,10000000,15000000,20000000,25000000,30000000,35000000), labels=label_axis) # display new axis labelsdev.off()###### Mo15.125_Clean - GWAS correction##### calculate kinship matrix# gkin <- ibs(data_association_mapping_BLAST[, autosomal(data_association_mapping_BLAST)], weight="freq") # full dataset#gkin <- ibs(data_association_mapping_BLAST,snps=sample(autosomal(data_association_mapping_BLAST),100000,replace=FALSE)) #subset of 100000 random snps#gkin <-read.table("gkin.txt")#gkin[1:5, 1:5]#data_association_mapping_BLAST.dist <- as.dist(0.5-gkin)#data_association_mapping_BLAST.mds <- cmdscale(data_association_mapping_BLAST.dist)#plot(data_association_mapping_BLAST.mds) # check for structure#dst <- as.dist(0.5-gkin)#pcs <- cmdscale(dst,k=10)#pcs[1:5,]# PCA correctiondata_Mo15.125_Cleanbinary.pca <- qtscore(Mo15.125_Clean~pcs[,1]+pcs[,2]+pcs[,3], data_association_mapping_BLAST, trait="binomial")tiff("Mo15.125_Clean/Mo15.125_Clean_lamba_qtPCA_riceblast.tiff", height = 32, width = 32, units = 'cm', compression = "lzw", res = 300)par(mfrow=c(2,1))estlambda(data_Mo15.125_Cleanbinary.pca[, "P1df"], plot=TRUE,main="pca (Mo15.125_Cleanbinary) p1df")estlambda(data_Mo15.125_Cleanbinary.pca[, "Pc1df"], plot=TRUE,main="pca (Mo15.125_Cleanbinary) pc1df")dev.off()write.table(results(data_Mo15.125_Cleanbinary.pca),"Mo15.125_Clean/Mo15.125_Clean_PCA_correction.txt")#plot(data_Mo15.125_Cleanbinary.pca)output_Mo15.125_Clean_pca <- (results(data_Mo15.125_Cleanbinary.pca))head(output_Mo15.125_Clean_pca)ncol(output_Mo15.125_Clean_pca)output_Mo15.125_Clean_qqman_pca <- output_Mo15.125_Clean_pca[,c(1,2,15)]colnames(output_Mo15.125_Clean_qqman_pca) <-c("CHR","BP","P")str(output_Mo15.125_Clean_qqman_pca)output_Mo15.125_Clean_qqman_pca$CHR <-as.numeric(as.character(output_Mo15.125_Clean_qqman_pca$CHR))unique(output_Mo15.125_Clean_qqman_pca$CHR)str(output_Mo15.125_Clean_qqman_pca)tiff("Mo15.125_Clean/Mo15.125_Clean_manhattan_PCA.tiff", height = 16, width = 32, units = 'cm', compression = "lzw", res = 300)manhattan(output_Mo15.125_Clean_qqman_pca, genomewideline = FALSE, suggestiveline = FALSE)dev.off()Mo15.125_examination_pca<- read.table("Mo15.125_Clean/Mo15.125_Clean_PCA_correction.txt")Mo15.125_top_snps_pca <- (Mo15.125_examination_pca[order(Mo15.125_examination_pca$Pc1df),c(1,2,4,5,15)])head(Mo15.125_top_snps_pca)#write a table for top 1000snps after PCA correction sorted by Chromosome and Pc1dfMo15.125_top_1000snps_pca <- Mo15.125_top_snps_pca[1:1000,]write.table(Mo15.125_top_1000snps_pca[order(Mo15.125_top_1000snps_pca$Chromosome, Mo15.125_top_1000snps_pca$Pc1df),],"Mo15.125_Clean/Mo15.125_Clean_PCA_correction_top_1000snps.txt")#######mixed model correction - Mixed model correction was found to be the most robust correction for population structure.######h2a <- polygenic(Mo15.125_Clean,data_association_mapping_BLAST,kin=gkin)h2a$esth2data_Mo15.125_Cleanbinary.mm <- mmscore(h2a,data_association_mapping_BLAST)tiff("Mo15.125_Clean/lamba_qtMM_BLASTMo15.125_Clean.tiff", height = 32, width = 32, units = 'cm', compression = "lzw", res = 300)par(mfrow=c(2,1))estlambda(data_Mo15.125_Cleanbinary.mm[, "P1df"], plot=TRUE,main="mm P1df")estlambda(data_Mo15.125_Cleanbinary.mm[, "Pc1df"], plot=TRUE,main="mm Pc1df")dev.off()#plot(data_Mo15.125_Cleanbinary.mm)#write.table(results(data_Mo15.125_Cleanbinary.mm),"Mo15.125_Clean/Mo15.125_Clean_mm_correction.txt")output_Mo15.125_Clean_mm <- (results(data_Mo15.125_Cleanbinary.mm))ncol(output_Mo15.125_Clean_mm)head(output_Mo15.125_Clean_mm)output_Mo15.125_Clean_qqman_mm <- output_Mo15.125_Clean_mm[,c(1,2,11)]head(output_Mo15.125_Clean_qqman_mm)colnames(output_Mo15.125_Clean_qqman_mm) <-c("CHR","BP","P")str(output_Mo15.125_Clean_qqman_mm)output_Mo15.125_Clean_qqman_mm$CHR <-as.numeric(as.character(output_Mo15.125_Clean_qqman_mm$CHR))unique(output_Mo15.125_Clean_qqman_mm$CHR)str(output_Mo15.125_Clean_qqman_mm)tiff("Mo15.125_Clean/Mo15.125_Clean_manhattan_mm.tiff", height = 16, width = 32, units = 'cm', compression = "lzw", res = 300)manhattan(output_Mo15.125_Clean_qqman_mm, genomewideline = FALSE, suggestiveline = FALSE)dev.off()tiff("Mo15.125_Clean/Mo15.125_Clean_manhattan_mm_CHR12.tiff", height = 16, width = 32, units = 'cm', compression = "lzw", res = 300)manhattan(subset(output_Mo15.125_Clean_qqman_mm, CHR==12),main="Chr12" ,xaxt="n", genomewideline = FALSE, suggestiveline = FALSE) # remove axis labelslabel_axis=c("0Mb","5Mb","10Mb","15Mb","20Mb","25Mb","30Mb") # store new axis labels in "label_axis"axis(1, at=c(0,5000000,10000000,15000000,20000000,25000000,30000000), labels=label_axis) # display new axis labelsdev.off()write.table(results(data_Mo15.125_Cleanbinary.mm),"Mo15.125_Clean/Mo15.125_Clean_mm_correction.txt")Mo15.125_examination_mm<- read.table("Mo15.125_Clean/Mo15.125_Clean_mm_correction.txt")Mo15.125_top_snps_mm <- (Mo15.125_examination_mm[order(Mo15.125_examination_mm$Pc1df),c(1,2,4,5,11)])head(Mo15.125_top_snps_mm)#write a table for top 1000snps after mm correction sorted by chromosome. SNP table was use to identify peak SNP locationsMo15.125_top_1000snps_mm <- Mo15.125_top_snps_mm[1:1000,]write.table(Mo15.125_top_1000snps_mm[order(Mo15.125_top_1000snps_mm$Chromosome),],"Mo15.125_Clean/Mo15.125_Clean_mm_correction_top_1000snps.txt")
